# Analysis of circulating protein aggregates as a route of investigation into neurodegenerative disorders

**DOI:** 10.1093/braincomms/fcab148

**Published:** 2021-07-09

**Authors:** Rocco Adiutori, Fabiola Puentes, Michael Bremang, Vittoria Lombardi, Irene Zubiri, Emanuela Leoni, Johan Aarum, Denise Sheer, Simon McArthur, Ian Pike, Andrea Malaspina

**Affiliations:** 1 Centre for Neuroscience and Trauma, Blizard Institute, Queen Mary University of London, London E1 2AT, UK; 2 Proteome Sciences R&D GmbH & Co. KG, Frankfurt am Main 60438, Germany; 3 Department of Clinical Microbiology, Karolinska University Hospital, Stockholm 171 76, Sweden; 4 Centre for Genomics and Child Health, Blizard Institute, Queen Mary University of London, London E1 2AT, UK; 5 Institute of Dentistry, Blizard Institute, Queen Mary University of London, London E1 2AT, UK; 6 Proteome Sciences plc, Hamilton House, Mabledon Place, London WC1H 9BB, UK

**Keywords:** biomarkers, neurodegeneration, protein aggregates, proteomics, neurofilaments

## Abstract

Plasma proteome composition reflects the inflammatory and metabolic state of the organism and can be predictive of system-level and organ-specific pathologies. Circulating protein aggregates are enriched with neurofilament heavy chain—axonal proteins involved in brain aggregate formation and recently identified as biomarkers of the fatal neuromuscular disorder amyotrophic lateral sclerosis. Using unbiased proteomic methods, we have fully characterized the content in neuronal proteins of circulating protein aggregates from amyotrophic lateral sclerosis patients and healthy controls, with reference to brain protein aggregate composition. We also investigated circulating protein aggregate protein aggregation propensity, stability to proteolytic digestion and toxicity for neuronal and endothelial cell lines. Circulating protein aggregates separated by ultracentrifugation are visible as electron-dense macromolecular particles appearing as either large globular or as small filamentous formations. Analysis by mass spectrometry revealed that circulating protein aggregates obtained from patients are enriched with proteins involved in the proteasome system, possibly reflecting the underlying basis of dysregulated proteostasis seen in the disease, while those from healthy controls show enrichment of proteins involved in metabolism. Compared to the whole human proteome, proteins within circulating protein aggregates and brain aggregates show distinct chemical features of aggregation propensity, which appear dependent on the tissue or fluid of origin and not on the health status. Neurofilaments’ two high-mass isoforms (460 and 268 kDa) showed a strong differential expression in amyotrophic lateral sclerosis compared to healthy control circulating protein aggregates, while aggregated neurofilament heavy chain was also partially resistant to enterokinase proteolysis in patients, demonstrated by immunoreactive bands at 171 and 31 kDa fragments not seen in digested healthy controls samples. Unbiased proteomics revealed that a total of 4973 proteins were commonly detected in circulating protein aggregates and brain, including 24 expressed from genes associated with amyotrophic lateral sclerosis. Interestingly, 285 circulating protein aggregate proteins (5.7%) were regulated (*P* < 0.05) and are present in biochemical pathways linked to disease pathogenesis and protein aggregation. Biologically, circulating protein aggregates from both patients and healthy controls had a more pronounced effect on the viability of hCMEC/D3 endothelial and PC12 neuronal cells compared to immunoglobulins extracted from the same plasma samples. Furthermore, circulating protein aggregates from patients exerted a more toxic effect than healthy control circulating protein aggregates on both cell lines at lower concentrations (*P*: 0.03, in both cases). This study demonstrates that circulating protein aggregates are significantly enriched with brain proteins which are representative of amyotrophic lateral sclerosis pathology and a potential source of biomarkers and therapeutic targets for this incurable disorder.

## Teaser

Circulating protein aggregates in blood contain 4973 brain peptides including heavy chain neurofilaments and 24 amyotrophic lateral sclerosis-associated proteins. In amyotrophic lateral sclerosis, circulating protein aggregates express proteins involved in metabolism and proteosome and may be biologically active on endothelial cells.

## Graphical Abstract

**Graphical Abstract:**
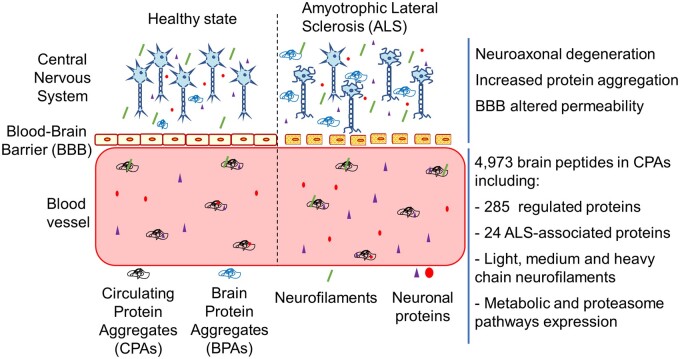

## Introduction

Disease progression in amyotrophic lateral sclerosis (ALS), a fatal and rapidly progressive neurodegenerative disorder, as in Alzheimer’s disease is characterized by the spread of pathological protein aggregation in the brain. In these neurodegenerative disorders, higher levels of aggregate-bound proteins like neurofilaments (Nfs) and tau are also detectable in biofluids.^1–3^ Change in biofluid levels of Nf in relation to rate of disease progression can be used for the clinical stratification of ALS.^4^^,^^5^ Once in a fluid environment, Nf may assemble into circulating protein aggregates (CPAs), similarly to protein aggregation seen in stress granule-like formations.^6^ We have recently demonstrated that CPAs extracted from the plasma of healthy individuals using ultracentrifugation (UC) and low-complexity binders are enriched with heavy chain neurofilament (NfH) and not with the light and medium isoforms (NfL, NfM).^7^ Protein composition of aggregates in circulation and not only of biofluids may therefore inform on brain protein composition and represent a viable source of potential biomarkers in a number of incurable neurodegenerative disorders.

Unlike whole plasma, the proteome of fluid-based aggregates has not been well documented. Our recent proteomic analysis of CPAs from neurologically healthy individuals has identified proteins involved in biological processes described in most neurodegenerative disorders, including cell structural and extra-cellular matrix proteins with a prion-like behaviour, or involved in inflammatory responses and in the phagosome pathway.^7–10^ Based on these data, we could speculate that plasma is a carrier of biologically active proteins, which are informative of the physiological and pathological state of organs. Indeed, previous studies have shown that proteins in circulation can influence the regenerative capacity of multiple tissues and organs in mice.^11^^,^^12^ Biologically active plasma proteins may also cause or facilitate the reported increase in blood–brain barrier permeability observed with ageing and in ALS.^13^

Both brain tissue and biological fluids have been reported to show an age-dependent increase in protein aggregation independent of any specific pathological state.^14^ The loss of solubility of proteins may relate to the reduction in chaperone and homeostatic functions of proteins like albumin, which is the most abundant in plasma.^15^ Age-associated changes in plasma protein composition have recently been investigated in a large cohort of individuals across a wide age range, leading to the identification of clusters of proteins whose expression is associated with an individual’s biological age and with the health status of different organs including brain.^16^^,^^17^ Since age is the main risk factor associated to the development of ALS and protein aggregation one of the main pathological hallmark of the disease,^14^^,^^18^ the study of the inherent aggregation propensity of proteins in a biofluids may be a novel route to investigate the pathobiology of neurodegeneration.

Here, we test the hypothesis that low-abundance brain proteins normally undetectable in the blood are compartmentalized within aggregate-like particles in blood and that these formations are enriched with proteins prone to aggregation and central to the pathobiology of a neurodegenerative disorder like ALS. We show that CPAs contain up to 5000 brain-derived proteins, a proportion of which are regulated in ALS and/or linked to ALS-risk genes. We also describe an ALS-specific pattern of NfH enterokinase proteolysis in CPAs and the biological effect that these formations have in brain and endothelial cell cultures.

## Materials and methods

### Patients and biological samples

Samples were collected from individuals with a diagnosis of ALS according to established criteria^19^ and from healthy controls (HC), enrolled in the ALS biomarkers study (REC n. 09/H0703/27). Participants had no known neurological comorbidities, nor were they affected by systemic or organ-specific autoimmune disorders (Supplementary Tables 1–3). Blood was drawn by venipuncture in ethylenediaminetetraacetic acid tubes, processed within 2 h by spinning at 3500 rpm for 10 min at 20°C and stored at −80°C.

Pre-central Gyrus brain tissue samples from two individuals affected by ALS (Brain1 and Brain2) obtained from The Netherlands Brain Bank (Netherlands Institute for Neuroscience, Amsterdam—www.brainbank.nl) were included in the study.

### Enrichment of protein aggregates

As previously reported, CPAs were enriched from plasma using a high concentration of detergent (Triton X-100) to dissolve vesicles and UC, to separate detergent-resistant particles^7^ (details in the Supplementary information). The same protocol was applied to brain samples after mechanical homogenization in 0.8 M NaCl, 1% Triton X-100, 0.1 M Ethylenediaminetetraacetic acid, 0.01 M Tris at pH 7.4 and proteinase inhibitor (cOmplete™, Merck).

### Quantification of CPAs protein content

The protein aggregate fractions dissolved in 8 M urea were tested using Pierce™ BCA Protein Assay Kit (ThermoFisher) for total protein quantitation.

### Transmission electron microscopy

A glow-discharged 400 mesh grid coated with carbon was incubated with a droplet of aggregate-enriched sample and after 10 s, the excess removed by carefully touching the grid edge with filter paper. Negative staining was obtained incubating the grid with a droplet of 2% w/v uranyl acetate. After washing with ddH_2_O, the grid was air-dried at room temperature and micrographs acquired by a JEOL JEM 1230 electron microscope.

### Circulating and brain protein aggregates protease digestion

Aggregate-enriched fractions were enzymatically digested using trypsin (V542A, Promega), α-Chymotrypsin (referred as Chymotrypsin in the text, C4129, Sigma), Calpain (208712, Millipore) and Enterokinase (11334115001, Roche). To minimize UC-induced protease resistance, to disrupt disulphide bonds and enhance cleavage sites accessibility, pellets were first re-suspended in 50 µl of each protease enzyme recommended buffer (PBS for Trypsin, 100 mM Tris HCl for Chymotrypsin, 50 mM Hepes–30 mM NaCl for Calpain and 50 mM Tris HCl for Enterokinase) and 5 µl of 0.5 M DTT was then added prior to sonication. Finally, each enzyme was added into the digestion reaction mix tube at a ratio 1:20 protease: total protein. 5.5 µl of 0.1 M CaCl_2_ were added to the chymotrypsin and calpain reaction mixes for enzyme activation as indicated by the manufacturers. Digestion mixes were subsequently incubated overnight at 37°C and later stopped adding 4× loading buffer (Fisher Scientific), dithiothreitol (DTT) and by heating at 95°C for 10 min. Resistance to proteases of NfH CPAs content was tested by western blotting.

### Western blotting

Proteins loaded onto gels were transferred after electrophoresis to a polyvinylidene difluoride membrane and then blocked with 5% skimmed milk in tris-buffered saline (TBS) 0.1% Tween-20 buffer (TBS-T 0.1%) at room temperature for 1 h. Incubation was performed overnight with primary antibody at 4°C and with secondary antibody for 1 h at RT. Membranes were washed with TBS-T 0.1% and incubated with enhanced chemiluminescence substrate. Imaging was undertaken using Image Lab (Bio-Rad) and bands volume measured using the ‘Volume Tools’ function in Image Lab and ‘Adj. Vol. (Int)’. We did not include immunodetection of loading control proteins in the Western blotting experiment as these were not considered appropriate for isolated CPAs with unknown content. In addition, most of the loading controls routinely used in Western Blotting experiments including Beta Actin, Beta Tubulin, Cyclophilin and GAPDH may undergo differential regulation as previously shown in ALS.^20–22^ From the protein content we have deducted correction factors and adjusted protein and NfH content as reported in the Supplementary information. Antibodies used in this study are listed in Supplementary Table 6. Blots’ full-size figures are available in the Supplementary information.

### Mass spectrometry-based proteomics

To study the protein composition of the enriched protein aggregate fractions from plasma and brain, we have first undertaken liquid chromatography coupled with tandem mass spectrometry (LC–MS/MS) analysis after in-gel trypsin digestion of pooled plasma samples CPAs from ALS and HC individuals as well as of brain protein aggregates (BPAs). We have then applied TMTcalibrator™ proteomics on individual ALS and HC CPA samples using ALS brain lysate as ion source (calibrant).

### In-gel trypsin digestion

Samples were loaded onto a gel for electrophoresis and gel bands were cut out. Subsequently, disulphide bonds reduction with DTT and alkylation with Iodoacetamide was performed. After washing and complete de-staining, each gel piece was rehydrated with a 50 mM ammonium bicarbonate (Ambic) solution and treated with 0.01 µg/µl Trypsin (V542A, Promega) during overnight (ON) incubation at 37°C. Tryptic peptides were recovered and each sample was freeze-dried in a vacuum centrifuge for LC–MS/MS analysis.

### TMTcalibrator™

The TMTcalibrator™ workflow was developed by Proteome Sciences plc^23–25^ to quantify low-abundance peptides in matrices with a high degree of biological complexity based on a tissue trigger/calibrant. Two ten-plexes were set up, each containing (i) lysate of two ALS brain tissue samples mixed 1:1 loaded at high concentration in 4 channels (calibrant samples) and (ii) CPAs from ALS patients and HC individuals (analytical samples; Supplementary Fig. 1). The triggering calibrant samples were prepared by dissolving brain tissues in SysQuant Buffer, removing the debris and mixing the two samples 1:1 (w/v):(w/v), while CPAs were obtained as described above. SysQuant Buffer was used to dilute 40 µg of each analytical sample total proteins and 840 µg of brain calibrant in each of the two ten-plexes. After reduction with DTT and alkylation with Iodoacetamide, desalting with SepPak tC18 was carried out and the calibrant divided into four different aliquots with a 1:4:6:10 volume ratio.

Dried samples were re-solubilised in 120 µl KH_2_PO_4_ and TMT reagents were added combining a specific tag for each sample (Supplementary Fig. 1) and reactions were stopped adding hydroxylamine to a final concentration of 0.25% (w/v). At this stage, the samples included in each ten-plex were merged and were fractionated by basic Reverse Phase using Pierce™ High pH Reversed-Phase Peptide Fractionation Kit (ThermoFisher Scientific), generating eight fractions for each of the two ten-plexes.

LC–MS/MS analysis was performed with replicate injections of approximately 40% of the total fraction volume using a Thermo Scientific™ Orbitrap Fusion Tribrid (Thermo Scientific) mass spectrometer coupled to an EASY-nLC 1000 (Thermo Scientific) system. The 16 basic Reverse Phase fractions were resuspended in 2% Acetonitrile (ACN), 0.1% formic acid. 12 µg from each basic Reverse Phase fraction was injected into a 75 µm × 2 cm nanoViper C18 Acclaim PepMap100 precolumn (3 µm particle size, 100 Å pore size; P/N 164705; Thermo Scientific). Peptides were separated at a flow rate of 250 nl/min and eluted from the column over a 5 h gradient starting with 0.1% formic acid in ACN (5-30% ACN from 0 to 280 min followed by 10 min ramping up to 80% ACN) through a 75 µm × 50 cm PepMap RSLC analytical column at 40°C (2 µm particle size, 100 Å pore size; P/N ES803; Thermo). After electrospray ionization, MS spectra ranging from 350 to 1500 m/z values were acquired in the Orbitrap at 120 k resolution and the most intense ions with a minimal required signal of 10 000 were subjected to MS/MS by HCD fragmentation in the Orbitrap at 30 k resolution. Protein identification was carried out with Thermo Scientific Proteome Discoverer 1.4.

### Bioinformatics

LC–MS/MS analysis generated 32 files that were processed by Proteome Sciences’ proprietary workflows for TMTcalibrator™ including the Calibrator Data Integration Tool (CalDIT), Feature Selection Tool (FeaST) and Functional Analysis Tool (FAT)^23^^,^^25^ (Supplementary Fig. 2). All raw spectra were searched against the human FASTA UniProtKB/Swiss-Prot using SEQUEST-HT and raw intensity values were measured through the TMT reporter ions.

### Aggregation propensity

To determine the propensity to aggregate of proteins identified as CPA components, we performed *in silico* analysis of protein size, isoelectric point (pI) and hydrophobicity using Uniprot—ExPASy (Compute pI/MW web tool and GRAVY score—http://www.gravy-calculator.de Accessed 08 July 2021). This analysis was undertaken comparing the ALS, HC CPA and BPA protein lists to the entire Uniprot human proteome (reviewed sequences only).

### IgG extraction from plasma and quantification

IgG extraction from the same plasma samples used for CPAs separation was carried out using Protein G Spin Columns (Thermo Scientific, UK) following the manufacturer’s protocol. The fraction of purified antibodies was determined measuring the relative absorbance of each fraction at 280 nm and the buffer exchanged into PBS using Amicon Ultra centrifugal filter with 100 kDa molecular weight (MW) cut-off (Millipore Merck, UK).

### PC12 viability

Undifferentiated PC12 cells were cultured in Dulbecco’s modiﬁed Eagle’s medium (Invitrogen, Paisley, UK) supplemented with 10% foetal calf serum (Invitrogen) and 10% horse serum (Sigma), 100 µg/ml streptomycin, 100 U/ml penicillin (Invitrogen) and incubated at 37° in a 5% CO_2_-humidiﬁed atmosphere. Differentiation was obtained by plating at a density of 3 × 10^5^ cells/well in 96-well plates (Nunc, ThermoFisher, UK) in Dulbecco’s modiﬁed Eagle’s medium (0.1% horse serum supplemented with nerve growth factor, 50 ng/ml). Cells were treated for 24 h at RT with CPAs dissolved in Urea 8 M or with IgG in medium. Viability was tested incubating with 0.5 mg/ml 3-(4,5-dimethylthiazol-2-yl)-2,5 diphenyltetrazolium bromide for 4 h. Supernatants were discarded and 200 µl DMSO added to solubilize formazan crystals. Colorimetric changes were measured at 590 nm (Synergy HT microplate reader). The percentage of cell viability was calculated as the absorbance of treated cells/absorbance of control.

### Endothelial cell viability

Human cerebromicrovascular endothelial cell lines hCMEC/D3 were maintained and treated as described previously.^26^^,^^27^ Cells were plated on plastic coated with 0.06 µg/cm^2^ calf skin collagen type I (Sigma, UK) and were cultured to confluency in complete endothelial cell growth medium MV2 (PromoCell GmbH, Germany). Following treatment for 24 h with aggregates or IgG in medium, cell number was estimated using the Prestoblue HS Cell Viability assay (ThermoFisher Scientific Ltd, UK) according to the manufacturer’s instructions and using a CLARIOstar fluorescence microplate reader (BMG Labtech Ltd, UK) with excitation and emission filters set to 560 nm and 590 nm, respectively.

### Statistical analysis

The enriched Kyoto Encyclopaedia of Genes and Genomes pathways obtained from the submission of ALS and HC protein lists to Webgestalt were assessed for statistical significance using the hypergeometric test.^28^ Principal component analysis (PCA) was used to study the variance of the data sets generated by the TMTcalibrator™ workflow. To determine regulated features (FeaST), LIMMA considered the following linear model: logRatio (ALS/HC) ≈ class + group + gender + progression rate + TMT batch. Multiple testing corrections and false discovery rate were obtained using the Benjamini–Hochberg procedure. For the Functional analysis (FAT), a two-sided *P*-value was generated by the Mann–Whitney U-test and the Benjamini–Hochberg method was used for multiple test correction. Expression values were normalized with other 1000 randomly selected background expression values. A minimum of three matched identifiers (e.g. gene names) were required for each term. Terms with an adjusted *P*-value < 0.3 were considered significant. Cell survival assays were evaluated by two-way ANOVA and Tukey HSD test. Non-parametric group analysis was performed using Kruskal–Wallis one-way test of variance on ranks with Dunn’s multiple comparison as post-test using GraphPad (v7). To test ALS versus HC proteolytic bands intensity difference, a ‘t-Test—Two-Sample Assuming Unequal Variances’ was performed. To test differences in aggregation propensity across proteomes, data were analysed for normality using the Shapiro–Wilk test.

### Study approval

A written informed consent was obtained from all ALS and HC participants enrolled in the ALS biomarkers study (REC n. 09/H0703/27). Written informed consent was obtained from donors of brain samples for the use of the material and clinical information for research purposes (Netherland Brain Bank: 2009/148).

### Data availability

All the raw files, searches and semi-quantitative data were stored into the ProteomeXchange Consortium via the PRIDE^29^ partner repository with the dataset identifiers PXD018938 and PXD018923.

## Results

### Study participants

Initial MS-based analysis of circulating CPAs was performed on 2 pools of plasma samples, one containing samples from three fast and three slow progressing ALS individuals and one from six HC individuals [ALS: 5 males (M), 1 female (F); HC: 3M, 3F; age range: ALS: 46.1–78.5; HC 51.8–62.9; Supplementary Table 1].

For the TMTcalibrator™ proteomic experiment, for protease digestion and for validation by western blot, CPAs extracted from plasma samples from ALS and HC cohorts (male/female ratio: 3:3; age range: ALS 60.2–68.8; age range: HC 60.6–68.3; Supplementary Tables 2 and 3) were tested individually.

### Extraction of CPAs and BPAs: qualitative analysis by TEM

**Figure 1:**
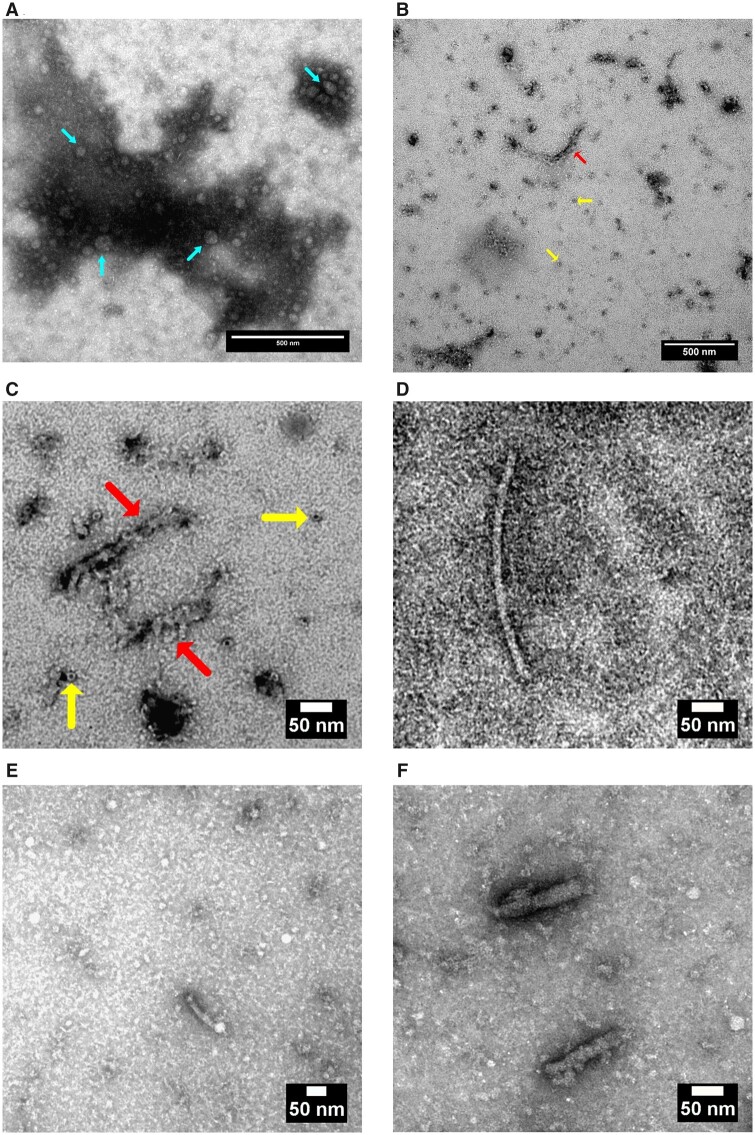
**Micrographs of circulating protein aggregates (CPAs) and brain protein aggregates (BPAs) taken by transmission electron microscopy.** (**A**) grid micrograph after CPAs sample loading showing an amorphous globular formation with adjacent and/or superimposed smaller rounded particles (which may be formed of lipoproteins; cyan arrows). (**B**) grid micrograph of BPAs (left-hand side) showing amorphous electron-dense as well as short filamentous and small round formations (red and yellow arrows, respectively). (**C**) Details of filamentous and of donut-like particles detected in BPA micrographs (red and yellow arrows, respectively). (**D, E, F**) Micrograph grids of CPAs showing 13–20 nm thick and 70–360 nm long fragments. Scale bar on the lower right-hand corner of each micrograph.

The efficiency of CPAs and BPAs extraction protocols was assessed using TEM to visualize the aggregate fractions after UC of plasma samples (3 HC and 3 ALS cases) and ALS brains. TEM revealed the presence of (macromolecular) amorphous electron-dense particles of different size, in both CPAs and BPAs (Fig. 1A and B, respectively). With the same extraction protocol, in CPA but not in BPA grids, it was possible to appreciate small, round (few nm diameter) particles close or superimposed to the bigger, globular and more electron-dense bodies. As previously reported, these formations may represent micelles composed of lipids, detergents or lipoproteins^30^^,^^31^ (Fig. 1A and B), such as very low-density, low-density and high-density lipoproteins. Both lipoproteins and biochemical pathways linked to their metabolism were in fact found significantly regulated in the CPAs proteomic study (described below). Unlike the large, amorphous, globular appearance of CPA aggregates, some of the electron-dense formations in the BPA micrographs had filamentous and donut-like shapes (Fig. 1B and C), suggestive of contamination with ferritin of brain homogenates as previously reported.^32–34^ In ALS CPA grids only, it was possible to see filamentous fragments with a rough surface similar to those seen in BPAs (Fig. 1D–F).

### CPAs and BPAs composition: LC–MS/MS proteomics

LC–MS/MS after in-gel trypsin digestion was used to study protein aggregates enriched from ALS and HC pooled plasma samples as well as from ALS brains. Three hundred sixty-seven proteins were identified in ALS CPAs and 353 in HC CPAs (Supplementary Fig. 3). Two hundred sixty-four (57.9% of the total) proteins were expressed in both ALS and HC CPAs (defined as shared), while 103 (22.6%) were found only in ALS (defined as unique ALS) and 89 (19.5%) in HC CPAs (defined as unique Controls) (Supplementary Fig. 3; proteins listed in Supplementary Files Protein list). Functional analysis performed using Webgestalt for Kyoto Encyclopaedia of Genes and Genomes pathway enrichment identified the proteasome as the most significantly represented feature in ALS (*P* = 0.028; four genes matched this category), while the glycolysis/gluconeogenesis pathway (*P* = 0.009; seven genes matched), pentose phosphate pathway (*P* = 0.003; five genes matched) and carbon metabolism (*P* = 0.008; eight genes matched) were significantly over-represented in HC. Proteins previously linked to ALS like NfH or TDP-43 were not detected. In brain BPAs we only identified 48 unique protein groups, including the three NF isoform proteins.

Five proteins were identified in all aggregate types [actin cytoplasmic 1, tubulin alpha-4A chain isoform 2, clathrin heavy chain 1 isoform 2, collagen alpha-1 (VI) and plectin isoform 7; Supplementary Fig. 2; proteins listed in Supplementary Files Protein list]. In addition, one protein was seen in both ALS CPAs and BPAs (cytoplasmic dynein 1 heavy chain 1) and one in both HC CPAs and BPAs [collagen alpha-2 (VI), respectively]. These data suggest that aggregates formed in the brain and in the peripheral circulation contain proteins that are involved in the structure and function of axons, a significant percentage of which may be implicated in the pathogenesis of ALS.^35^ For example, several variants of the gene encoding α-tubulin TUBA4A have been reported to have a destabilizing effect on the microtubule network, reducing the repolymerization capability of these proteins which is critical to the maintenance of axonal function and integrity.^36^

### Aggregation propensity

**Figure 2:**
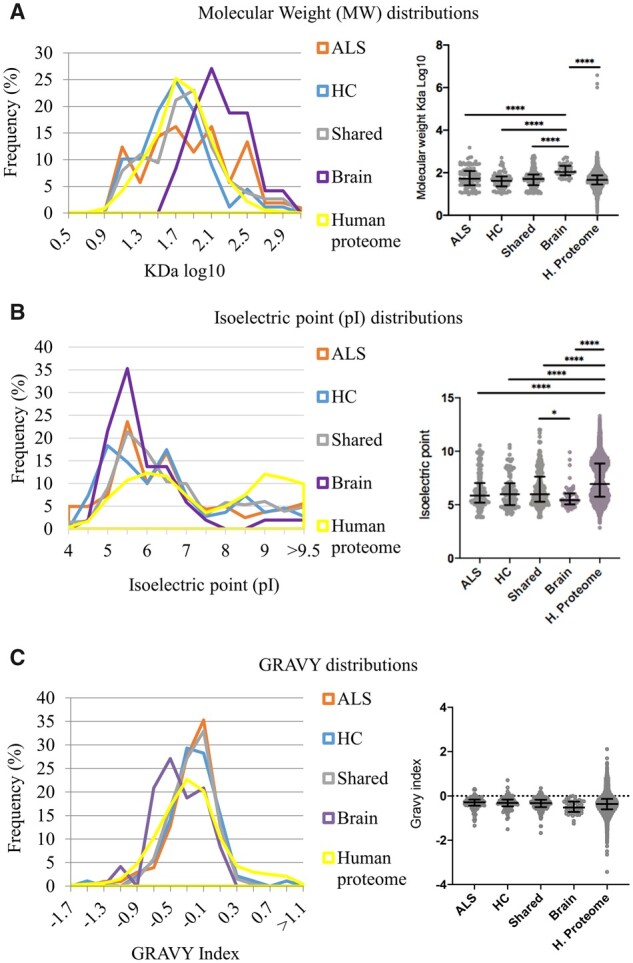
**Aggregation propensity of proteins in blood and brain aggregates from ALS and HC compared to the Human proteome.** Molecular weight (MW) (**A**), isoelectric point (pI) (**B**) and hydrophobicity (GRAVY index) (**C**), known to affect aggregation propensity of proteins, are compared across those expressed only in ALS and HC CPAs (ALS and HC, respectively), those shared between ALS and HC CPA datasets (shared), those within brain aggregates (brain) and in the entire human proteome. The distribution plots show the dispersion of the samples with relative frequency, while the violin plots show median and interquartile ranges of the measures. Statistical analysis was performed using one-way ANOVA, Kruskal–Wallis test with Dunn's multiple comparison as post-test for group analysis (*: *P* = 0.0251; ****: *P* < 0.0001); proteins listed in Supplementary Files Protein list.

The brain and plasma aggregate protein lists generated by LC–MS/MS were studied to test the propensity to aggregation of proteins in each dataset. The distribution of protein size or MW, pI and hydrophobicity, expressed as GRAVY index, was analysed in each proteome dataset with the whole human proteome as reference.^37^ BPA proteins had a significantly higher MW (*P* < 0.0001) compared to the other protein groups (ALS and HC shared and Human proteome, Fig. 2A), while pI was significantly lower in all CPA datasets compared to the Human proteome (*P* < 0.0001; Fig. 2B). Despite minimal overlap in protein composition between CPAs and BPAs, aggregation propensity in the two aggregate types and in the human proteome was similar when measured by GRAVY index, which takes into account the average hydropathy of peptides according to its aminoacidic composition (Fig. 2C).

### CPAs protease digestion and NfH resistance

**Figure 3:**
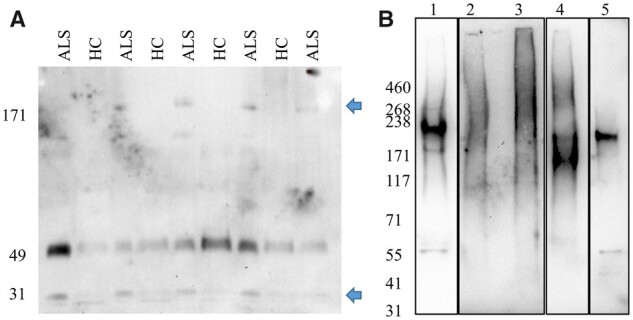
**Western blot analysis of neurofilament heavy chain (NfH) within circulating (CPAs) and brain (BPAs) protein aggregates after proteases digestion.** (**A**) shows a 49 kDa band uniformly expressed across samples and additional 171 and 31 kDa bands only in ALS patients (blue arrows). (**B**) Undigested NfH in ALS brain protein aggregates (BPAs; lane 1) and after digestion with chymotrypsin (lane 2), enterokinase (lane 3), calpain (lane 4) and brain lysate lane 5. To maximize band visualization, time exposure was for lane 1 at 10.1 s, lane 4 and 5 at 58.4 s and lane 2 and 3 at 278.8 s. See Supplementary information for full-size blots.

Resistance to protease digestion has been described as a key feature of pathological aggregation of proteins in conditions like prion disease.^38^ We have previously shown that neurofilament heavy chain (NfH) is found in blood CPAs.^7^^,^^39^ As NfH is constitutively expressed in protein aggregates from ALS brain and has been linked to the pathogenesis of the disease, we looked at NfH protease resistance in CPAs from ALS and compared its digestion profile to that in HC CPAs and BPAs (Fig. 3 and Supplementary Fig. 4).

Western blot analysis of NfH in plasma CPAs before digestion detected three bands at 460, 268 and 41 kDa as previously reported (Supplementary Fig. 4).^7^ The sum of all NfH band intensities (SUM) was (not significantly) higher in the ALS group compared to HC. The ratio between the intensity of the 460 kDa band and the NfH SUM intensity (460/SUM) was higher in HC, while the ratio between the intensities of the 268 and 460 bands (268/460) was significantly higher in ALS (Supplementary Fig. 4).

Treatment with trypsin or chymotrypsin showed an almost complete digestion of NfH in both ALS and HC, with the exception of a residual 41 kDa band present in a minority of samples (data not shown). After calpain digestion, there was a different pattern of immunoreactivity for each CPAs sample with the exception of 58 and 41 kDa bands evenly detected in all samples (Supplementary Fig. 4). Enterokinase digestion resulted in a 49 kDa band in all samples with equal expression in ALS and HC (Fig. 3A). All ALS samples showed bands at 171 and 31 kDa not seen in HC samples (Fig. 3A).

NfH in BPAs showed low or no resistance to digestion with all three enzymes (Fig. 3B). Chymotrypsin and enterokinase digestions (Fig. 3B, lane 2 and 3, respectively) generated no distinct bands but a faint smear at higher MW than the NfH bands detected in undigested brain and brain lysate (Fig. 3B, lane 1 and 5, respectively).

### TMTcalibrator™: brain-derived proteins in CPAs

The observed lack of similarity in the composition of brain and plasma aggregates may relate to the limits of proteomic techniques, whereby low abundance (brain-derived) proteins may be masked by those with high abundance and detection confounded by the presence of post-translational modifications. To address these confounds and gather more information on the potential enrichment of brain-derived proteins in circulating aggregates, we have undertaken further proteomics using a TMTcalibrator™ workflow, where tissue lysate was used to enhance detection of brain-derived proteins in CPAs.^23–25^

Inclusion of the brain trigger in TMT^®^ 10plex experiments dramatically increased the numbers of unique protein identifications from CPAs. In total, 4973 proteins were identified in all TMT^®^ channels, including the three Nfs protein isoforms [Nf Light (NfL), Nf Medium (NfM) and Nf Heavy (NfH)]. Nf were found at a relatively higher concentration in ALS compared to HC samples [log2-fold change ALS/HC (logFC) = 0.093, 0.181 and 0.298, respectively] but none was significantly regulated (*P* = 0.40, 0.16 and 0.06, respectively). There were 285 proteins (5.7%) showing a statistically significant regulation (*P* < 0.05). Of these, 158 were more expressed in HC (logFC < 0) with an average fold-change (FC) of −0.667, while 127 in ALS (logFC > 0) with an average FC of 0.703. The protein list with the 285 proteins was matched with an ALS gene list obtained from the MalaCards database, an integrated repository of human diseases and their annotations. Proteins encoded by 24 ALS-associated genes were identified including Fused in Sarcoma RNA-binding protein (FUS) which was found to be significantly regulated in ALS CPAs (*P* = 0.00696) (Table 1).

**Table 1:** ALS risk genes included in the list of proteins identified by the TMTcalibrator™ workflow in CPA from ALS and HC and reported in the gene classifiers MalaCards Human Disease Database (https://www.malacards.org/card/amyotrophic_lateral_sclerosis_1#RelatedGenes-table Accessed 08 July 2021) (Database, n.d.). Among a total of 38 ALS elite genes reported in the MalaCards Human Disease Database (those more likely to cause the disease), 24 were detected in the list of proteins generated by the TMTcalibrator™ experiment.

^A^Gene	^B^Uniprot ID	^C^Protein name	^D^Unique peptides	^E^logFC	^F^ *P*-value
**FUS**	P35637-2	Isoform Short of RNA-binding protein FUS	5	0.564	6.96e^**-03**^
**NEFH**	P12036	Neurofilament heavy polypeptide	27	0.298	6.36e^**-02**^
**OPTN**	Q96CV9	Optineurin	11	−0.243	9.58e^**-02**^
**UNC13A**	Q9UPW8	Protein unc-13 homolog A	11	−0.326	9.75e^**-02**^
**PON2**	Q15165-3	Isoform 3 of Serum paraoxonase/arylesterase 2	1	0.292	1.46e^**-01**^
**ANG**	P03950	Angiogenin	1	−0.595	1.67e^**-01**^
**CHMP2B**	Q9UQN3	Charged multivesicular body protein 2 b	1	0.492	1.90e^**-01**^
**VCP**	P55072	Transitional endoplasmic reticulum ATPase	65	−0.514	2.16e^**-01**^
**ATXN2**	Q99700-2	Isoform 2 of Ataxin-2	2	−0.445	2.51e^**-01**^
**ANXA11**	P50995-2	Isoform 2 of Annexin A11	16	0.150	3.01e^**-01**^
**SOD1**	P00441	Superoxide dismutase [Cu-Zn]	8	0.176	3.39e^**-01**^
**ERBB4**	Q15303-4	Isoform JM-B CYT-2 of Receptor tyrosine-protein kinase erbB-4	1	−0.284	3.70e^**-01**^
**TARDBP**	Q13148	TAR DNA-binding protein 43	2	0.180	3.85e^**-01**^
**SQSTM1**	Q13501	Sequestosome-1	2	−0.280	4.00e^**-01**^
**MATR3**	P43243	Matrin-3	16	0.127	4.03e^**-01**^
**PFN1**	P07737	Profilin-1	14	0.136	4.08e^**-01**^
**VAPB**	O95292	Vesicle-associated membrane protein-associated protein B/C	10	−0.094	4.31e^**-01**^
**EPHA4**	P54764	Ephrin type-A receptor 4	13	−0.082	5.37e^**-01**^
**PON1**	P27169	Serum paraoxonase/arylesterase 1	14	0.123	6.07e^**-01**^
**TAF15**	Q92804-2	Isoform Short of TATA-binding protein-associated factor 2 N	3	0.067	6.99e^**-01**^
**UBQLN2**	Q9UHD9	Ubiquilin-2	4	−0.065	6.99e^**-01**^
**HNRNPA1**	P09651–3	Isoform 2 of Heterogeneous nuclear ribonucleoprotein A1	8	−0.066	7.50e^**-01**^
**DCTN1**	Q14203-6	Isoform 6 of Dynactin subunit 1	2	0.020	9.34e^**-01**^
**TBK1**	Q9UHD2	Serine/threonine-protein kinase TBK1	5	−0.004	9.86e^**-01**^

A: the gene symbol used to represent a gene.

B: Uniprot database protein identifier.

C: protein full name recommended by Uniprot.

D: number of peptide sequences unique to a protein group.

E: relative quantification with value expressed as log2(ALS/HC) intensities.

F: statistical significance for differential regulation between ALS and HC experimental groups.

**Figure 4:**
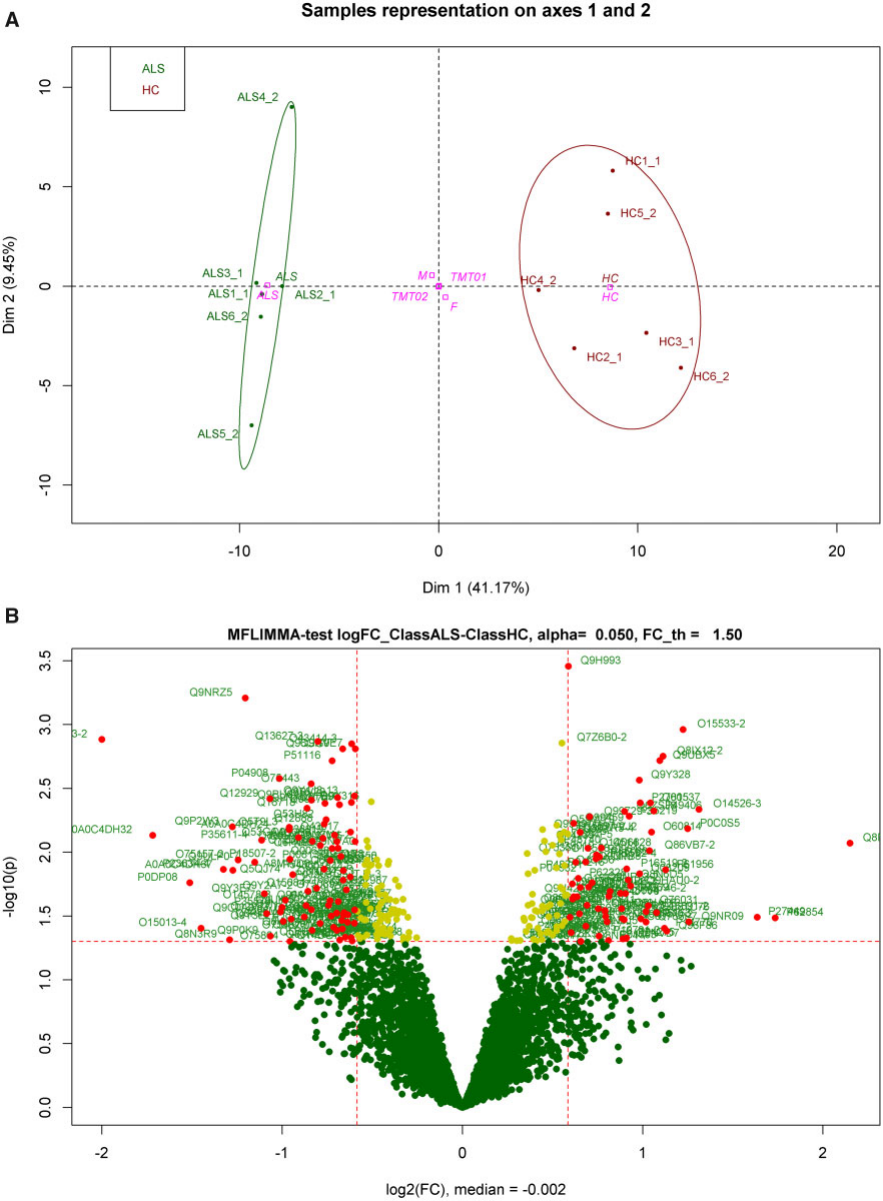
**TMTcalibrator™ proteomic analysis.** (**A**) Principal component analysis (PCA) showing a separation between the ALS and HC experimental groups regulated features at protein level. Dimension 1 or the variance between the two experimental groups (ALS and HC) is 41.17% of the entire variance; dimension 2 or variance between 10plexes (TMT01 and TMT02) is 9.45% of the entire variance. (**B**) The volcano plot shows the distribution of the proteins identified by TMT proteomic study according to their fold change (FC) expressed as log2 (fold change ALS/HC) (logFC) in the *x*-axis and according to *P*-value expressed as –log10 (*P*-value) in the *y*-axis. Protein groups were considered regulated if *P*-value < 0.05 and logFC < −0.58 or > 0.58. Red dots are regulated features, yellow dots are features with a significant *P*-value (*P* < 0.05) and logFC between −0.58 and 0.58 while green dots are not regulated protein groups (*P* > 0.05). Uniprot IDs are reported beside the dots with significant *P*-value.

PCA of the proteomic data after correction for the TMT^®^ batch-effect identified the sample origin (ALS or HC) as the strongest component (41.17%) of the total variance in the data matrix (Fig. 4A). The marked separation between ALS and HC identified by PCA was also observed in hierarchical clustering of the regulated proteins (Supplementary Fig. 5). Considering the CPAs enrichment of brain proteins (Fig. 4B), we speculate that circulating protein assemblies represent a good source of biomarkers for ALS, which may be difficult to detect in whole plasma analysis.

### Functional analysis

We have assessed the relevance of different biological terms in the proteomic data, based on their over-representation in the subset of regulated proteins.^23^^,^^25^ Within the top ten regulated biochemical pathways of the 69 identified (*P* < 0.05), five were involved in metabolism of lipoproteins (Supplementary Table 7). Several authors have already described that metabolism in ALS is switched from sugars and carbohydrates to lipids use.^40–42^ We also took a protein-centric approach, looking at the most highly regulated features (unique peptides ≥ 2, LogFC < −0.693 or > 0.693, *P*-value < 0.05) to evaluate other pathways potentially involved with aggregation and neurodegeneration, and obtain viable molecular targets for patients stratification. From this, we identified 48 proteins (Supplementary Table 8) included in four regulated biochemical pathways: metabolism of carbohydrates (*P* = 0.0099), glycosaminoglycans metabolism, lysosome (*P* = 0.0015), synthesis of phosphatidic acid (*P* = 0.0184) and wnt signalling pathway (*P* = 0.0337). Glycosaminoglycans are involved in protein aggregation and prion protein diffusion.^43–51^ Lysosome activity changes have been described in ALS and linked to the C9orf72 gene repeat expansions,^52–56^ while synthesis of phosphatidic acid and related phospholipids, including phosphatidylcholine and phosphatidylethanolamine, have been linked to ALS and prion disease pathogenesis.^57^^,^^58^

### Analysis of regulated proteins by immunodetection

To evaluate our mass-spectrometry results using an orthogonal technique, we elected to use an immunoassay (Western blot) strategy, as this may ultimately be more suitable for mass testing. However, as our initial data relate to brain-triggered peptides from digested proteins, we had some concerns as to whether antibodies would recognize the same proteins in isolated CPAs.

**Figure 5:**
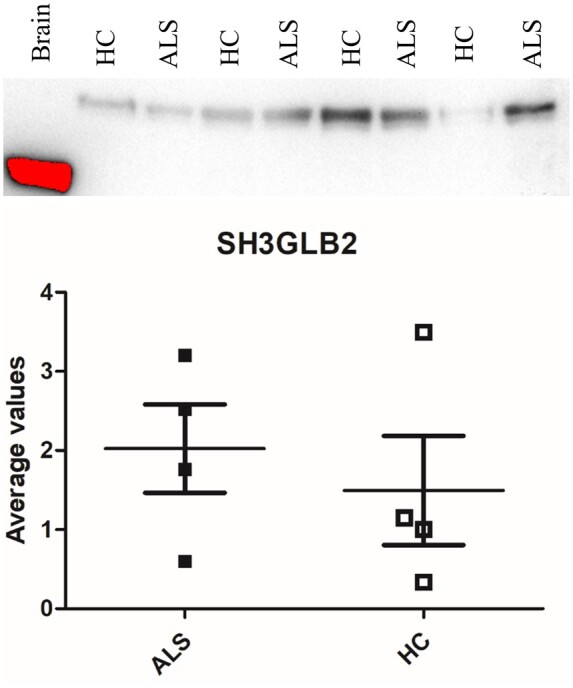
**Western blot analysis of Endophilin-B2 (SH3GLB2) in plasma CPAs from ALS patients and healthy controls.** Samples were normalized to HC density and the average values with relative standard deviation for the ALS (*n* = 4) and Control (*n* = 4) groups were plotted onto the chart. A brain lysate sample is also included (1st lane, red band, indicating signal saturation) which showed an endophilin-B2 band at a lower molecular weight than the bands detected in CPAs. Immunodetection confirmed the SH3GLB2 higher level of expression in the ALS CPAs compared to control (logFC = 0.34), but no statistically significant regulation (*P* = 0.57). As stated in the materials and methods section, no loading control was included for lack of constitutively expressed proteins in CPAs, as well as differential regulation presented in ALS literature for those proteins normally used in plasma and serum western blotting (e.g. albumin, transferrin, etc.). See Supplementary information for full-sizeblots.

We set up Western blot protocols for six of the most strongly regulated CPA proteins belonging to ALS-relevant molecular pathways: Glypican-4 (GPC4), Fibromodulin (FMOD), Biglycan (BGN), Cation-dependent mannose-6-phosphate receptor (M6PR), Endophilin-B2 (SH3GLB2) and Protein DJ-1 (PARK7). CPAs extracted from ALS patients and HC, along with ALS brain lysate as reference, were used in the western blot analysis. SH3GLB2 showed the same trend of ALS versus HC protein regulation identified in the TMTcalibrator™ experiment, with a similar level of regulation [logFC = 0.34 in TMTcalibrator™ and log2(ALS/HC) = 0.437] in western blot analysis, though this latter result was not statistically significant (Fig. 5). The remaining proteins showed a different trend of regulation compared to that obtained in the proteomic analysis (Supplementary Fig. 6) suggesting antibodies were detecting different forms of these proteins compared to a presumed assessment of total expression using bottom-up proteomics. Interestingly, SH3GLB2 and cation-dependent mannose-6-phosphate receptor were detected at a higher MW in CPAs compared to brain lysate, supporting the hypothesis of a different PTM profile in tissues as opposed to fluids which may affect the state of aggregation and protease digestion.

### Aggregation propensity of the brain-derived proteins in CPAs

We evaluated aggregation propensity of the CPA proteins identified by TMTcalibrator™ compared to the Human proteome and BPAs, using the reported physicochemical parameters. The analysis was performed on the entire TMT^®^ proteome dataset and on the 285 regulated proteins only, which were divided in two groups based on the level of differential expression: log(ALS/HC) > 0 and log(ALS/HC) < 0. There was no statistically significant difference among these three datasets for the parameters under investigation. However, the entire TMT and BPAs proteome datasets showed statistically significant higher MW and lower pI compared to the Human proteome (*P* < 0.0001), while for the GRAVY index, the BPA values were lower than the TMT and Human proteome (*P* = 0.0348 and 0.0224, respectively; data not shown).

### Cell survival assays

**Figure 6:**
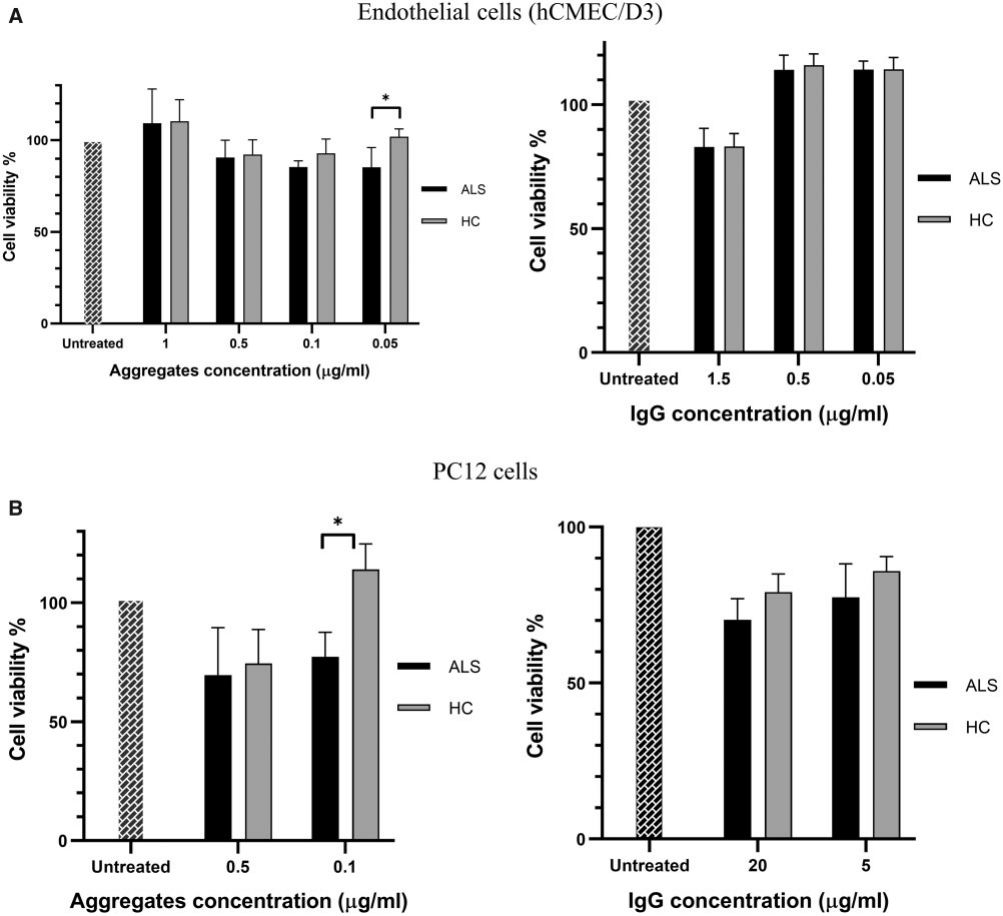
**Cell viability after treatment with aggregates, solubilized aggregates and immunoglobulins extracted from plasma samples.** The figure shows the percentage of endothelial (hCMEC/D3) and PC12 living cells (**A** and **B**) after treatment with different concentrations of CPAs and IgG from ALS and HC. Cells treated with ALS CPAs showed a statistically significant lower cell viability compared to HC CPAs treated cells at 0.05 µg/ml (*P* = 0.031; endothelial cells, **A**) and at 0.1 µg/ml (*P* = 0.029; PC12 cells, **B**). IgG had minor effect on all cell type viability with no difference between ALS and HC. CPA proteins were solubilized with 8 M urea before PC12 cells treatment. Significance was tested by two-way ANOVA and Tukey HSD test.

To test the biological effects of CPAs on living cells, human brain microvascular endothelial cells (hCMEC/D3) modelling human blood–brain barrier and PC12 neuron-like cells lines were treated with CPAs from ALS patients and HC and cell viability measured (Fig. 6). Total IgG were extracted from the same blood samples CPAs were separated from and used to treat the same cell lines. CPAs re-suspended in PBS were administered at defined concentrations to hCMEC/D3, while CPAs were pre-treated with 8 M urea before testing PC12 viability. Dissolution of aggregates by urea for PC12 cells was undertaken to evaluate the effect of CPA-containing proteins rather than the effect of their aggregated state. PC12 cell viability decreased at increasing concentration of CPAs (to 75% at 0.5 µg/ml; Fig. 6B), while endothelial cells showed the opposite trend, with the maximum effect on cell viability at the lowest concentration (0.05 µg/ml) and no effect at the highest concentration (1 µg/ml; Fig. 6A). ALS CPAs exerted higher toxicity (lower cell viability) at lower concentration (0.05 µg/ml) with endothelial cells and with PC12 cells (0.5 µg/ml) compared to HC CPAs (Fig. 6). IgG extracted from the same ALS and HC plasma samples showed reduction of endothelial cell viability to 80% and to between 70 and 85% in PC12 cells at 1.5 µg/ml of IgG, with no significant difference between ALS and HC (Fig. 6).

## Discussion

Using a TMTcalibrator™ proteomic approach we show, for the first time, that CPAs contain approximately 5000 unique proteins that are also expressed in ALS brain. CPAs obtained from individuals with ALS include products of translation of ALS risk genes such as FUS and SOD1 (Table 1) and a large number of proteins implicated in the proteasome system, an essential clearance mechanism of defective proteins.^59^^,^^60^ Plasma CPAs and specifically low concentration ALS CPAs, affect both endothelial and neuronal cells viability, showing a more pronounced biological effect than that observed using the immunoglobulin fraction extracted from the same plasma samples (Fig. 6).

The detection by proteomics in blood-borne CPAs of 285 low-abundance brain proteins showing a significant level of regulation (*P* < 0.05) in ALS compared to controls (Fig. 4, Supplementary Fig. 5) widens the potential for biomarkers discovery based on the analysis of proteins compartmentalized in aggregates. The density of brain proteins within aggregates can only be appreciated using the TMTcalibrator™ workflow, which is able to analyse tissues and fluids in the same experiment. This technique enhances detection of low-abundance fluid proteins that are also expressed in brain, most of them undetectable using standard proteomics or immunodetection.^7^ For the identification of the same protein targets, immunoassays may suffer from competition of naturally occurring autoantibodies causing epitope sequestration in aggregates and immunocomplexes as recently shown for Nfs.^5^^,^^39^ Unlike TMTcalibrator™ enhanced detection based on an internal tissue calibrant, standard MS-based proteomics would, in turn, lack the sensitivity to discriminate low concentration against more abundant proteins.

The use of an orthogonal technique of immunodetection to reproduce and validate proteomic data is expected to strengthen the biological significance of any observation based on mass spectrometry. We believe that the use of CPAs as source of biomarkers in our study may limit the use of confirmatory immunoassays like western blotting to reproduce the results obtained by proteomics for the following reasons: (i) enhanced detection of brain proteins by use of a tissue trigger may lack homologous peptides with fluid-specific modifications that are the main targets of antibodies, and (ii) proteins may not be completely dissolved and remain sequestered within aggregates, thus reducing the protein epitopes interaction with the assay antibodies. These phenomena may skew detection in western blot producing results that differ from those obtained by proteomics. In our case, failure to detect a reproducible expression patterns of selected proteins in the same aggregates using two separate methodologies may also relate to the small number of samples employed in the experimental procedures. Nevertheless, knowledge of differentially expressed proteoforms related to post-translational modification and/or bioavailability may serve to further enhance our understanding of biomarker relevance and support the production of new antibodies with the required specificity.

The method of aggregates separation used in our study does not allow the analysis by proteomics of the supernatant from processed samples, due to detergent contamination following CPAs extraction. To obtain suitable supernatant in the aggregate extraction process, we have previously used low complexity binders for aggregate separation from biofluid. The resulting aggregate fraction had a substantially different protein composition to the one obtained by UC, the method of choice of the current study.^7^ It is therefore not possible to compare the protein profile of the aggregates and fluid components of the same plasma sample. To circumvent this problem, we have recently used two separate proteomic workflows, including brain-enhanced TMT proteomics, to study the immunological response and the plasma/brain proteome in phenotypic variants of ALS.^25^ In whole plasma, we only identified nine ALS-associated genes out of the 24 identified in our study of CPAs (including Profilin-1 and 2 of Heterogeneous nuclear ribonucleoprotein A1). We could therefore speculate that aggregates in the blood are more enriched with neuron-derived and disease-specific proteins compared to the fluid component of plasma, making them a more desirable target in the search for ALS biomarkers. When compared to a healthy state, the use of brain-enhanced TMT proteomics and the functional analysis of the plasma CPAs proteome from ALS individuals reveal other important pathological hallmarks of ALS, including changes in the proteasome-dependent protein degradation and in energy metabolism pathways.^59–62^ The TMTcalibrator™ dataset contains regulated features also known to be involved in ALS pathology, including lysosome as well as lipoprotein and glycosaminoglycan metabolisms^40^^,^^41^^,^^52^^,^^55^^,^^56^^,^^59–62^ (Supplementary Table 7). To our knowledge, changes in proteasome activity in ALS have so far been shown in such detail only in brain, spinal cord and in neuronal cell lines, but not systemically or more specifically in blood.^59^^,^^60^

The analysis of the physicochemical properties of protein aggregation in our proteomic datasets provides further insight into protein behaviour in different molecular environments and in relation with a disease like ALS. When MW and pI are taken into account, we observe that the tissue or fluid of origin is the main contributors to the differences in the proteome chemical properties observed across the aggregate types and not the presence or absence of ALS (Fig. 2). When the whole human proteome is included in the analyses as reference, it is possible to see how proteins in the aggregated state are distinguishable from the whole set of human proteins, regardless of their tissue or fluid of origin or presence or absence of ALS.

With regard to proteolytic properties of aggregates, we have identified clear differences after enterokinase digestion between ALS and HC CPAs, with the presence of specific NfH digestion fragments at 171 kDa and at 31 kDa only in ALS samples (Fig. 3A). While a trend for NfH proteolytic fragment over-expression in ALS CPAs compared to HC is visible in our experimental data, our study lacks sufficient samples to establish this observation as previously reported.^5^^,^^63^ An extension of this preliminary finding using a larger number of samples will be needed to compare our observation to previously reported data on the same Nf isoform enterokinase digestion pattern in a different experimental context.^64^

We have tested the potential for CPAs to serve as a pathogenic seed using viability of (PC12) neuronal and (hCMEC/D3) endothelial cell lines following treatment with ALS and HC CPAs (Fig. 6). In contrast to treatment with enriched immunoglobulin fractions, that are reported to have organ-level^16^^,^^17^ effects, only CPAs treatment effected a detectable toxic effect on PC12 neurons and hCMEC/D3 endothelial cells. Furthermore, there was a clear ALS-specific change in cell viability when CPAs are administered at a relatively low concentration (Fig. 6). It is not possible to explain how this effect comes into play upon exposure to low-concentration CPAs, and whether the ALS-specific effect relates to a particular composition and/or conformation of aggregates which may be concentration-dependent. We can only speculate that smaller oligomeric complexes exert greater toxicity on cells compared to a larger hetero-aggregates, which are likely to be the predominant forms in higher concentration solutions. This would certainly be consistent with studies on amyloid particles in Alzheimer’s disease which show that larger size assemblies are less toxic than lower-ordered oligomers.^65^ The use of TEM to test the efficiency of CPAs separation confirms the presence of particles of both globular and filamentous appearance, similar to those observed in BPAs (Fig. 1). Further investigation by TEM may be needed to evaluate whether the ALS-specific CPAs effect on cells relate to a particular conformation, size or composition of aggregates. Based on these in-vitro observation, it is possible to speculate that CPAs may well be the proteinaceous component in blood that is ultimately responsible for the blood–brain barrier damage that has been reported in ALS.^13^

To date, there is no evidence in the literature of investigations into non-membrane bound particles in the blood of patients with a neurodegenerative disorder and their potential use in biomarkers discovery. Our data indicate that CPAs represent a new source of biomarkers enriched with brain proteins, including disease-relevant proteins, that can become regulated under pathological condition. These aggregates appear biologically active as they affect endothelial and neuronal cell viability when administered to cell culture. Further investigation on the nature of these particles will be required to confirm and strengthen this initial finding, including a more extensive comparison with brain aggregates and analysis of the biochemical characteristics in a larger subset of individuals, using more effective and user-friendly methods of CPAs extraction.

## Supplementary material

Supplementary material is available at *Brain Communications* online.

## Supplementary Material

fcab148_Supplementary_Data

## Abbreviations

ALS =: amyotrophic lateral sclerosis
BPAs =: brain protein aggregates
CPAs =: circulating protein aggregates
F =: female
FC =: fold-change
FUS =: fused in sarcoma RNA-binding protein
HC =: healthy controls
LC–MS/MS =: liquid chromatography coupled with tandem mass spectrometry
M =: male
MS =: mass spectrometry
MW =: molecular weight
Nfs =: neurofilaments
NfH =: heavy chain neurofilaments
NfL =: light chain neurofilaments
NfM =: medium chain neurofilaments
PCA =: principal component analysis
pI =: isoelectric point
TBS =: tris-buffered saline
TEM =: transmission electron microscopy
TMT =: Tandem Mass Tag
UC =: ultracentrifugation
