# Supplementary material for: Analysis of circulating protein aggregates as a route of investigation into neurodegenerative disorders
Source: Brain Commun. 2021 Jul 9;3(3):fcab148. doi: 10.1093/braincomms/fcab148 (PMC8361415; doi:10.1093/braincomms/fcab148)
Supplement: fcab148_Supplementary_Data [file fcab148_supplementary_data.zip › Original Submission(2).pdf]

OXFORD UNIVERSITY PRESS | Brain Communications

Analysis of circulating protein aggregates reveals pathological hallmarks of amyotrophic lateral sclerosis

Journal:	*Brain Communications*
Manuscript ID	BRAINCOM-2020-313
Manuscript Type:	Original Article
Date Submitted by the Author:	10-Oct-2020
Complete List of Authors:	Adiutori, Rocco; Blizard Institute of Cell and Molecular Science, Neuroscience and Trauma<br>Puentes, Fabiola; Blizard Institute of Cell and Molecular Science, Neuroscience and Trauma<br>Bremang, Michael; Proteome Sciences plc<br>Lombardi, Vittoria; Barts and The London School of Medicine and Dentistry Blizard Institute<br>Zubiri, Irene; Barts and The London School of Medicine and Dentistry Blizard Institute, Neuroscience and Trauma Center<br>Leoni, Emanuela; Proteome Science plc<br>Aarum, Johan; Karolinska University Hospital, Department of Clinical Microbiology<br>Sheer, Denise; Blizard Institute of Cell and Molecular Science, Centre for Genomics and Child Health<br>McArthur, Simon; Blizard Institute of Cell and Molecular Science, Institute of Dentistry<br>Pike, Ian; Proteome Science plc<br>Malaspina, Andrea; Blizard Institute of Cell and Molecular Science, Neuroscience and Trauma
Keywords:	biomarkers, neurodegeneration, protein aggregates, amyotrophic lateral sclerosis, proteomics, neurofilaments

SCHOLARONE™ Manuscripts

Analysis of circulating protein aggregates reveals pathological hallmarks of amyotrophic lateral sclerosis

Rocco Adiutori*[1], Fabiola Puentes[1], Michael Bremang[2], Vittoria Lombardi[1], Irene Zubiri[1], Emanuela Leoni[3], Johan Aarum[4], Denise Sheer[5], Simon McArthur[6], Ian Pike[2], Andrea Malaspina*[1]

Affiliation:

1: Centre for Neuroscience and Trauma, Blizard Institute, Queen Mary University of London, 4 Newark Street, London, E1 2AT, UK.

2: Proteome Sciences plc, Hamilton House, Mabledon Place, London, WC1H 9BB, UK.

3: Proteome Sciences R&D GmbH & Co. KG, Altenhöferallee 3, Frankfurt am Main, 60438, Germany.

4: Department of Clinical Microbiology, Karolinska University Hospital, Stockholm, 171 76 Sweden.

5: Centre for Genomics and Child Health, Blizard Institute, Queen Mary University of London, 4 Newark Street, London, E1 2AT, UK.

6: Institute of Dentistry, Blizard Institute, Queen Mary University of London, 4 Newark Street, London, E1 2AT, UK.

* Corresponding authors: Rocco Adiutori, Andrea Malaspina.

For Review Only

Abstract

Plasma proteins composition reflects the inflammatory and metabolic state of the organism and can be predictive of system-level and organ-specific pathologies. Circulating protein aggregates (CPA) are enriched with heavy chain neurofilaments (NfH), axonal proteins involved in brain aggregates formation and recently identified as biomarkers of the fatal neuromuscular disorder amyotrophic lateral sclerosis (ALS). Here we confirm CPA and brain protein aggregates (BPA) separation by ultracentrifugation using electron microscopy. We use mass spectrometry-based proteomics to evaluate the protein composition of CPA extracted from ALS and healthy controls (HC) pooled plasma samples. Further brain-enhanced TMTcalibrator™ proteomics is applied to estimate differential protein expression between ALS patients (n:6) and HC (n:6) and to characterize the brain-derived component of these circulating aggregates. We also test CPA and BPA proteins aggregation propensity and the resistance to proteases digestion by trypsin, chymotrypsin, calpain and enterokinase of NFH within aggregates. Finally, we study CPA biological effects on neuronal and endothelial cell lines. Electron microscopy confirms the presence of electron-dense macromolecular particles appearing as either large globular or as small filamentous formations after extraction by ultracentrifugation. CPA from ALS are enriched with proteins involved in the proteasome system while those from HC show a prominent expression of proteins involved in metabolism. NfH isoforms (460 and 268 KDa) within CPA show a differential expression in ALS compared to HC, while CPA enterokinase digestion in ALS generates 171 and 31 KDa fragments not seen in HC samples. Compared to the whole human proteome, proteins within CPA and BPA show distinct chemical features of aggregation propensity, which appear dependent on the tissue or fluid of origin and not on the health state. The use of a TMTcalibrator™ proteomics workflow reveals 4973 brain-derived low-abundance proteins in CPA, including products of translation of 24 ALS risk genes. 285 of CPA brain proteins (5.7%) are regulated in ALS ($p < 0.05$) and are part of biochemical pathways linked to ALS pathogenesis and aggregates formation. CPA from both ALS and HC have a higher effect on hCMEC/D3 endothelial and PC12 neuronal cells viability than immunoglobulins extracted from the same plasma samples. CPA from ALS exerts a more toxic effect than HC material on both cell lines at lower concentrations (p: 0.031 and 0.029, respectively). This study demonstrates that CPA are significantly enriched with brain proteins which are representative of ALS pathology and a potential source of biomarkers and therapeutic targets for this incurable disorder.

For Review Only

Keywords

Biomarkers, neurodegeneration, protein aggregates, amyotrophic lateral sclerosis, proteomics, neurofilaments

Introduction

Stratification of clinically heterogeneous neurodegenerative disorders into more homogeneous and predictable disease phenotypes is an essential pre-requisite for clinical trials (Bradley, 2012). As disease progression in Amyotrophic Lateral Sclerosis (ALS), a fatal neurodegenerative disorder, and in Alzheimer's disease may be linked to the spread of pathological protein aggregation in brain, the detection in biofluids of aggregate-bound proteins like neurofilaments (Nf), tau and beta amyloid, has proved a successful strategy for biomarkers discovery (Lee & Kim, 2015; Polymenidou & Cleveland, 2011). (Friedrich et al., 2010). Brain proteins like neurofilaments (Nf) can leak from pathological aggregates within neurons and axons into cerebrospinal fluid (CSF) and blood. In biofluids, proteins including Nf may assemble into circulating protein aggregates (CPA), similarly to what described for stress granule-like formations (Yang & Hu, 2016). Using ultracentrifugation (UC) and low-complexity binders to extract CPA from plasma of healthy individuals, we have recently shown that CPA are enriched with heavy chain neurofilament (NfH) and not with the light and medium isoforms (NfL, NfM) (Adiutori et al., 2018). We have also demonstrated that the change in biofluid levels of Nf in relation to the speed of disease progression can be used for the clinical stratification of ALS (C. H. Lu, Macdonald-Wallis, et al., 2015; C. H. Lu, Petzold, et al., 2015).

Both brain tissue and biological fluids have been reported to show an age-dependent increase in protein aggregation (Xia, Trasatti, Wymer, & Colon, 2016). The loss of solubility of proteins may relate to the reduction in chaperonal and homeostatic functions of specific proteins, like for example albumin, which is abundant in plasma (Finn, Nunez, Sunde, & Easterbrook-Smith, 2012). The increase of aggregation propensity of proteins with age is key to understand the pathobiology of neurodegeneration. In ALS, protein aggregation is an important pathological feature and age is probably the main risk factor to develop the disease (Niccoli, Partridge, & Isaacs, 2017; Xia et al., 2016). Age-associated changes in

For Review Only

plasma protein composition have recently been investigated in a large cohort of individuals of a wide age range, leading to the identification of clusters of proteins whose expression is associated with an individual's biological age and with the health status of different organs including the brain (Lehallier et al., 2019; Williams et al., 2019).

Unlike whole plasma, the proteome of biological fluids aggregates has not been well documented. Our recent proteomic analysis of CPA from neurologically healthy individuals has identified proteins involved in biological processes described in most neurodegenerative disorders, including cell structural and extra-cellular matrix proteins with prion-like behavior, or involved in inflammatory responses and in the phagosome pathway (A. McCombe & D. Henderson, 2011; Adiutori et al., 2018; Amor et al., 2014; Lyon, Wosiski-Kuhn, Gillespie, Caress, & Milligan, 2019). Based on the data reported above, we could speculate that plasma is a carrier of biologically active proteins, which are informative of the physiological and pathological state of organs. Indeed, previous studies have shown that proteins in circulation can influence the regenerative capacity of multiple tissues and organs in mice (Conboy et al., 2005; Villeda et al., 2011). Biologically active plasma proteins may also cause or facilitate the reported increase in blood brain barrier (BBB) permeability observed with ageing and in ALS (Garbuzova-Davis et al., 2012).

Here we test the hypothesis that those proteins compartmentalized within aggregate-like particles in blood may provide clues on the pathobiology of a neurodegenerative disorder like ALS and a new source of disease biomarkers, as already shown for brain protein aggregates. We show that CPA contain up to 5,000 brain-derived proteins, a proportion of which are regulated in ALS and/or linked to ALS-risk genes. We also describe an ALS-specific pattern of NfH enterokinase proteolysis in CPA and the biological effect that these formations have in brain and endothelial cell cultures.

For Review Only

Materials and Methods

Patients and biological samples

Samples were collected from individuals with a diagnosis of amyotrophic lateral sclerosis (ALS) according to established criteria (Ludolph et al., 2015) and from healthy controls (HC), enrolled in the ALS biomarkers study (REC n. 09/H0703/27). Participants had no known neurological comorbidities, nor were they affected by systemic or organ-specific autoimmune disorders (Supplementary Table 1-3).

Blood was drawn by venipuncture in EDTA tubes, processed within 2 hours by spinning at 3500 rpm for 10 minutes at 20 °C and stored at -80 °C.

Pre-central Gyrus brain tissue samples from two individuals affected by ALS (Brain1 and Brain2) obtained from The Netherlands Brain Bank (Netherlands Institute for Neuroscience, Amsterdam - www.brainbank.nl) were included in the study.

Enrichment of protein aggregates

As previously reported, circulating protein aggregates (CPA) were enriched from plasma using a high concentration of detergent (Triton X-100) to dissolve vesicles and protein complexes and ultracentrifugation (UC), to exploit the density of the detergent-resistant particles (Adiutori et al., 2018) (details in the Supplementary Material). The same protocol was applied to brain samples after mechanical homogenisation in 0.8 M NaCl, 1% Triton X-100, 0.1 M Ethylenediaminetetraacetic acid (EDTA), 0.01 M Tris at pH 7.4 and proteinase inhibitor (cOmplete™, Merck).

For Review Only

Quantification of CPA protein content

The protein aggregate fractions resuspended in 8M urea were tested using Pierce™ BCA Protein Assay Kit (ThermoFisher) for total protein quantitation.

Transmission Electron Microscopy (TEM)

A glow-discharged 400 mesh grid coated with carbon was incubated with a droplet of aggregates-enriched sample and after 10 seconds, the excess was removed by carefully touching to the grid edge with filter paper. Negative staining was obtained incubating the grid with a droplet of 2% w/v uranyl acetate (UA). After washing with ddH2O, the grid was air-dried at room temperature and micrographs acquired by a JEOL JEM 1230 electron microscope.

Circulating and brain protein aggregates protease digestion

Aggregates-enriched fractions were enzymatically digested using trypsin (V542A, Promega), α-Chymotrypsin (referred as Chymotrypsin in the text, C4129, Sigma), Calpain (208712, Millipore) and Enterokinase (11334115001, Roche). To minimize UC-induced protease resistance, for disrupting disulphide bonds and enhancing cleavage sites accessibility, pellets were first re-suspended in 50 µl of

each protease enzyme recommended buffer (PBS for Trypsin, 100mM Tris HCl for Chymotrypsin, 50mM Hepes - 30mM NaCl for Calpain and 50mM Tris HCl for Enterokinase) and 5 µl of 0.5 M DTT was then added prior to sonication. Finally, each enzyme was added into the digestion reaction mix tube at a ratio 1:20 protease:total protein. 5.5 µl of 0.1 M CaCl2 were added to the chymotrypsin and calpain reaction mixes for enzyme activation as indicated by the manufacturers. Digestion mixes were subsequently incubated overnight at 37°C and later stopped adding loading buffer 4X (Fisher Scientific), dithiothreitol (DTT) and by heating at 95°C for 10 minutes. Resistance to proteases of NfH CPA content was tested by western blotting and results adjusted to protein and NfH content (Supplementary Material).

For Review Only

Western blotting

Proteins loaded onto gels were transferred after electrophoresis to a polyvinylidene difluoride (PVDF) membrane and then blocked with 5% skimmed milk in Tris-Buffered Saline (TBS) 0.1% Tween-20 buffer (TBS-T 0.1%) at room temperature for 1 hour. Incubation was performed overnight with primary antibody at 4 °C and with secondary antibody for 1 hour at RT. Membranes were washed with TBS-T 0.1% and incubated with enhanced chemiluminescence substrate. Imaging was undertaken using Image Lab (Bio-Rad) and bands volume measured using the "Volume Tools" function in Image Lab and "Adj. Vol. (Int)". Antibodies used in this study are listed in Supplementary Table 6.

MS-based proteomics

To evaluate protein composition of the enriched protein aggregate fractions from plasma and brain, we have first undertaken LC-MS/MS analysis after in-gel trypsin digestion of pooled plasma samples (PPS) CPA from ALS and HC individuals as well as of brain protein aggregates (BPA). We have then applied TMTcalibrator™ proteomics on individual ALS and HC CPA samples using ALS brain lysate as ion source (calibrant).

In-gel trypsin digestion

Samples were loaded onto a gel for electrophoresis and gel bands were cut out. Subsequently, disulfide bonds reduction with DTT and alkylation with Iodoacetamide (IAA) was performed. After washing and complete de-staining, each gel piece was rehydrated with a 50 mM ammoniumbicarbonate (Ambic) solution and treated with 0.01 µg/µl Trypsin (V542A, Promega) during overnight (ON) incubation at 37

°C. Tryptic peptides were recovered and each sample was freeze-dried in a vacuum centrifuge for LC-MS/MS analysis.

TMTcalibrator™

TMTcalibrator™ workflow was developed by Proteome Sciences plc (Leoni et al., 2019; Russell et al., 2016; Zubiri et al., 2018) to quantify low-abundance peptides in matrices with a high degree of biological complexity. Two ten-plexes were set up, each containing 1) lysate of two ALS brain tissue samples mixed 1:1 loaded at high concentration in 4 channels (calibrant samples) and 2) CPA from ALS patients and HC individuals (analytical samples; Supplementary Fig. 1). The calibrant samples were prepared by dissolving brain tissues in SysQuant Buffer, removing the debris and mixing the two samples 1:1 (w/v):(w/v), while CPA were obtained as described above. SysQuant Buffer was used to dilute 40µg of total protein for each analytical sample and 840µg for the brain calibrant in each of the two ten-plexes. After reduction with DTT and alkylation with IAA, desalting with SepPak tC18 was carried out and the calibrant divided into four different aliquots with a 1:4:6:10 volume ratio.

Dried samples were re-solubilised in 120 µl KH2PO4 and TMT reagents were added combining a specific tag for each sample (Supplementary Fig. 1) and reactions were stopped adding hydroxylamine to a final concentration of 0.25% (w/v). At this stage the samples included in each ten-plex were merged and were fractionated by basic Reverse Phase (bRP) using Pierce™ High pH Reversed-Phase Peptide Fractionation Kit (ThermoFisher Scientific), generating eight fractions for each of the two ten-plexes.

LC-MS/MS analysis was performed in double-shot using a Thermo Scientific™ Orbitrap Fusion Tribrid (Thermo Scientific) mass spectrometer coupled to an EASY-nLC 1000 (Thermo Scientific) system. The 16 bRP fractions were resuspended in 2% ACN, 0.1% formic acid (FA), and then 12 µg from each was injected into a 75 µm × 2 cm nanoViper C18 Acclaim PepMap100 precolumn (3 µm particle size, 100 Å pore size; P/N 164705; Thermo Scientific). Peptides were separated at a flow rate of 250 nl/min and eluted from the column over a 5 hours gradient starting with 0.1% FA in ACN (5-30% ACN from 0 to 280 min followed by 10 min ramping up to 80% ACN) through a 75 µm × 50 cm PepMap RSLC analytical column at 40 °C (2 µm particle size, 100 Å pore size; P/N ES803; Thermo). After electrospray ionisation, MS spectra ranging from 350 to 1500 m/z values were acquired in the Orbitrap at 120 k

For Review Only

resolution and the most intense ions with a minimal required signal of 10,000 were subjected to MS/MS by HCD fragmentation in the Orbitrap at 30 k resolution. Protein identification was carried out with Thermo Scientific Proteome Discoverer 1.4.

Bioinformatics

LC-MS/MS analysis generated 32 files that were processed by Proteome Sciences' proprietary workflows for TMTcalibrator™ including the Calibrator Data Integration Tool (CalDIT), Feature Selection Tool (FeaST) and Functional Analysis Tool (FAT) (Leoni et al., 2019; Zubiri et al., 2018) (Supplementary Fig. 2). All raw spectra were searched against the human FASTA UniProtKB/Swiss-Prot using SEQUEST-HT and raw intensity values were measured through the TMT reporter ions.

Aggregation propensity

To study the chemical properties of the protein mix within the CPA which affect their propensity to aggregate, in-silico analysis of protein size, isoelectric point (pI) and hydrophobicity was undertaken using Uniprot – ExPASy (Compute pI/MW web tool and GRAVY score - http://www.gravy-calculator.de). This analysis was undertaken comparing the ALS, HC CPA and BPA protein lists to the entire Uniprot human proteome (reviewed sequences only).

IgG extraction from plasma and quantification

IgG extraction from the same plasma samples used for CPA separation was carried out using Protein G Spin Columns (Thermo Scientific, UK) following manufacturer's protocol. The fraction of purified antibodies was determined measuring the relative absorbance of each fraction at 280 nm and the buffer exchanged into PBS using Amicon Ultra centrifugal filter with 100 KDa molecular weight cut-off (Millipore Merck, UK).

PC12 viability

Undifferentiated PC12 cells were cultured in Dulbecco's modified Eagle's medium (Invitrogen, Paisley, UK) supplemented with 10% fetal calf serum (Invitrogen) and 10% horse serum (Sigma), 100 µg/ml streptomycin, 100 U/ml penicillin (Invitrogen) and incubated at 37° in a 5% CO_2-humidified atmosphere. Differentiation was obtained by plating at a density of 3×10^5 cells/well in 96-well plates (Nunc,

For Review Only

Thermofisher, UK) in Dulbecco's modified Eagle's medium (0,1% horse serum supplemented with nerve growth factor, 50 ng/ml). Cells were treated for 24 hours at RT with CPA dissolved in Urea 8M or with IgG in medium. Viability was tested incubating with 0.5 mg/ml 3-(4,5-dimethylthiazol-2-yl)-2,5 diphenyltetrazolium bromide (MTT) for 4h. Supernatants were discarded and 200 µl DMSO added to solubilize formazan crystals. Colorimetric changes were measured at 590 nm (Synergy HT microplate reader). The percentage of cell viability was calculated as the absorbance of treated cells/absorbance of control.

Endothelial cell viability

Human cerebromicrovascular endothelial cell lines hCMEC/D3 were maintained and treated as described previously (Hoyles et al., 2018; Weksler et al., 2005). Cells were plated on plastic coated with 0.06 µg/cm^2 calf skin collagen type I (Sigma, UK) and were cultured to confluency in complete endothelial cell growth medium MV2 (PromoCell GmbH, Germany). Following treatment for 24h with aggregates or IgG in medium, cell number was estimated using the Prestoblue HS Cell Viability assay (ThermoFisher Scientific Ltd., UK) according to the manufacturer's instructions and using a CLARIOstar fluorescence microplate reader (BMG Labtech Ltd., UK) with excitation and emission filters set to 560nm and 590nm respectively.

Statistics

The enriched KEGG pathways obtained from the submission of ALS and HC pools protein lists to Webgestalt were evaluated for statistical significance using the hypergeometric test (Zhang, Kirov, & Snoddy, 2005). To test differences in aggregation propensity across proteomes, data were analysed for normality using the Shapiro-Wilk test. Non-parametric group analysis was performed using Kruskal–Wallis one-way test of variance on ranks with Dunn's multiple comparison as post-test using GraphPad(v7).

To test ALS versus HC proteolytic bands intensity difference, a "t-Test - Two-Sample Assuming Unequal Variances" was performed. Principal component analysis (PCA) was used to study the variance of the data sets generated by the TMTcalibrator™ workflow. To determine the regulated features (FeaST), LIMMA considered the following linear model: logRatio(ALS/HC) ≈ class + group + gender + progression rate + TMT batch. Multiple testing corrections and false discovery rate (FDR) were obtained

For Review Only

using the Benjamini-Hochberg procedure. For the Functional analysis (FAT), a two-sided p-value was generated by the Mann Whitney U test and the Benjamini-Hochberg method was used for multiple test correction. Expression values were normalised with other 1000 randomly selected background expression values. A minimum of three matched identifiers (e.g. gene names) were required for each term. Terms with an adjusted p-value < 0.3 were considered significant. Cell survival assays were evaluated by two-way ANOVA and Tukey HSD test.

Study approval

A written informed consent was obtained from all ALS and HC participants enrolled in the ALS biomarkers study (REC n. 09/H0703/27). Written informed consent was obtained from donors of brain samples for the use of the material and clinical information for research purposes (Netherland Brain Bank: 2009/148).

Results

Study participants

The mass spectrometry (MS)-based proteomic study of CPA was performed on 2 pools of plasma samples (PPS), one containing samples from three fast and three slow progressing ALS individuals and one from six HC individuals (ALS: 5 males (M), 1 female (F); HC: 3M, 3F; age range: ALS: 46.1-78.5; HC 51.8-62.9; Supplementary Table 1).

For the TMT® proteomic experiment, for protease digestion and for validation by western blot, CPA extracted from plasma samples from ALS and HC cohorts (male/female ratio: 3:3; age range: ALS 60.2-68.8; HC: 60.6-68.3; Supplementary Table 2 and 3) were tested individually.

Extraction of circulating (CPA) and brain protein aggregates (BPA): qualitative analysis by transmission electron microscopy

To verify the efficiency of CPA and BPA extraction protocols, we have used transmission electron microscopy (TEM) to visualize the aggregate fractions after UC of plasma samples (3 HC and 3 ALS

For Review Only

cases) and ALS brains. TEM revealed the presence of (macromolecular) amorphous electron-dense particles of different size, in both CPA and BPA (Fig. 1A and B respectively). With the same extraction protocol, in CPA but not in BPA grids, it was possible to appreciate small, round (few nm diameter) particles close or superimposed to the bigger, globular and more electron-dense bodies. As previously reported, these formations may represent micelles composed of lipids, detergents or lipoproteins (Safar et al., 2006; Terry et al., 2016) (Fig. 1 A and B), such as very low-density (VLDL), low-density (LDL) and high-density lipoproteins (HDL). Both lipoproteins and biochemical pathways linked to their metabolism were in fact detected and found significantly regulated in the CPA proteomic study (described below). Unlike the large, amorphous, globular appearance of CPA aggregates, some of the electron-dense formations in the BPA micrographs had filamentous and donut-like shapes (Fig. 1 B and C), suggestive of contamination with ferritin of brain homogenates as previously reported (Quintana, Cowley, & Marhic, 2004; Sana, Poh, & Lim, 2012; Zhou et al., 2009). In CPA grids only, it was possible to see filamentous fragments with a rough surface similar to those seen in BPA (Fig. 1D, E and F).

For Review Only

Figure 1. Micrographs of circulating protein aggregates (CPA) and brain protein aggregates (BPA) taken by transmission electron microscopy.

(A) grid micrograph after CPA sample loading showing an amorphous globular formation with adjacent and/or superimposed smaller rounded particles (which may be formed of lipoproteins). (B) grid micrograph of BPA showing amorphous electron-dense (left-hand side) as well as short filamentous and small round formations. (C) Details of filamentous and of donut-like particles detected in BPA micrographs. (D, E, F) Micrograph grids of CPA showing 13 to 20 nm thick and 70 to 145 nm long fragments. Scale bar on the lower right-hand corner of each micrograph.

Circulating protein aggregates (CPA) and brain protein aggregates (BPA) composition: LC-MS/MS proteomics

Liquid Chromatography coupled with Tandem Mass Spectrometry (LC-MS/MS) after in-gel trypsin digestion was used to study protein aggregates enriched from ALS and HC pooled plasma samples (PPS) as well as from ALS brains.

In total, 367 proteins were identified in ALS CPA and 353 in HC CPA (Fig. 2A). 264 (57.9% of the total) proteins were expressed in both ALS and HC CPA (here defined as shared), while 103 (22.6%) were

found only in ALS (defined as unique ALS) and 89 (19.5%) in HC CPA (defined as unique Controls) (Fig. 2A). Functional analysis of these protein sub-sets was performed using Webgestalt for Kyoto Encyclopaedia of Genes and Genomes (KEGG) pathway enrichment. Among the most enriched pathways, the proteasome was the most significantly represented feature in ALS ($p=0.028$; four genes matched in this category) while the glycolysis/gluconeogenesis pathway ($p=0.009$; seven genes matched), the pentose phosphate pathway ($p=0.003$; five genes matched) and the carbon metabolism ($p=0.008$; eight genes matched) were significantly expressed in HC. Proteins previously linked to ALS like NfH or TDP-43 were not detected.

LC-MS/MS identified 48 protein groups in brain protein aggregates (BPA), including the three neurofilament isoform proteins. There was little overlap between CPA and BPA proteins (Fig. 2B), with only five proteins identified in both types of aggregates, one identified in both ALS CPA and BPA and one in both HC CPA and BPA.

Figure 2. Comparison of circulating protein aggregates (ALS and HC) and brain protein aggregates (ALS) composition.

(A) Venn diagram showing CPA proteins unique to or shared by ALS and HC. (B) Venn diagram showing HC and ALS CPA proteins shared by brain aggregates. Five proteins were expressed in all 3 aggregate groups (actin cytoplasmic 1, tubulin alpha-4A chain isoform 2, clathrin heavy chain 1 isoform 2, collagen alpha-1(VI) and plectin isoform 7), while brain aggregates shared only one protein with ALS and HC CPA (cytoplasmic dynein 1 heavy chain 1 and collagen alpha-2(VI), respectively).

Aggregation propensity

The brain and plasma aggregate protein lists generated by LC-MS/MS were studied to test the propensity to aggregation in each dataset. The distribution of protein size or molecular weight (MW), isoelectric point (pI) and hydrophobicity, expressed as GRAVY index, were analysed in each proteome dataset with the whole human proteome as reference (Weids, Ibstedt, Tamas, & Grant, 2016). BPA had a significantly higher MW ($p<0.0001$) compared to the other datasets (ALS, HC, shared respectively and Human proteome, Fig. 3A), while pI was significantly lower in all CPA datasets compared to the Human proteome ($p<0.0001$; Fig. 3B). Despite minimal overlap in protein composition between CPA and BPA

For Review Only

(Fig. 2B), aggregation propensity in the two aggregate types and in the human proteome was similar when measured by GRAVY index, which takes into account the average hydropathy of a peptide according to its aminoacidic composition (Fig. 3C).

Figure 3. Aggregation propensity of proteins in blood and brain aggregates from ALS and HC compared to the Human proteome.

Molecular weight (MW) (A), isoelectric point (pI) (B) and hydrophobicity (GRAVY index) (C), known to affect aggregation propensity, are compared across proteins found to be expressed only in ALS and HC CPA (ALS and HC respectively), proteins shared between ALS and HC CPA datasets (Shared), proteins within brain aggregates (Brain) and in the entire human proteome. The distribution plots show the dispersion of the samples with relative frequency, while the violin plots show median and interquartile ranges. Statistical analysis was performed using one-way ANOVA, Kruskal-Wallis test with Dunn's multiple comparison as post-test for group analysis with * expressing the level of significance (*: $p = 0.0251$; ****: $p < 0.0001$).

For Review Only

CPA protease digestion and NfH resistance

Resistance to protease digestion has been described as a key feature of altered protein behavior in conditions like prion disease (McKinley, Bolton, & Prusiner, 1983). We have previously shown that neurofilament heavy chain (NfH) is found in blood CPA (Adiutori et al., 2018; Lu, Kalmar, Malaspina, Greensmith, & Petzold, 2011). As NfH is constitutively expressed in protein aggregates from ALS brain and has been linked to the pathogenesis of the disease, we looked at NfH protease resistance in CPA from ALS and compared its digestion profile to that in HC CPA (Fig. 4). CPA enriched from the ALS and HC plasma samples were treated with trypsin, chymotrypsin, enterokinase and calpain. Both CPA NfH digested and undigested profiles were analysed.

Western blot analysis of NfH in plasma CPA before digestion detected three bands at 460, 268 and 41 KDa as previously reported (Fig. 4A) (Adiutori et al., 2018). The sum of all NfH band intensities (SUM) was (not significantly) higher in the ALS group compared to HC. The ratio between the intensity of the 460 KDa band and the NfH SUM intensity (460/SUM) was higher in HC, while the ratio between the intensities of the 268 and 460 bands (268/460) was significant higher in ALS (Fig. 4A).

Treatment with trypsin or chymotrypsin showed an almost complete digestion of NfH in both ALS and HC, with the exception of a residual 41 KDa band present in a minority of samples (data not shown). After calpain digestion, there was a different pattern of immunoreactivity for each CPA sample with the exception of 58 and 41 KDa bands evenly detected in all samples (Fig. 4B). Enterokinase digestion resulted in a 49 KDa band in all samples with equal expression in ALS and HC (Fig. 4C). All ALS samples showed bands at 171 and 31 KDa not seen in HC samples (Fig. 4C).

NfH in BPA showed low or no resistance to digestion with all three enzymes (Fig. 4D). Chymotrypsin and enterokinase digestions (Fig. 4D, lane 2 and 3 respectively) generated no distinct bands but a faint smear at higher molecular weight than the NfH bands detected in undigested brain and brain lysate (Fig. 4D, lane 1 and 5 respectively). Calpain digestion showed two faint bands at about 171 KDa (Fig. 4B, lane 4).

For Review Only

Figure 4. Western blot analysis of neurofilament heavy chain (NfH) within circulating protein aggregates (CPA) after proteases digestion.

Undigested CPA (A) show NfH bands at 460, 268 and 41 KDa (268 KDa is NfH expected molecular weight). The ratio between the 460 KDa band and the the sum of all NfH band intensities (SUM, 460/SUM) is higher in HC (p= 0.032), while the ratio between the 268 and 460 bands (268/460) is higher in ALS (p= 0.018). Calpain digestion (B) shows 58 and 41 KDa bands in all samples with no difference in expression. The enterokinase digestion profile of NfH (C) shows a 49 KDa band uniformly expressed across samples and additional 171 and 31 KDa bands only in ALS patients (blue arrows). Undigested NfH in ALS brain protein aggregates (BPA; D, lane 1) and after digestion with chymotrypsin (lane 2), enterokinase (lane 3), calpain (lane 4) and brain lysate lane 5. To maximise band visualization, time exposure was for lane 1 at 10.1 seconds, lane 4 and 5 at 58.4 seconds and lane 2 and 3 at 278.8 seconds. In D, the lanes have been rearranged to simplify comparison with CPA data shown in A, B and C. The original blot is included in the Supplementary Material.

TMTcalibrator™: brain-derived proteins in CPA

The observed lack of similarity in the composition of brain and plasma aggregates may relate to the limits of proteomic techniques, whereby low abundance (brain-derived) proteins, may be masked by those with high abundance and detection confounded by the presence of post-translational modifications. To address these shortfalls and gather more information on the potential enrichment of brain-derived proteins in circulating aggregates, we have undertaken further proteomics using a TMTcalibrator™ workflow, where brain lysate was used to enhance detection of proteins in CPA (Leoni et al., 2019; Russell et al., 2016; Zubiri et al., 2018).

4973 brain-derived proteins were identified, including the three neurofilaments (Nf) protein isoforms (Nf Light (NfL), Nf Medium (NfM) and Nf Heavy (NfH). Nf were found at a relatively higher level in ALS compare to HC samples (log2-fold change ALS/HC (logFC) = 0.093, 0.181 and 0.298, respectively) but none was significantly regulated (p= 0.40, 0.16 and 0.06 respectively). Of the 4973 brain-derived proteins, 285 proteins (5.7%) showed a statistically significant regulation ($p < 0.05$). 158 were more expressed in HC (logFC < 0) with an average fold-change (FC) of -0.667, while 127 in ALS (logFC > 0) with an average FC of 0.703. The protein list obtained was matched with an ALS gene list obtained from

For Review Only

the MalaCards database, an integrated repository of human diseases and their annotations. 24 ALS proteins were identified including Fused in Sarcoma RNA-binding protein (FUS) which was found to be significantly regulated in ALS (p= 0.00696) (Table 1).

Principal component analysis (PCA) of the proteomic data after correction for the TMT® batch-effect identified the sample origin (ALS or HC) as the strongest component (41.17%) of the total variance in the data matrix (Fig. 5A). The marked separation between ALS and HC in CPA brain-derived proteins identified by PCA was also observed when using clustering of these regulated proteins (Fig. 5B). Considering the CPA enrichment of brain proteins (Fig. 5C), we speculate that circulating protein assemblies represent a good source of biomarkers for ALS, which may be difficult to detect in whole plasma analysis..

For Review Only

Table 1. ALS risk genes included in the list of proteins identified using the TMTcalibrator™ workflow in CPA from ALS and HC and reported in the gene classifiers MalaCards Human Disease Database (https://www.malacards.org/card/amyotrophic_lateral_sclerosis_1#RelatedGenes-table) (Database, n.d.). Among a total of 38 ALS elite genes reported in the MalaCards Human Disease Database (those more likely to cause the disease), 24 were detected in the list of proteins generated by the TMTcalibrator™ experiment.

Figure 5. TMTcalibrator™ proteomic analysis.

(A) Principal component analysis (PCA) showing a separation between the ALS and HC experimental groups regulated features at protein level. Dimension 1 or the variance between the two experimental groups (ALS and HC) is 41.17% of the entire variance; dimension 2 or variance between 10plexes (TMT01 and TMT02) is 9.45% of the entire variance. (B) Heatmap showing the distribution of the regulated features and their clustering. The regulated features are distributed vertically, reported as Uniprot IDs on the right-hand side and relative clustering on the left-hand side. Analytical samples are distributed horizontally, with sample names at the bottom and relative clustering at the top of the heatmaps. The color key histogram at the top left side shows the distribution of the features and the heatmap color coding. (C) The volcano plot shows the distribution of the proteins identified by TMT proteomic study according to their fold change (FC) expressed as log2 (fold change ALS/HC) (logFC)

in the x axis and according to p-value expressed as –log10 (p-value) in the y axis. Protein groups were considered regulated if p-value < 0.05 and logFC < -0.58 or > 0.58. Red dots are regulated features, yellow dots are features with a significant p-value ($p < 0.05$) and logFC between -0.58 and 0.58 while green dots are not regulated protein groups ($p > 0,05$). Uniprot IDs are reported beside the dots with significant p-value.

Functional analysis

We have assessed the relevance of different biological terms in the proteomic data, based on their over-representation in the subset of regulated proteins (Leoni et al., 2019; Zubiri et al., 2018). Within the top ten regulated biochemical pathways of the 69 identified ($p < 0.05$), five were involved in metabolism of lipoproteins (Supplementary Table 7). Several authors already described that metabolism in ALS is switched from sugars and carbohydrates to lipids use (Delaye et al., 2017; Szelechowski et al., 2018; Tefera & Borges, 2017). Within the 69 pathways highlighted by FAT, we have looked at highly regulated protein (unique peptides ≥ 2, LogFC < -0.693 or > 0.693, p-value < 0.05) to evaluate other pathways potentially involved with aggregation and neurodegeneration, but also to obtain potential targets for patient stratification. In this way we identified 48 proteins (Supplementary Table 8) from which we could highlight four biochemical pathways: metabolism of carbohydrates (p= 0.0099), glycosaminoglycans (GAGs) metabolism, lysosome (p= 0.0015), synthesis of phosphatidic acid (PA; p= 0.0184) and wnt signalling pathway (p= 0.0337). GAGs are involved in protein aggregation and prion diffusion (Ancsin, 2003; DeWitt, Richey, Praprotnik, Silver, & Perry, 1994; Forostyak et al., 2014; Foyez et al., 2015; Hirano et al., 2013; Holmes et al., 2013; Nishitsuji, 2018; Sarrazin, Lamanna, & Esko, 2011; Shijo et al., 2018). Lysosome activity changes have been described in ALS caused by the C9orf72 gene repeat expansions (Cipolat Mis, Brajkovic, Frattini, Di Fonzo, & Corti, 2016; Sasaki, 2011; Shi et al., 2018; Song, Guo, Liu, & Tang, 2012; Sullivan et al., 2016), while synthesis of PA and related phospholipids, including phosphatidylcholine and phosphatidylethanolamine, have been linked to ALS and prion disease pathogenesis (Blasco et al., 2017; Supattapone, 2012).

Proteomic results validation by immunoassays

Six proteins belonging to ALS-relevant molecular pathways were tested by western blot: Glypican-4 (GPC4), Fibromodulin (FMOD), Biglycan (BGN), Cation-dependent mannose-6-phosphate receptor

For Review Only

(M6PR), Endophilin-B2 (SH3GLB2) and Protein DJ-1 (PARK7) using CPA extracted from ALS patients and HC, with brain lysate as reference. SH3GLB2 showed the same trend of ALS vs HC protein regulation identified in the TMTcalibrator™ experiment, with a similar level of regulation (logFC= 0.34 in TMTcalibrator™ and log2(ALS/HC)= 0.437) in western blot analysis (Fig. 6). The remaining proteins showed a different trend of regulation compared to that obtained in the proteomic analysis (data not shown). SH3GLB2 and M6PR were detected at a higher MW in CPA compared to brain lysate, possibly in keeping with a different PTM profile in tissues as opposed to fluids which may affect the state of aggregation and protease digestion.

Figure 6. Western blot analysis of Endophilin-B2 (SH3GLB2) in plasma CPA from ALS patients and healthy controls.
Samples were normalized to HC6 density and the average values with relative standard deviation for the ALS (n=4) and Control (n=4) groups were plotted onto the chart. A brain lysate sample is also included (1st lane, red band, indicating signal saturation) which showed an endophilin-B2 band at a lower molecular weight than the bands detected in CPA. Immunodetection confirmed the SH3GLB2 higher level of expression in the ALS CPA compared to control (logFC= 0.34), but without being statistically significant (p= 0.57).

For Review Only

Aggregation propensity of the brain-derived proteins in CPAs

We evaluated aggregation propensity of the CPA proteins identified by TMTcalibrator™ compared to the Human proteome and BPA, using the reported physicochemical parameters. The analysis was performed on the entire TMT® proteome dataset and on the 285 regulated proteins only, which were divided in two groups based on the level of differential expression: log(ALS/HC)>0 and log(ALS/HC)<0. There was no statistically significant difference among these three datasets for the parameters under investigation. However, the entire TMT and BPA proteome datasets showed statistically significant higher MW and lower pI compared to the Human proteome (P <0.0001), while for GRAVY index, the BPA values were lower than TMT and Human proteome (p= 0.0348 and 0.0224, respectively; data not shown).

Cell survival assays

To test the biological effects of CPA on living cells, human brain microvascular endothelial cells (hCMEC/D3) modelling human blood-brain barrier (BBB) and PC12 neuron-like cells lines were treated with CPA from ALS patients and HC and cell viability measured (Fig. 7). Total IgG were extracted from the same blood samples CPA were separated from and used to treat the same cell lines. CPA re-suspended in PBS were administered at defined concentrations to hCMEC/D3, while CPA were pre-treated with 8M urea before testing PC12 viability. Dissolution of aggregates by urea for PC12 cells was undertaken to evaluate the effect of CPA-containing proteins rather than the effect of their aggregated state. PC12 cell viability decreased at increasing concentration of CPA, (to 75% at 0.5 µg/ml; Fig. 7B), while endothelial cells showed the opposite trend, with the maximum effect on cell viability at the lowest concentration (0.05 µg/ml) and no effect at the highest concentration (1 µg/ml; Fig. 7A). ALS CPA exerted higher toxicity (lower cell viability) at lower concentration (0.05 µg/ml) with endothelial cells and with PC12 cells (0.5 µg/ml) compared to HC CPA (Fig. 7). IgG extracted from the same ALS and HC plasma samples as CPA, showed reduction of endothelial cell viability to 80% and to between 70 and 85% in PC12 cells at 1.5 µg/ml, with no significant difference between ALS and HC (Fig. 7).

Figure 7. Cell viability after treatment with aggregates, solubilized aggregates and immunoglobulins extracted from plasma samples.

The figure shows the percentage of endothelial (hCMEC/D3) and PC12 living cells (A and B) after treatment with different concentrations of CPA and IgG from ALS and HC. Cells treated with ALS CPA showed a statistically significant lower cell viability compared to HC CPA treated cells at 0,05 µg/ml ($p= 0.031$; endothelial cells, A) and at 0,1 µg/ml ($p= 0.029$; PC12 cells, B). IgG had minor effect on all cell type viability with no difference between ALS and HC. CPA proteins were solubilized with 8M urea before PC12 cells treatment. Significance was tested by two-way ANOVA and Tukey HSD test.

Discussion

In this paper we show that in ALS, an incurable neurodegenerative disorder, circulating protein aggregates (CPA) are enriched with proteins implicated in the proteasome system, an essential clearance mechanism of defective proteins (Ling, Polymenidou, & Cleveland, 2013; Saez & Vilchez, 2014). Using a TMTcalibrator™ proteomic approach we also show, for the first time, that these aggregates contain

For Review Only

just below 5,000 brain peptides, including a significant number of proteins linked to ALS risk genes such as FUS and SOD1 (Table 1). Chemical features defining propensity to aggregate of CPA proteins differ from those seen in brain aggregates and from the whole human proteome. Plasma CPA and particularly low concentration ALS CPA, affect both endothelial and neuronal cells viability, showing a more pronounced biological effect than that observed using the immunoglobulin fraction extracted from the same plasma samples (Fig. 7).

The TMTcalibrator™ workflow employed in this study has the added value of being able to analyze tissues and fluids in the same experiment, enhancing the detection of low-abundance fluid proteins that are also expressed in brain, most of them undetectable using standard proteomics (Adiutori et al., 2018). Of these brain proteins, 285 show a significant level of regulation ($p < 0.05$) in ALS compared to controls (Fig. 5). The detection by proteomics of these low-abundance brain proteins in CPA has important methodological implications. For the identification of the same protein targets, immunoassays may suffer from competition of naturally occurring autoantibodies causing epitope sequestration in aggregates and immunocomplexes as recently shown for neurofilaments (C. H. Lu et al., 2011; C. H. Lu, Petzold, et al., 2015). Unlike TMTcalibrator™ enhanced detection based on an internal tissue calibrant, standard MS-based proteomics would, in turn, lack the sensitivity to discriminate low concentration against more abundant proteins. The mismatch between MS and antibody-based peptide recognition is clearly shown in our validation experiment by western blot, where the use of an orthogonal technique of immunodetection does not always confirm the findings on proteomics. This is not surprising considering that these methods may target different peptides of the same protein producing results that may not be congruent. Despite the inherent limitations of our approach, this study provides a novel strategy for biomarker discovery and measurement in ALS, based on the analysis of proteins compartmentalized in aggregates.

The method of aggregates separation employed in our study does not allow the analysis by proteomics of the supernatant from processed samples, due to detergent contamination following CPA extraction. To circumvent this problem, we have previously used low complexity binders for aggregate separation in biofluid, only to observe that the resulting aggregate fraction had a substantially different protein composition to the one obtained by ultracentrifugation, the method of choice of the current study (Adiutori et al., 2018). It is therefore not possible to compare the protein profile of the aggregates and fluid components of the same plasma sample. To circumvent this problem, we have recently used two

For Review Only

separate proteomic workflows, including brain-enhanced TMT proteomics, to study the immunological response and the plasma/brain proteome in phenotypic variants of ALS (Leoni et al., 2019). In the whole plasma, we have identified only nine ALS elite genes out of the 24 identified in our CPA study (including Profilin-1 and the Isoform 2 of Heterogeneous nuclear ribonucleoprotein A1). We may therefore speculate that aggregates in blood are more enriched with neuron-derived and disease-specific proteins compared to the fluid component of plasma, making them a more desirable target in the search for ALS biomarkers.

When compared to a healthy state, functional analysis of the plasma CPA proteome from ALS individuals reveals pathological hallmarks of ALS, including changes in the proteasome-dependent protein degradation and in energy metabolism pathways (Ling et al., 2013; Ngo & Steyn, 2015; Palamiuc et al., 2015; Saez & Vilchez, 2014). The TMTcalibrator™ dataset contains regulated features also known to be involved in ALS pathology including lysosome as well as lipoprotein and glycosaminoglycan metabolisms (Ling et al., 2013; Ngo & Steyn, 2015; Palamiuc et al., 2015; Saez & Vilchez, 2014; Sasaki, 2011; Shi et al., 2018; Sullivan et al., 2016; Szelechowski et al., 2018; Tefera & Borges, 2017) (Supplementary Table 7). To our knowledge, changes in proteasome activity in ALS have so far been shown in such detail only in brain, spinal cord and in neuronal cell lines, but not systemically or more specifically in blood (Ling et al., 2013; Saez & Vilchez, 2014).

The analysis of the physicochemical properties that facilitate proteins aggregation in our protein datasets provides further insight into protein behavior in different molecular environments and in relation with a disease state like ALS. When molecular weight and isoelectric point are taken into account, we observe that the tissue or fluid of origin are the main contributors to the differences in the proteome chemical properties observed across the aggregate types and not the presence or absence of ALS (Fig. 3). When the whole human proteome is included in the analyses as reference, it is possible to see how proteins in the aggregated state are distinguishable from the whole set of human proteins, regardless of their tissue or fluid of origin or presence or absence of a pathological state like ALS. With regard to hydrolytic properties, we have identified clear differences after enterokinase digestion between ALS and HC CPA, with the presence of specific NfH digestion fragments at 171 KDa and at 31 KDa only in ALS samples (Fig. 4C). While a trend for NfH proteolytic fragments over-expression in ALS CPA compared to HC is visible in our experimental data, our study is not powered with enough samples to establish this observation along what already previously reported (C.-H. Lu et al., 2012; C. H. Lu, Petzold, et al., 2015).

For Review Only

An extension of this preliminary finding using a larger number of samples will be needed to compare our observation to previously reported data on the same neurofilament isoform enterokinase digestion pattern in a different experimental context (Petzold et al., 2011).

We have tested different cell lines using change in cell viability upon treatment with ALS and HC CPA as a readout of the biological effects of circulating aggregates (Fig. 7). Given that immunoglobulins make up a sizeable share of plasma proteins and that plasma has been shown to have a systemic biological effect (Lehallier et al., 2019; Williams et al., 2019), we have treated the same cell lines with immunoglobulins extracted from the blood samples CPA were recovered from. Whilst immunoglobulins seem not to significantly interfere with cell function at different concentration, there is a detectable toxic effect on PC12 neurons and on hCMEC/D3 endothelial cell cultures upon treatment with CPA, and a clear ALS-specific change in cell viability when CPA are administered at relatively low concentrations (Fig. 7). CPA may well be the proteinaceous component in blood that is ultimately responsible for the BBB damage that has been reported in ALS (Garbuzova-Davis et al., 2012). It is not possible to explain how endothelial cell damage comes into play upon exposure to CPA, and whether the ALS-specific effect relates to a particular composition and/or conformation of aggregates which may be concentration-dependent. The use of TEM to confirm the efficiency of aggregates separation confirms the presence of particles of both globular and filamentous appearance, similar to those observed in BPA (Fig. 1). Further investigation by TEM may be needed to evaluate whether the ALS-specific CPA effect on cells relate to a particular conformation, size or composition of aggregates.

For Review Only

To date, there is no evidence in the literature of investigations into non-membrane bound particles in blood stream of ALS patients and their potential use in biomarkers discovery. Our data indicate that circulating protein aggregates represent a new source of biomarkers enriched with brain proteins, including disease-relevant proteins, that can become regulated under pathological condition. These aggregates appear biologically active as they affect endothelial and neuronal cell viability when administered to cell culture. Further investigation on the nature of these particles will be required to confirm and strengthen this initial finding, including a more extensive comparison with brain aggregates and analysis of the biochemical characteristics in a larger subset of individuals, using more effective and user-friendly methods of CPA extraction.

Acknowledgments

We would like to thank the patients and their families along with all healthy donors for their contribution. This study is funded by a Medical Research Council (MRC) Industry CASE Studentship (grant number: MR/M015882/1) awarded to Queen Mary University of London, in collaboration with Proteome Sciences. Plasma samples were obtained from the ALS biomarkers study (09/H0703/27). Support for the development of the methodology outlined in this paper has also come from EU2020 funding (H2020 PHC-13-2014): "Efficacy and safety of low-dose IL-2 (ld-IL-2) as a Treg enhancer for anti-neuroinflammatory therapy in newly diagnosed Amyotrophic Lateral Sclerosis (ALS) patients" (MIROCALS)". The mass spectrometry proteomics data have been deposited to the ProteomeXchange Consortium via the PRIDE (Perez-Riverol et al., 2019) partner repository with the dataset identifier PXD018938 and PXD018923.

For Review Only

Funding

This project was funded by the Medical Research Council (MRC) with an Industry CASE Studentship, the Motor Neuron Disease Association (MNDA) UK for the ALS Biomarkers study and the EU2020 for MIROCALS.

Competing interests

The authors declare that they have no competing interests.

Supplementary Material

Attached to submission.

References

Adiutori, R., Aarum, J., Zubiri, I., Bremang, M., Jung, S., Sheer, D., … Malaspina, A. (2018). The proteome of neurofilament-containing protein aggregates in blood. *Biochemistry and Biophysics Reports*, *14*, 168–177. https://doi.org/10.1016/j.bbrep.2018.04.010

Ancsin, J. B. (2003). Amyloidogenesis: Historical and modern observations point to heparan sulfate

proteoglycans as a major culprit. *Amyloid*, *10*(2), 67–79. https://doi.org/10.3109/13506120309041728

Blasco, H., Veyrat-Durebex, C., Bocca, C., Patin, F., Vourc'H, P., Kouassi Nzoughet, J., … Reynier, P. (2017). Lipidomics Reveals Cerebrospinal-Fluid Signatures of ALS. *Scientific Reports*. https://doi.org/10.1038/s41598-017-17389-9

Cipolat Mis, M. S., Brajkovic, S., Frattini, E., Di Fonzo, A., & Corti, S. (2016). Autophagy in motor neuron disease: Key pathogenetic mechanisms and therapeutic targets. *Molecular and Cellular Neuroscience*, *72*, 84–90. https://doi.org/10.1016/J.MCN.2016.01.012

Database, M. T. human disease. (n.d.). ALS elite genes. Retrieved from https://www.malacards.org/card/amyotrophic_lateral_sclerosis_1#RelatedGenes-table

Delaye, J. B., Patin, F., Piver, E., Bruno, C., Vasse, M., Vourc'h, P., … Blasco, H. (2017). Low IDL-B and high LDL-1 subfraction levels in serum of ALS patients. *Journal of the Neurological Sciences*. https://doi.org/10.1016/j.jns.2017.07.019

DeWitt, D. A., Richey, P. L., Praprotnik, D., Silver, J., & Perry, G. (1994). Chondroitin sulfate proteoglycans are a common component of neuronal inclusions and astrocytic reaction in neurodegenerative diseases. *Brain Research*, *656*(1), 205–209. https://doi.org/10.1016/0006-8993(94)91386-2

Forostyak, S., Homola, A., Turnovcova, K., Svitil, P., Jendelova, P., & Sykova, E. (2014). Intrathecal delivery of mesenchymal stromal cells protects the structure of altered perineuronal nets in SOD1 rats and amends the course of ALS. *Stem Cells*. https://doi.org/10.1002/stem.1812

Foyez, T., Takeda-Uchimura, Y., Ishigaki, S., Narentuya, N., Zhang, Z., Sobue, G., … Uchimura, K. (2015). Microglial keratan sulfate epitope elicits in central nervous tissues of transgenic model mice and patients with amyotrophic lateral sclerosis. *American Journal of Pathology*, *185*(11), 3053–3065. https://doi.org/10.1016/j.ajpath.2015.07.016

Hirano, K., Ohgomori, T., Kobayashi, K., Tanaka, F., Matsumoto, T., Natori, T., … Kadomatsu, K. (2013). Ablation of Keratan Sulfate Accelerates Early Phase Pathogenesis of ALS. *PLoS ONE*.

For Review Only

https://doi.org/10.1371/journal.pone.0066969

Holmes, B. B., DeVos, S. L., Kfoury, N., Li, M., Jacks, R., Yanamandra, K., … Diamond, M. I. (2013). Heparan sulfate proteoglycans mediate internalization and propagation of specific proteopathic seeds. *Proceedings of the National Academy of Sciences*. https://doi.org/10.1073/pnas.1301440110

Hoyles, L., Snelling, T., Umlai, U.-K., Nicholson, J. K., Carding, S. R., Glen, R. C., & McArthur, S. (2018). Microbiome–host systems interactions: protective effects of propionate upon the blood–brain barrier. *Microbiome 2018 6:1*, *6*(1), 55. https://doi.org/10.1186/s40168-018-0439-y

Leoni, E., Bremang, M., Mitra, V., Zubiri, I., Jung, S., Lu, C.-H., … Malaspina, A. (2019). Combined Tissue-Fluid Proteomics to Unravel Phenotypic Variability in Amyotrophic Lateral Sclerosis. *Scientific Reports*, *9*(1). https://doi.org/10.1038/s41598-019-40632-4

Lu, C. H., Kalmar, B., Malaspina, A., Greensmith, L., & Petzold, A. (2011). A method to solubilise protein aggregates for immunoassay quantification which overcomes the neurofilament "hook" effect. *J Neurosci Methods*, *195*(2), 143–150. https://doi.org/10.1016/j.jneumeth.2010.11.026

Ludolph, A., Drory, V., Hardiman, O., Nakano, I., Ravits, J., Robberecht, W., & Shefner, J. (2015). A revision of the El Escorial criteria - 2015. *Amyotrophic Lateral Sclerosis & Frontotemporal Degeneration*, Vol. 16, pp. 291–292. https://doi.org/10.3109/21678421.2015.1049183

McKinley, M. P., Bolton, D. C., & Prusiner, S. B. (1983). A protease-resistant protein is a structural component of the scrapie prion. *Cell*, *35*(1), 57–62.

Nishitsuji, K. (2018). Heparan sulfate S-domains and extracellular sulfatases (Sulfs): their possible roles in protein aggregation diseases. *Glycoconjugate Journal*, 387–396. https://doi.org/10.1007/s10719-018-9833-8

Perez-Riverol, Y., Csordas, A., Bai, J., Bernal-Llinares, M., Hewapathirana, S., Kundu, D. J., … Vizcaíno, J. A. (2019). The PRIDE database and related tools and resources in 2019: Improving support for quantification data. *Nucleic Acids Research*. https://doi.org/10.1093/nar/gky1106

For Review Only

Quintana, C., Cowley, J. M., & Marhic, C. (2004). Electron nanodiffraction and high-resolution electron microscopy studies of the structure and composition of physiological and pathological ferritin. *Journal of Structural Biology*. https://doi.org/10.1016/j.jsb.2004.03.001

Russell, C. L., Mitra, V., Hansson, K., Blennow, K., Gobom, J., Zetterberg, H., … Pike, I. (2016). Comprehensive Quantitative Profiling of Tau and Phosphorylated Tau Peptides in Cerebrospinal Fluid by Mass Spectrometry Provides New Biomarker Candidates. *Journal of Alzheimer's Disease*, *55*(1), 303–313. https://doi.org/10.3233/JAD-160633

Safar, J. G., Wille, H., Geschwind, M. D., Deering, C., Latawiec, D., Serban, A., … Prusiner, S. B. (2006). Human prions and plasma lipoproteins. *Proceedings of the National Academy of Sciences*. https://doi.org/10.1073/pnas.0604021103

Sana, B., Poh, C. L., & Lim, S. (2012). A manganese-ferritin nanocomposite as an ultrasensitive T2contrast agent. *Chemical Communications*. https://doi.org/10.1039/c1cc15189d

Sarrazin, S., Lamanna, W. C., & Esko, J. D. (2011). Heparan sulfate proteoglycans. *Cold Spring Harbor Perspectives in Biology*, *3*(7), 1–33. https://doi.org/10.1101/cshperspect.a004952

Sasaki, S. (2011). Autophagy in spinal cord motor neurons in sporadic amyotrophic lateral sclerosis. *J Neuropathol Exp Neurol*, *70*. https://doi.org/10.1097/NEN.0b013e3182160690

Shi, Y., Lin, S., Staats, K. A., Li, Y., Chang, W. H., Hung, S. T., … Ichida, J. K. (2018). Haploinsufficiency leads to neurodegeneration in C9ORF72 ALS/FTD human induced motor neurons. *Nat Med*. https://doi.org/10.1038/nm.4490

Shijo, T., Warita, H., Suzuki, N., Kitajima, Y., Ikeda, K., Akiyama, T., … Aoki, M. (2018). Aberrant astrocytic expression of chondroitin sulfate proteoglycan receptors in a rat model of amyotrophic lateral sclerosis. *Journal of Neuroscience Research*, *96*(2), 222–233. https://doi.org/10.1002/jnr.24127

Song, C., Guo, J., Liu, Y., & Tang, B. (2012). Autophagy and Its Comprehensive Impact on ALS. *International Journal of Neuroscience*, *122*(12), 695–703. https://doi.org/10.3109/00207454.2012.714430

For Review Only

Sullivan, P. M., Zhou, X., Robins, A. M., Paushter, D. H., Kim, D., Smolka, M. B., & Hu, F. (2016). The ALS/FTLD associated protein C9orf72 associates with SMCR8 and WDR41 to regulate the autophagy-lysosome pathway. *Acta Neuropathologica Communications*. https://doi.org/10.1186/s40478-016-0324-5

Supattapone, S. (2012). Phosphatidylethanolamine as a prion cofactor: Potential implications for disease pathogenesis. *Prion*. https://doi.org/10.4161/pri.21826

Szelechowski, M., Amoedo, N., Obre, E., Léger, C., Allard, L., Bonneu, M., … Rossignol, R. (2018). Metabolic Reprogramming in Amyotrophic Lateral Sclerosis. *Scientific Reports*. https://doi.org/10.1038/s41598-018-22318-5

Tefera, T. W., & Borges, K. (2017). Metabolic dysfunctions in amyotrophic lateral sclerosis pathogenesis and potential metabolic treatments. *Frontiers in Neuroscience*. https://doi.org/10.3389/fnins.2016.00611

Terry, C., Wenborn, A., Gros, N., Sells, J., Joiner, S., Hosszu, L. L. P., … Wadsworth, J. D. F. (2016). Ex vivo mammalian prions are formed of paired double helical prion protein fibrils. *Open Biology*. https://doi.org/10.1098/rsob.160035

Weids, A. J., Ibstedt, S., Tamas, M. J., & Grant, C. M. (2016). Distinct stress conditions result in aggregation of proteins with similar properties. *Sci Rep*, *6*, 24554. https://doi.org/10.1038/srep24554

Weksler, B. B., Subileau, E. A., Perrière, N., Charneau, P., Holloway, K., Leveque, M., … Couraud, P. O. (2005). Blood-brain barrier-specific properties of a human adult brain endothelial cell line. *FASEB Journal : Official Publication of the Federation of American Societies for Experimental Biology*, *19*(13), 1872–1874. https://doi.org/10.1096/fj.04-3458fje

Zhang, B., Kirov, S., & Snoddy, J. (2005). WebGestalt: An integrated system for exploring gene sets in various biological contexts. *Nucleic Acids Research*, *33*(SUPPL. 2), 741–748. https://doi.org/10.1093/nar/gki475

Zhou, Z., Fan, J.-B., Zhu, H.-L., Shewmaker, F., Yan, X., Chen, X., … Liang, Y. (2009). Crowded

For Review Only

Cell-like Environment Accelerates the Nucleation Step of Amyloidogenic Protein Misfolding. *Journal of Biological Chemistry*, *284*(44), 30148–30158. https://doi.org/10.1074/jbc.M109.002832

Zubiri, I., Lombardi, V., Bremang, M., Mitra, V., Nardo, G., Adiutori, R., … Malaspina, A. (2018). Tissue-enhanced plasma proteomic analysis for disease stratification in amyotrophic lateral sclerosis. *Molecular Neurodegeneration*, *13*(1), 60. https://doi.org/10.1186/s13024-018-0292-2

For Review Only

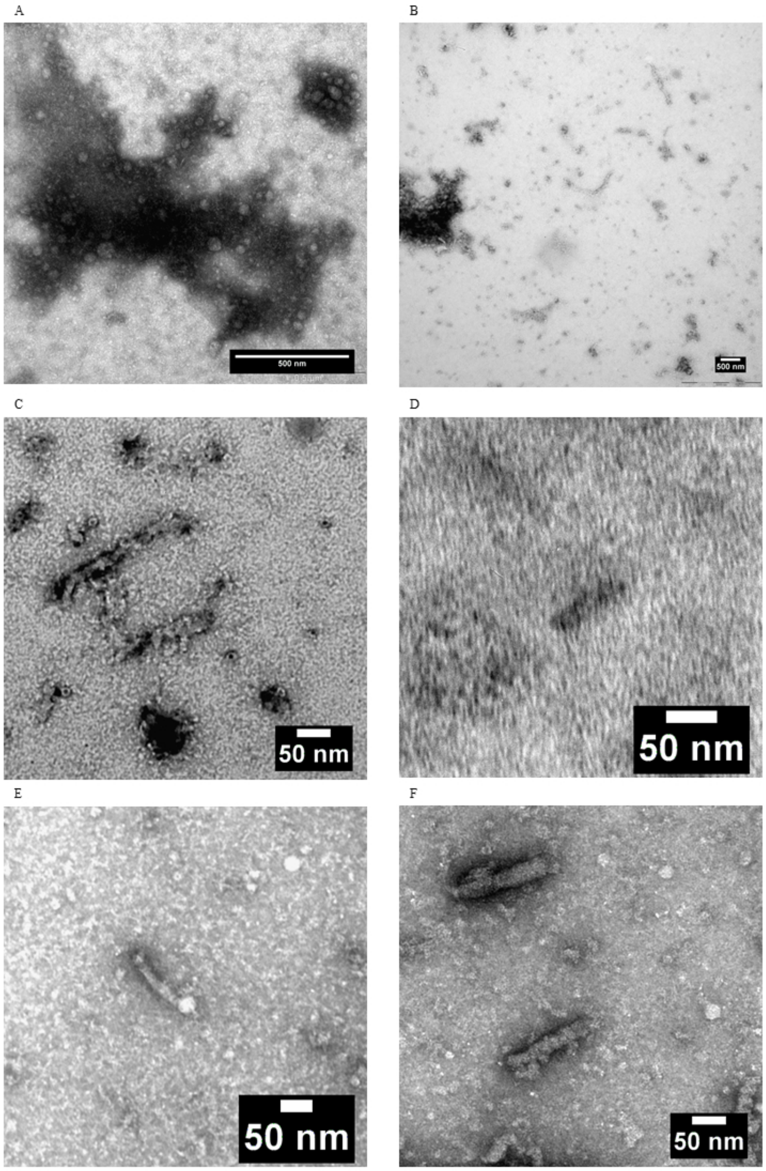

Figure 1. Micrographs of circulating protein aggregates (CPA) and brain protein aggregates (BPA) taken by transmission electron microscopy (TEM) after uranyl acetate (UA) negative staining.
(A) grid micrograph after CPA sample loading showing an amorphous globular formation with adjacent and/or superimposed smaller rounded particles (which may be formed of lipoproteins). (B) grid micrograph of BPA showing amorphous electron-dense (left-hand side) as well as short filamentous and small round formations. (C) Details of filamentous and of donut-like particles detected in BPA micrographs. (D, E, F) Micrograph grids of CPA showing 13 to 20 nm thick and 70 to 145 nm long fragments. Scale bar on the lower right-hand corner of each micrograph.

210x297mm (96 x 96 DPI)

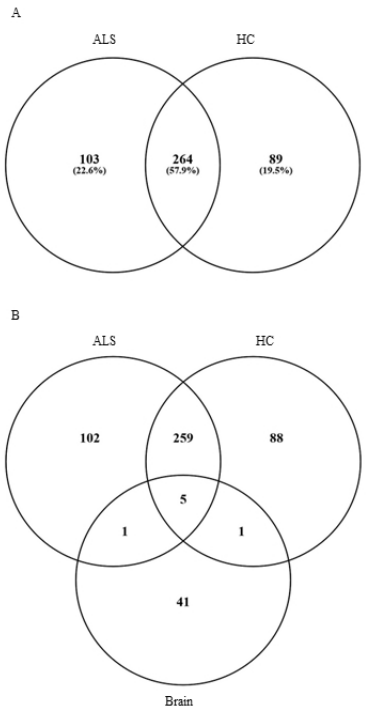

Figure 2. Proteins identified by LC-MS/MS in circulating protein aggregates (CPA) enriched from ALS and HC pooled plasma samples and in aggregates enriched from brain.
(A) Venn diagram showing CPA proteins unique to or shared by ALS and HC. (B) Venn diagram showing HC and ALS CPA proteins shared by brain aggregates. Five proteins were expressed in all 3 aggregate groups (actin cytoplasmic 1, tubulin alpha-4A chain isoform 2, clathrin heavy chain 1 isoform 2, collagen alpha-1(VI) and plectin isoform 7), while brain aggregates shared only one protein with ALS and HC CPA (cytoplasmic dynein 1 heavy chain 1 and collagen alpha-2(VI), respectively).

210x297mm (96 x 96 DPI)

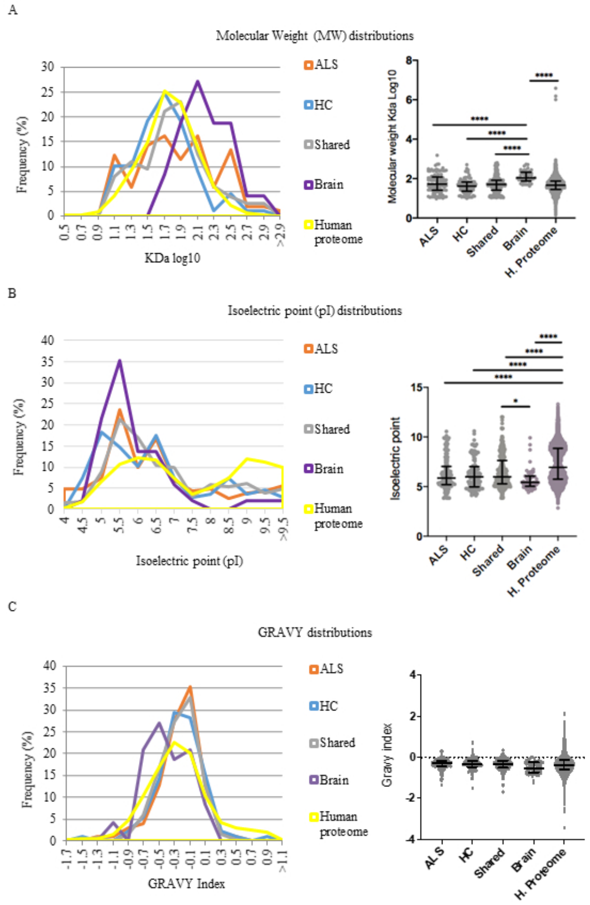

Figure 3. Aggregation propensity of proteins in circulating and brain protein aggregates from ALS and HC compared to the Human proteome.

Molecular weight (MW) (A), isoelectric point (pI) (B) and hydrophobicity (GRAVY index) (C), known to affect aggregation propensity, are compared across proteins found to be expressed only in ALS and HC CPA (ALS and HC respectively), proteins shared between ALS and HC CPA datasets (Shared), proteins within brain aggregates (Brain) and in the entire human proteome. The distribution plots show the dispersion of the samples with relative frequency, while the violin plots show median and interquartile ranges. Statistical analysis was performed using one-way ANOVA, Kruskal-Wallis test with Dunn's multiple comparison as post-test for group analysis with * expressing the level of significance (*: p = 0.0251; ****: p < 0.0001).

210x297mm (96 x 96 DPI)

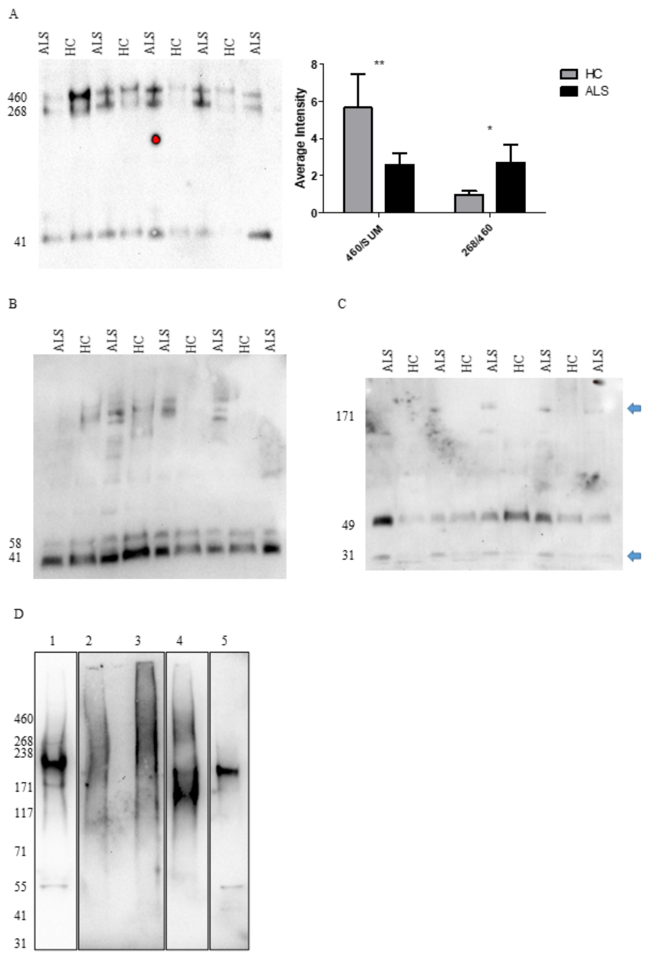

Figure 4. Western blot analysis of neurofilament heavy chain (NfH) within circulating protein aggregates (CPA) after proteases digestion.

Undigested CPA (A) show NfH bands at 460, 268 and 41 KDa (268 KDa is NfH expected molecular weight). The ratio between the 460 KDa band and the the sum of all NfH band intensities (SUM, 460/SUM) is higher in HC (p= 0.032), while the ratio between the 268 and 460 bands (268/460) is higher in ALS (p= 0.018). Calpain digestion (B) shows 58 and 41 KDa bands in all samples with no difference in expression. The enterokinase digestion profile of NfH (C) shows a 49 KDa band uniformly expressed across samples and additional 171 and 31 KDa bands only in ALS patients (blue arrows). Undigested NfH in ALS brain protein aggregates (BPA; D, lane 1) and after digestion with chymotrypsin (lane 2), enterokinase (lane 3), calpain (lane 4) and brain lysate lane 5. To maximise band visualization, time exposure was for lane 1 at 10.1 seconds, lane 4 and 5 at 58.4 seconds and lane 2 and 3 at 278.8 seconds. In D, the lanes have been rearranged to simplify comparison with CPA data shown in A, B and C. The original blot is included in the Supplementary Material.

210x297mm (96 x 96 DPI)

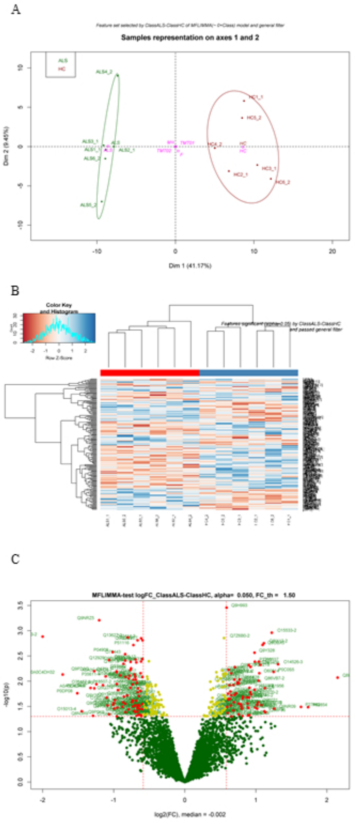

Figure 5. TMTcalibrator™ proteomic analysis.
(A) Principal component analysis (PCA) showing a separation between the ALS and HC experimental groups regulated features at protein level. Dimension 1 or the variance between the two experimental groups (ALS and HC) is 41.17% of the entire variance; dimension 2 or variance between 10plexes (TMT01 and TMT02) is 9.45% of the entire variance. (B) Heatmap showing the distribution of the regulated features and their clustering. The regulated features are distributed vertically, reported as Uniprot IDs on the right-hand side and relative clustering on the left-hand side. Analytical samples are distributed horizontally, with sample names at the bottom and relative clustering at the top of the heatmaps. The color key histogram at the top left side shows the distribution of the features and the heatmap color coding. (C) The volcano plot shows the distribution of the proteins identified by TMT proteomic study according to their fold change (FC) expressed as log2 (fold change ALS/HC) (logFC) in the x axis and according to p-value expressed as –log10 (p-value) in the y axis. Protein groups were considered regulated if p-value < 0.05 and logFC < -0.58 or > 0.58. Red dots are regulated features, yellow dots are features with a significant p-value (p < 0.05) and logFC between -0.58 and 0.58 while green dots are not regulated protein groups (p > 0,05). Uniprot IDs are

reported beside the dots with significant p-value.

210x297mm (96 x 96 DPI)

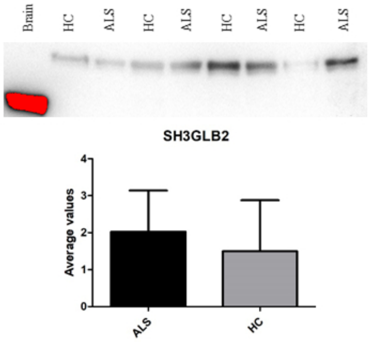

Figure 6. Western blot analysis of Endophilin-B2 (SH3GLB2) in plasma CPA from ALS patients and healthy controls.

Samples were normalized to HC6 density and the average values with relative standard deviation for the ALS (n=4) and Control (n=4) groups were plotted onto the chart. A brain lysate sample is also included (1st lane, red band, indicating signal saturation) which showed an endophilin-B2 band at a lower molecular weight than the bands detected in CPA. Immunodetection confirmed the SH3GLB2 higher level of expression in the ALS CPA compared to control (logFC= 0.34), but without being statistically significant (p= 0.57).

210x297mm (96 x 96 DPI)

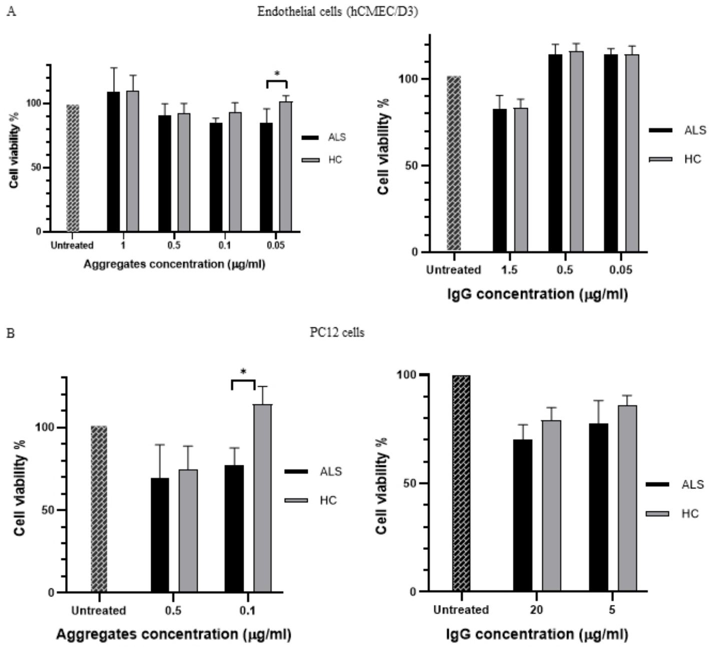

Figure 7. Cell viability after treatment with circulating protein aggregates (CPA), proteins solubilized from CPA and immunoglobulins extracted from the same plasma samples.
The figure shows the percentage of endothelial (hCMEC/D3) and PC12 living cells (A and B) after treatment with different concentrations of CPA and IgG from ALS and HC. Cells treated with ALS CPA showed a statistically significant lower cell viability compared to HC CPA treated cells at 0,05 µg/ml (p= 0.031; endothelial cells, A) and at 0,1 µg/ml (p= 0.029; PC12 cells, B). IgG had minor effect on all cell type viability with no difference between ALS and HC. CPA proteins were solubilized with 8M urea before PC12 cells treatment. Significance was tested by two-way ANOVA and Tukey HSD test.

210x297mm (96 x 96 DPI)

Table 1. ALS risk genes included in the list of proteins identified by the TMTcalibrator™ workflow in CPA from ALS and HC and reported in the gene classifiers MalaCards Human Disease Database (https://www.malacards.org/card/amyotrophic_lateral_sclerosis_1#RelatedGenes-table) (Database, n.d.). Among a total of 38 ALS elite genes reported in the MalaCards Human Disease Database (those more likely to cause the disease), 24 were detected in the list of proteins generated by the TMTcalibrator™ experiment.

[A]Gene	[B]Uniprot ID	[C]Protein name	[D]Unique peptides	[E]logFC	[F]p-value
**FUS**	P35637-2	Isoform Short of RNA-binding protein FUS	5	0.564	6.96e^{-03}
**NEFH**	P12036	Neurofilament heavy polypeptide	27	0.298	6.36e^{-02}
**OPTN**	Q96CV9	Optineurin	11	-0.243	9.58e^{-02}
**UNC13A**	Q9UPW8	Protein unc-13 homolog A	11	-0.326	9.75e^{-02}
**PON2**	Q15165-3	Isoform 3 of Serum paraoxonase/arylesterase 2	1	0.292	1.46e^{-01}
**ANG**	P03950	Angiogenin	1	-0.595	1.67e^{-01}
**CHMP2B**	Q9UQN3	Charged multivesicular body protein 2b	1	0.492	1.90e^{-01}
**VCP**	P55072	Transitional endoplasmic reticulum ATPase	65	-0.514	2.16e^{-01}
**ATXN2**	Q99700-2	Isoform 2 of Ataxin-2	2	-0.445	2.51e^{-01}
**ANXA11**	P50995-2	Isoform 2 of Annexin A11	16	0.150	3.01e^{-01}
**SOD1**	P00441	Superoxide dismutase [Cu-Zn]	8	0.176	3.39e^{-01}
**ERBB4**	Q15303-4	Isoform JM-B CYT-2 of Receptor tyrosine-protein kinase erbB-4	1	-0.284	3.70e^{-01}
**TARDBP**	Q13148	TAR DNA-binding protein 43	2	0.180	3.85e^{-01}

For Review Only

[A]Gene	[B]Uniprot ID	[C]Protein name	[D]Unique peptides	[E]logFC	[F]p-value
**SQSTM1**	Q13501	Sequestosome-1	2	-0.280	4.00e^{-01}
**MATR3**	P43243	Matrin-3	16	0.127	4.03e^{-01}
**PFN1**	P07737	Profilin-1	14	0.136	4.08e^{-01}
**VAPB**	O95292	Vesicle-associated membrane protein-associated protein B/C	10	-0.094	4.31e^{-01}
**EPHA4**	P54764	Ephrin type-A receptor 4	13	-0.082	5.37e^{-01}
**PON1**	P27169	Serum paraoxonase/arylesterase 1	14	0.123	6.07e^{-01}
**TAF15**	Q92804-2	Isoform Short of TATA-binding protein-associated factor 2N	3	0.067	6.99e^{-01}
**UBQLN2**	Q9UHD9	Ubiquilin-2	4	-0.065	6.99e^{-01}
**HNRNPA1**	P09651-3	Isoform 2 of Heterogeneous nuclear ribonucleoprotein A1	8	-0.066	7.50e^{-01}
**DCTN1**	Q14203-6	Isoform 6 of Dynactin subunit 1	2	0.020	9.34e^{-01}
**TBK1**	Q9UHD2	Serine/threonine-protein kinase TBK1	5	-0.004	9.86e^{-01}

For Review Only

A: the gene symbol used to represent a gene

B: Uniprot database protein identifier

C: protein full name recommended by Uniprot

D: number of peptide sequences unique to a protein group

E: relative quantification with value expressed as log2(ALS/HC) intensities

F: statistical significance for differential regulation between ALS and HC experimental groups

Supplementary Information for

Analysis of circulating protein aggregates reveals pathological hallmarks of amyotrophic lateral sclerosis

Rocco Adiutori*[1], Fabiola Puentes[1], Michael Bremang[2], Vittoria Lombardi[1], Irene Zubiri[1], Emanuela Leoni[3], Johan Aarum[4], Denise Sheer[5], Simon McArthur[6], Ian Pike[2], Andrea Malaspina*[1]

Affiliation:
1: Centre for Neuroscience and Trauma, Blizard Institute, Queen Mary University of London, 4 Newark Street, London, E1 2AT, UK.
2: Proteome Sciences plc, Hamilton House, Mabledon Place, London, WC1H 9BB, UK.
3: Proteome Sciences R&D GmbH & Co. KG, Altenhöferallee 3, Frankfurt am Main, 60438, Germany.
4: Department of Clinical Microbiology, Karolinska University Hospital, Stockholm, 171 76 Sweden.
5: Centre for Genomics and Child Health, Blizard Institute, Queen Mary University of London, 4 Newark Street, London, E1 2AT, UK.
6: Institute of Dentistry, Blizard Institute, Queen Mary University of London, 4 Newark Street, London, E1 2AT, UK.

* Corresponding authors: Rocco Adiutori, Andrea Malaspina.
Email: rocco.adiutori@qmul.ac.uk; andrea.malaspina@qmul.ac.uk

For Review Only

Study participant: cohort composition, clinical and demographic information

Supplementary Table 1. Clinical and demographic features of the amyotrophic lateral sclerosis (ALS) and healthy controls (HC) individuals selected for LC-MS proteomic analysis of pooled plasma samples.

Group	M:F	Ethnicity	Age at visit (years)	Diagnostic classification	Site of Onset	ALSFRS-R	Progression rate at visit
**HC**	3:3	Caucasian (100%)	58,7	NA	NA	NA	NA
**ALS**	5:1	Caucasian (83,3%), Afro-Caribbean (16,7%)	65,5	Definite ALS (33,3%), Possible ALS (33,3%), Probable ALS (16,7%), Suspected ALS (16,7%)	Limb (33,3%), Bulbar (16,7%), Respiratory (16,7%), Bulbar/Respiratory (33,3%)	39	1,974

M:F: males (M) and females (F) ratio

Diagnostic classification: diagnosis of ALS according to the El-Escorial criteria ([1])

Site of onset: anatomic site of disease onset (e.g. limb vs bulbar)

ALS Functional Rating Scale revised: level of neurological impairment across different clinical domains (1-48, higher neurological impairment with lower values)

Progression rate at last visit: calculated as 48 - ALSFRS-R score at last visit/disease duration from onset of symptoms to sampling time expressed in months

For Review Only

Supplementary Table 2. Clinical and demographic features of the amyotrophic lateral sclerosis (ALS) and healthy controls (HC) individuals selected for circulating protein aggregates (CPA) digestion and TMTcalibrator™ proteomic analysis.

Group	M:F	Ethnicity	Age at visit (years)	Diagnostic classification	Site of Onset	ALSFRS-R	Progression rate at visit
**HC**	3:3	Caucasian (100%)	64.2	NA	NA	NA	NA
**ALS**	3:3	Caucasian (100%)	63.8	Definite ALS (100%)	Limb (100%)	29	0.961

M:F: males (M) and females (F) ratio

Diagnostic classification: diagnosis of ALS according to the El-Escorial criteria ([1])

Site of onset: anatomic site of disease onset (e.g. limb vs bulbar)

ALS Functional Rating Scale revised: level of neurological impairment across different clinical domains (1-48, higher neurological impairment with lower values)

Progression rate at last visit: calculated as 48 - ALSFRS-R score at sampling time at last visit/disease duration from onset of symptoms to sampling time at last visit expressed in months

For Review Only

Supplementary Table 3. Clinical and demographic features of the amyotrophic lateral sclerosis (ALS) and healthy controls (HC) individuals selected for validation experiments by western blot.

Group	M:F	Ethnicity	Age at visit (years)	Diagnostic classification	Site of Onset	ALSFRS-R	Progression rate at visit
**HC**	2:4	Caucasian (100%)	61.8	NA	NA	NA	NA
**ALS**	2:2	Caucasian (100%)	63.9	Definite ALS (100%)	Limb (100%)	27	1.082

M:F: males (M) and females (F) ratio

Diagnostic classification: diagnosis of ALS according to the El-Escorial criteria ([1])

Site of onset: anatomic site of disease onset (e.g. limb vs bulbar)

ALS Functional Rating Scale revised: level of neurological impairment across different clinical domains (1-48, higher neurological impairment with lower values)

Progression rate at last visit: calculated as 48 - ALSFRS-R score at last visit/disease duration from onset of symptoms to last visit expressed in months

For Review Only

Protein aggregates enrichment from blood and brain.

Plasma samples kept at -80°C were thawed on ice. Triton X-100 was added to a final concentration of 2%. The mixture was incubated for 10 minutes at room temperature and centrifuged at 21000xg for 15 minutes. The supernatant was placed onto a sucrose cushion (1 M sucrose, 50 mM Tris-HCl pH 7.4, 1 mM EDTA and 2% Triton X-100) to form two different phases. Ultracentrifugation (UC) was performed for 2 hours at 50000 rpm (167829.2xg) at 4°C, using a Sorvall Discovery 100SE (TFT 80.2 rotor). Supernatant was discarded and pellet, resuspended, washed in PBS (1.5 NaCl) and vortexed for 30 seconds. An additional 40 minutes UC was undertaken for CPA pellet enrichment. The UC final product was resuspended in experimental procedure-specific media including 1) a buffer suitable for the analysis of aggregates resistance to digestion and 2) SysQuant Buffer, 8M urea, phosphatase inhibitor (PhosSTOP™, Merck) and protease inhibitor (cOmplete™, Merck).

For transmission electron microscopy (TEM), the final UC pellet was resuspended in 500 µl PBS and subjected to an additional washing step. The supernatant was then discarded, the pellets re-suspended in 100 µl double distilled water (ddH2O) and transferred into a clean tube to be sonicated on ice at max power for 5 minutes (Diogenode, Bioruptor) in order to disrupt possible formations caused by the high g-force in UC. Enriched fractions were stored at -80°C for TEM analysis.

For Review Only

Correction factors for semi-quantitative analysis of neurofilament heavy chain (NfH) in circulating protein aggregates (CPAs) before and after digestion with proteases.

For semi-quantitative analysis of ALS and HC digested products, band intensities of the related undigested samples were used as reference to adjust for differences in the total protein content loading across samples (SDS-PAGE was subjected to zinc staining as described in the methods section).

CPA digestion products (as described in "Circulating and brain protein aggregates protease digestion") were resolved in 3-8% tris-acetate SDS-PAGE and visualized by Zinc staining (Life Technologies). Images acquisition (Chemi-Doc Camera, Bio-Rad) and processing (ImageJ) was performed using the Analyze\Gels\Plot lanes function and band intensities was obtained using the size marker (HiMark™ Pre-stained Protein Standard) as reference. Correction factors to adjust loading differences across samples included the ratio between the sum of all band intensities for each undigested sample and the sum of the marker total band intensities (from the same gel; Supplementary material, Table S5). Resistance to proteases of NfH within CPA was evaluated by western blotting. Band intensities of NfH digested products were corrected using NfH undigested samples band intensities as reference and normalized for loading volumes differences as reported in the supplementary material (Table S6).

The sum of the intensity of all bands in each lane was compared across samples (Table S5). The size marker variation across gels showed a coefficient of variation (CV) of 6.3% while the undigested CPA samples showed a CV of >10% (Table S5). To eliminate this variability, a correction factor was generated as the ratio between the intensities of the undigested samples and the sum of the intensities of the Marker in the same gel (Table S6). These factors were applied to the semi-quantitative analysis of NfH expression after CPA digestion.

For Review Only

Supplementary Table 4. Marker, undigested ALS and HC samples: sum of the band intensities.

Total intensity for:	Gel1 (ALS1_HC1)	Gel2 (ALS2_HC2)	Gel3 (ALS3_HC3)	Gel4 (ALS4_HC4)	Gel5 (ALC5_HC5)	Gel6 (HC6)	Mean	St. Dev.	CV%
**Marker**	107704	109638	102576	114133	100891	95666	105102	6664	6.3
**ALS**	62481	57628	58192	55143	46977	-	56084	5735	10.2
**HC**	40800	59041	39941	46063	40622	50107	46095	7477	16.2

The Marker shows low CV% suggesting equal loading. A higher CV% was obtained for ALS and in particular for HC samples, suggesting uneven loading.

Total bands intensity (black color code): sum of the intensities of all bands in each ALS and HC undigested sample and in the gel marker lane (ImageJ) for all 6 gels. St. Dev. (grey color code): mean, standard deviation; CV% 9grey color code): coefficient of variation expressed in percentage.

For Review Only

Supplementary Table 5. Correction factors (CFs) for the digested ALS and HCs samples calculated using undigested samples and marker total intensities as reported in Table 4.

	ALS1*	ALS2*	ALS3*	ALS4*	ALS5*	HC3	HC4	HC5	HC6
**Correction Factors**	0.58	0.56	0.57	0.48	0.47	0.39	0.40	0.40	0.52

The correction factors (CFs) were applied to quantify differences in CPA band intensities of digested samples obtained by SDS-PAGE. Each band was first normalized with the marker band intensity closer to the band molecular weight (MW) and then divided by the specific sample/lane CF. ALS1*-5* and HC3-6 indicates the samples used in this experiment with relative CF.

For Review Only

Antibodies used in the study

Supplementary Table 6. List of antibodies used for western blotting, including primary and secondary antibodies.

Primary antibodies	ID antibody	Species	Provider	Working condition
**anti-Neurofilament heavy (NfH)**	N4142	rabbit	Sigma-Aldrich	1:1000 in blocking buffer
**anti-TAR DNA-binding protein 43 (TDP-43)**	G400	rabbit	New England Biolabs	1:1000 in blocking buffer
**anti-Ubiquitinylated proteins**	clone FK1 | 04-262	mouse	Millipore	1:1000 in blocking buffer
**anti-Fibromodulin (FMOD)**	CSB-PA008755GA01HU	rabbit	Generon Ltd	1:1000 in blocking buffer
**anti-Glypican-4 (GCP4)**	LS-C375826	rabbit	Source BioScience UK	1:2000 in blocking buffer
**anti-Byglican (BGN)**	HPA003157	rabbit	Cambridge Bioscience	1:250 in TBS-T 0.1%, 5%BSA
**anti-Cation-dependent mannose-6-phosphate receptor (M6PR)**	ARP43519_T100	rabbit	Insight Biotechnology	1:500 in TBS-T 0.1%, 5%BSA
**anti-Protein DJ-1 (PARK7)**	HPA004190	rabbit	Cambridge Bioscience	1:250 in TBS-T 0.1%, 5%BSA
**anti-Endophilin-B2 (SH3GLB2)**	H00056904-B01P	mouse	Bio-Techne	1:500 in TBS-T 0.1%, 5%BSA
**anti-Rabbit IgG (HRP conjugated)**	P021702-2	swine	DAKO	1:50000 or 1:20000 in blocking buffer depending on the primary antibody
**anti-Mouse IgG (HRP conjugated)**	A28177	goat	Thermo Fischer Scientific	1:20000 in blocking buffer

For Review Only

Supplementary figures – TMTcalibrator™

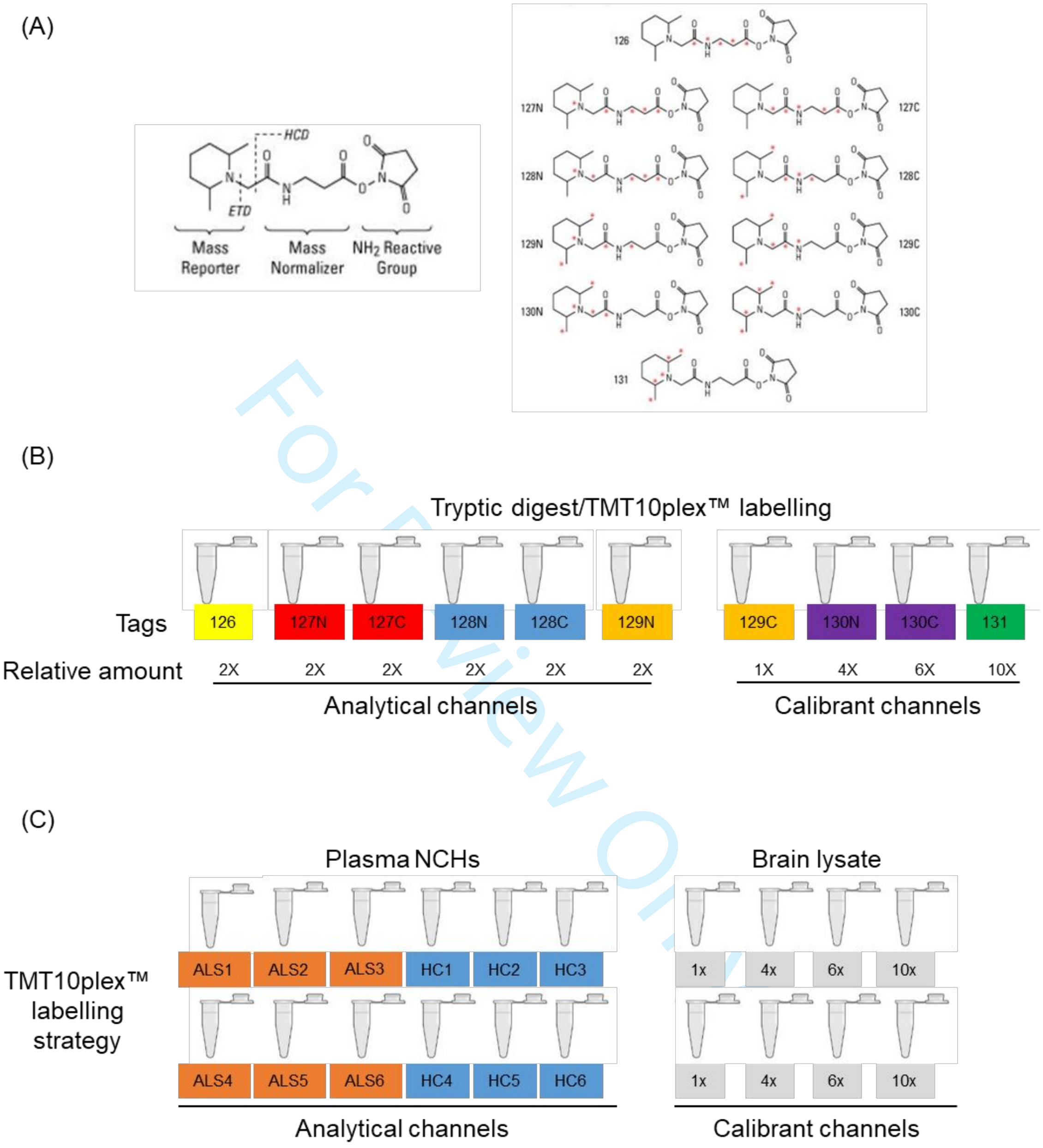

Supplementary Figure 1. TMTcalibrator™ experimental design.

(A) Tandem Mass Tag (TMT) reagents with relative masses and isotope position. (B) General 10plex labelling layout after trypsin digestion of the samples; analytical samples and calilbrants are mixed in a specific ratio that enhances detection by LC-MS/MS of low abundant peptides in the analytical channels thanks to the high calibrant content. (C) Labelling strategy in two 10plexes LC-MS/MS runs which includes Circulating Protein aggregates (CPA) from amyotrophic lateral sclerosis (ALS) patients and from healthy controls (HC) in the analytical channels (orange and blue colour codes) and a mixture (1:1) of brains (Precentral gyrus) lysates from two different ALS patients in the calibrant channels (grey colour code).

For Review Only

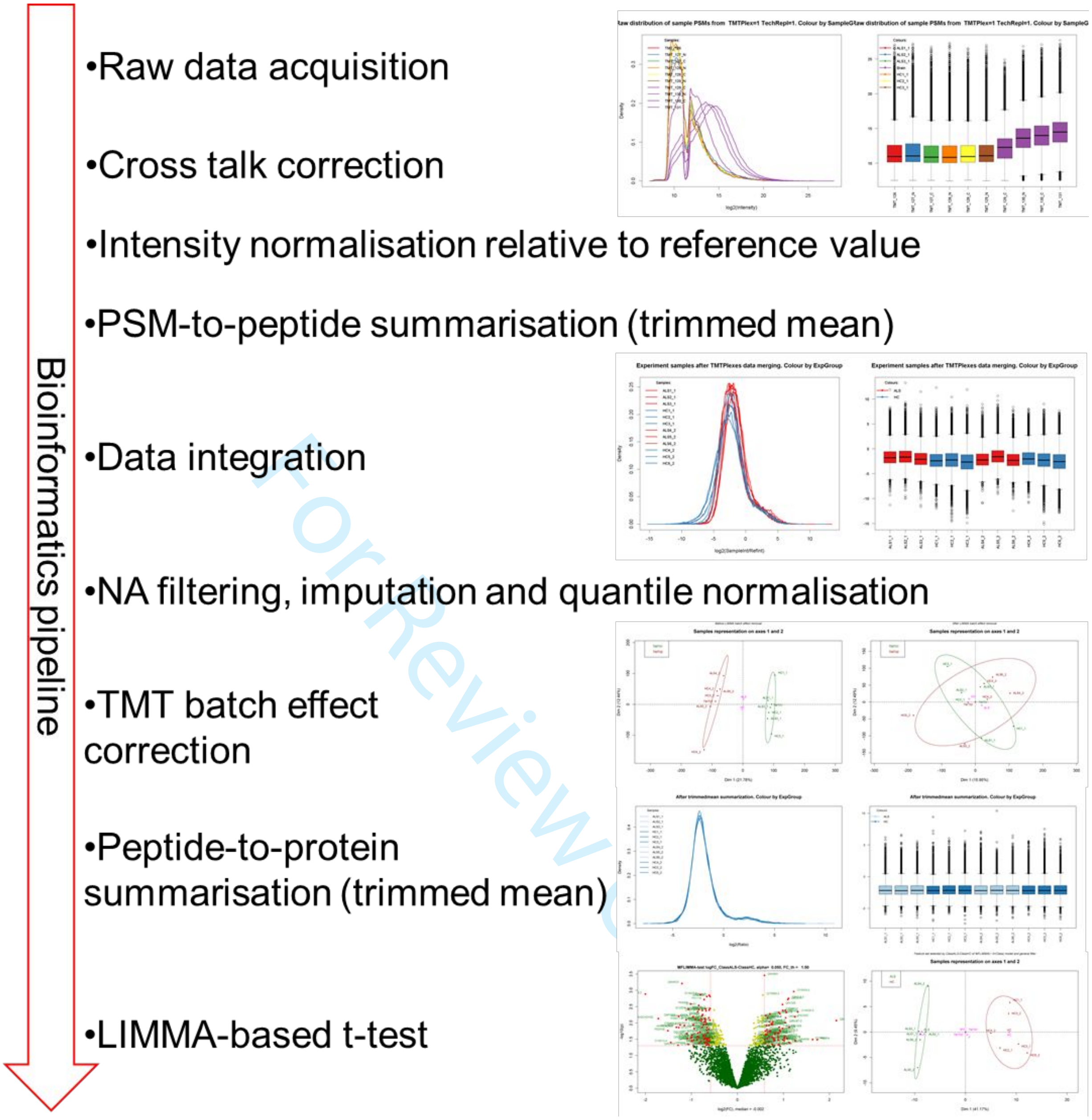

Supplementary Figure 2. TMTcalibrator™: bioinformatic pipeline.

After MS/MS spectra (raw data) acquisition, the intensity of each channel was corrected for background and cross-talking between tags in the second mass spectrometer (MS2). Intensity values of the detected Peptide-Spectrum Matches (PSMs) were normalised with a reference value generated as the average of the Calibrant channels and this was followed by PSM-to-peptide summarisation defined as “trimmed mean”. Data points considered as outliers in each analytical sample were removed stabilizing the mean before merging the data obtained from the two 10plexes. Then, “not available data points (NA)” filtering, imputation and quantile normalization were performed on the merged data set, so that it was possible to perform a Principal Component Analysis (PCA) on the data acquired. It was also possible to evaluate the TMT batch effect within linear models for microarray data (LIMMA). After peptide-to-protein summarisation, a statistically significant difference of expression (p-Value < 0.05, Fold Change threshold = 1.5) of the protein groups identified was tested using a LIMMA-based t-test .

Functional Analysis Tool (FAT) results: top10 regulated pathways in the TMTcalibrator™ experiment

Supplementary Table 7. FAT analysis of the ALS vs HC TMT proteomics: top10 regulated pathways.

Pathway description	Number of genes in the category	p-Value	Median logFC
**Lipoprotein metabolism [Reactome]**	27	$4.11e^{-04}$	0.19
**Dopamine Neurotransmitter Release Cycle [Reactome]**	15	$4.53e^{-04}$	0.24
**DCC mediated attractive signaling [Reactome]**	8	$6.65e^{-04}$	0.36
**Plasma lipoprotein assembly, remodeling, and clearance [Reactome]**	25	$8.03e^{-04}$	0.19
**Plasma lipoprotein assembly [Reactome]**	13	$1.65e^{-03}$	0.29
**Netrin-1 signaling [Reactome]**	15	$4.80e^{-03}$	0.20
**Lipid digestion, mobilization, and transport [Reactome]**	36	$5.25e^{-03}$	0.11
**Oncostatin M Signaling Pathway [Wikipathways]**	21	$8.09e^{-03}$	-0.13
**Plasma lipoprotein remodeling [Reactome]**	9	$8.16e^{-03}$	0.19
**CD28 dependent Vav1 pathway [Reactome]**	6	$8.35e^{-03}$	0.28

For Review Only

Pathway description: name of the pathway identified (reference database in square brackets, e.g. [Reactome])

Number of genes in the category: genes included in the given pathway in Homo sapiens

p-Value: statistical significance calculated by Mann-Whitney U test

Median logFC: median value of expression (logFC values) of the proteins included in the given pathway in Homo sapiens

Highly regulated proteins in the TMTcalibrator™ dataset

Supplementary Table 8. List of the proteins identified in the TMTcalibrator™ dataset with unique peptides ≥ 2, logarithmic fold change (LogFC) < -0.693 or > 0.693 and statistically significant (p-value < 0.05).

Uniprot ID	Gene name	Protein name	Unique peptides	logFC	p-value
Q9P2W3	GNG13	Guanine nucleotide-binding protein G(I)/G(S)/G(O) subunit gamma-13	2	-1.276	$6.32e^{-03}$
A0A0C4DH67	IGKV1-8	Immunoglobulin kappa variable 1-8	2	-1.273	$1.39e^{-02}$
O75157-2	TSC22D2	Isoform 2 of TSC22 domain family protein 2	2	-1.245	$1.15e^{-02}$
Q9NRZ5	AGPAT4	1-acyl-sn-glycerol-3-phosphate acyltransferase delta	2	-1.205	$6.21e^{-04}$
P35579	MYH9	Myosin-9	82	-1.000	$2.70e^{-02}$
Q9Y3E2	BOLA1	BolA-like protein 1	2	-0.994	$3.48e^{-02}$
Q5QJ74	TBCEL	Tubulin-specific chaperone cofactor E-like protein	2	-0.941	$1.50e^{-02}$
Q53GQ0	HSD17B12	Very-long-chain 3-oxoacyl-CoA reductase	5	-0.910	$7.70e^{-03}$
Q9UHI5	SLC7A8	Large neutral amino acids transporter small subunit 2	2	-0.867	$2.62e^{-02}$
Q16718	NDUFA5	NADH dehydrogenase [ubiquinone] 1 alpha subcomplex subunit 5	2	-0.862	$4.52e^{-03}$
Q9BU02	THTPA	Thiamine-triphosphatase	2	-0.838	$3.92e^{-03}$
O75487	GPC4	Glypican-4	2	-0.834	$4.10e^{-02}$
O15084	ANKRD28	Serine/threonine-protein phosphatase 6 regulatory ankyrin repeat subunit A	3	-0.809	$1.92e^{-02}$
P29144	TPP2	Tripeptidyl-peptidase 2	37	-0.789	$3.62e^{-02}$
A8MWD9	SNRPGP15	Putative small nuclear ribonucleoprotein G-like protein 15	3	-0.768	$1.36e^{-02}$
Q9NX63	CHCHD3	MICOS complex subunit MIC19	10	-0.763	$4.14e^{-03}$
Q53H82	LACTB2	Endoribonuclease LACTB2	2	-0.756	$5.56e^{-03}$
P50238	CRIP1	Cysteine-rich protein 1	3	-0.742	$2.61e^{-02}$
P00813	ADA	Adenosine deaminase	3	-0.733	$1.16e^{-02}$
P51116	FXR2	Fragile X mental retardation syndrome-related protein 2	3	-0.723	$1.93e^{-03}$
P40855	PEX19	Peroxisomal biogenesis factor 19	2	-0.722	$9.35e^{-03}$
P38919	EIF4A3	Eukaryotic initiation factor 4A-III	6	-0.709	$7.35e^{-03}$

For Review Only

Uniprot ID	Gene name	Protein name	Unique peptides	logFC	p-value
Q9BYH1-3	SEZ6L	Isoform 2 of Seizure 6-like protein	4	-0.697	$8.24e^{-03}$
Q9BV20	MRI1	Methylthioribose-1-phosphate isomerase	4	-0.692	$3.74e^{-03}$
P52788	SMS	Spermine synthase	5	-0.689	$2.44e^{-02}$
Q99470	SDF2	Stromal cell-derived factor 2	2	-0.682	$4.26e^{-03}$
Q9NPB8	GPCPD1	Glycerophosphocholine phosphodiesterase GPCPD1	2	0.683	$1.19e^{-02}$
O14514	BAI1	Brain-specific angiogenesis inhibitor 1	2	0.697	$9.31e^{-03}$
Q6NXE6-2	ARMC6	Isoform 2 of Armadillo repeat-containing protein 6	2	0.700	$1.87e^{-02}$
Q99729-3	HNRNPAB	Isoform 3 of Heterogeneous nuclear ribonucleoprotein A/B	3	0.706	$5.29e^{-03}$
Q9HD89	RETN	Resistin	4	0.754	$2.78e^{-02}$
P55083	MFAP4	Microfibril-associated glycoprotein 4	2	0.761	$1.09e^{-02}$
Q06828	FMOD	Fibromodulin	3	0.770	$9.28e^{-03}$
P45984-3	MAPK9	Isoform Beta-1 of Mitogen-activated protein kinase 9	2	0.789	$2.87e^{-02}$
Q9Y3C8	UFC1	Ubiquitin-fold modifier-conjugating enzyme 1	2	0.803	$3.52e^{-02}$
Q9NRA0-2	SPHK2	Isoform 2 of Sphingosine kinase 2	2	0.897	$3.39e^{-02}$
P04275	VWF	von Willebrand factor	127	0.910	$4.69e^{-02}$
P16519-2	PCSK2	Isoform 2 of Neuroendocrine convertase 2	3	0.911	$1.35e^{-02}$
P35219	CA8	Carbonic anhydrase-related protein	2	0.925	$5.23e^{-03}$
Q9HAU0-2	PLEKHA5	Isoform 2 of Pleckstrin homology domain-containing family A member 5	2	0.933	$1.84e^{-02}$
Q9Y328	NSG2	Neuron-specific protein family member 2	2	0.981	$2.73e^{-03}$
Q00537	CDK17	Cyclin-dependent kinase 17	2	1.043	$4.11e^{-03}$
O60814	HIST1H2BK	Histone H2B type 1-K	2	1.049	$6.97e^{-03}$
P49406	MRPL19	39S ribosomal protein L19, mitochondrial	2	1.062	$4.76e^{-03}$
Q8IX12-2	CCAR1	Isoform 2 of Cell division cycle and apoptosis regulator protein 1	2	1.113	$1.77e^{-03}$
Q96F86	EDC3	Enhancer of mRNA-decapping protein 3	2	1.134	$4.15e^{-02}$
O15533-2	TAPBP	Isoform 2 of Tapasin	2	1.224	$1.09e^{-03}$

For Review Only

Uniprot ID	Gene name	Protein name	Unique peptides	logFC	p-value
P27449	ATP6V0C	V-type proton ATPase 16 kDa proteolipid subunit	2	1.635	$3.23e^{-02}$

Uniprot ID: Uniprot database protein identifier

Gene name: the recommended gene symbol used to officially represent a gene

Protein name: protein full name recommended by Uniprot

Unique peptides: number of peptide sequences unique to a protein group

logFC: relative quantification with value expressed as log2(ALS/HC) intensities

p-value: statistical significance of the differential regulation between ALS and HC experimental groups

For Review Only

SI References

1. Ludolph A, Drory V, Hardiman O, et al. A revision of the El Escorial criteria - 2015. *Amyotroph Lateral Scler Frontotemporal Degener*. 2015;16(5-6):291-292. doi:10.3109/21678421.2015.1049183

For Review Only
