# Supplementary material for: Analysis of circulating protein aggregates as a route of investigation into neurodegenerative disorders
Source: Brain Commun. 2021 Jul 9;3(3):fcab148. doi: 10.1093/braincomms/fcab148 (PMC8361415; doi:10.1093/braincomms/fcab148)
Supplement: fcab148_Supplementary_Data [file fcab148_supplementary_data.zip › supplementary information_Brain Communications_submission_20210622.docx]

Supplementary Information for

**Analysis of circulating protein aggregates as a route of investigation into neurodegenerative disorders**

Rocco Adiutori*^1^, Fabiola Puentes^1^, Michael Bremang^2^, Vittoria Lombardi^1^, Irene Zubiri^1^, Emanuela Leoni^3^, Johan Aarum^4^, Denise Sheer^5^, Simon McArthur^6^, Ian Pike^2^, Andrea Malaspina*^1^

Affiliation:

1: Centre for Neuroscience and Trauma, Blizard Institute, Queen Mary University of London, 4 Newark Street, London, E1 2AT, UK.

2: Proteome Sciences plc, Hamilton House, Mabledon Place, London, WC1H 9BB, UK.

3: Proteome Sciences R&D GmbH & Co. KG, Altenhöferallee 3, Frankfurt am Main, 60438, Germany.

4: Department of Clinical Microbiology, Karolinska University Hospital, Stockholm, 171 76 Sweden.

5: Centre for Genomics and Child Health, Blizard Institute, Queen Mary University of London, 4 Newark Street, London, E1 2AT, UK.

6: Institute of Dentistry, Blizard Institute, Queen Mary University of London, 4 Newark Street, London, E1 2AT, UK.

* Corresponding authors: Rocco Adiutori, Andrea Malaspina.

Correspondence to: Prof. Andrea Malaspina, Centre for Neuroscience and Trauma, Blizard Institute, Queen Mary University of London, 4 Newark Street, London, E1 2AT, UK, a.malaspina@qmul.ac.uk

Correspondence may also be sent to: Rocco Adiutori, Centre for Neuroscience and Trauma, Blizard Institute, Queen Mary University of London, 4 Newark Street, London, E1 2AT, UK, rocco.adiutori@qmul.ac.uk

**Study participant: cohort composition, clinical and demographic information**

Supplementary Table 1. Clinical and demographic features of the amyotrophic lateral sclerosis (ALS) and healthy controls (HC) individuals selected for LC-MS proteomic analysis of pooled plasma samples.

| **Group** | **M:F** | **Ethnicity** | **Age at visit (years)** | **Diagnostic classification** | **Site of Onset** | **ALSFRS-R** | **Progression rate at visit** |
| --- | --- | --- | --- | --- | --- | --- | --- |
| **HC** | 3:3 | Caucasian (100%) | 58,7 | NA | NA | NA | NA |
| **ALS** | 5:1 | Caucasian (83,3%), Afro-Caribbean (16,7%) | 65,5 | Definite ALS (33,3%), Possible ALS (33,3%), Probable ALS (16,7%), Suspected ALS (16,7%) | Limb (33,3%), Bulbar (16,7%), Respiratory (16,7%), Bulbar/Respiratory (33,3%) | 39 | 1,974 |

M:F: males (M) and females (F) ratio

Diagnostic classification: diagnosis of ALS according to the El-Escorial criteria^1^

Site of onset: anatomic site of disease onset (e.g. limb vs bulbar)

ALS Functional Rating Scale revised: level of neurological impairment across different clinical domains (1-48, higher neurological impairment with lower values)

Progression rate at last visit: calculated as 48 - ALSFRS-R score at last visit/disease duration from onset of symptoms to sampling time expressed in months

Supplementary Table 2. Clinical and demographic features of the amyotrophic lateral sclerosis (ALS) and healthy controls (HC) individuals selected for circulating protein aggregates (CPA) digestion and TMTcalibrator™ proteomic analysis.

| **Group** | **M:F** | **Ethnicity** | **Age at visit (years)** | **Diagnostic classification** | **Site of Onset** | **ALSFRS-R** | **Progression rate at visit** |
| --- | --- | --- | --- | --- | --- | --- | --- |
| **HC** | 3:3 | Caucasian (100%) | 64.2 | NA | NA | NA | NA |
| **ALS** | 3:3 | Caucasian (100%) | 63.8 | Definite ALS (100%) | Limb (100%) | 29 | 0.961 |

M:F: males (M) and females (F) ratio

Diagnostic classification: diagnosis of ALS according to the El-Escorial criteria^1^

Site of onset: anatomic site of disease onset (e.g. limb vs bulbar)

ALS Functional Rating Scale revised: level of neurological impairment across different clinical domains (1-48, higher neurological impairment with lower values)

Progression rate at last visit: calculated as 48 - ALSFRS-R score at sampling time at last visit/disease duration from onset of symptoms to sampling time at last visit expressed in months

Supplementary Table 3. Clinical and demographic features of the amyotrophic lateral sclerosis (ALS) and healthy controls (HC) individuals selected for validation experiments by western blot.

| **Group** | **M:F** | **Ethnicity** | **Age at visit (years)** | **Diagnostic classification** | **Site of Onset** | **ALSFRS-R** | **Progression rate at visit** |
| --- | --- | --- | --- | --- | --- | --- | --- |
| **HC** | 2:4 | Caucasian (100%) | 61.8 | NA | NA | NA | NA |
| **ALS** | 2:2 | Caucasian (100%) | 63.9 | Definite ALS (100%) | Limb (100%) | 27 | 1.082 |

M:F: males (M) and females (F) ratio

Diagnostic classification: diagnosis of ALS according to the El-Escorial criteria^1^

Site of onset: anatomic site of disease onset (e.g. limb vs bulbar)

ALS Functional Rating Scale revised: level of neurological impairment across different clinical domains (1-48, higher neurological impairment with lower values)

Progression rate at last visit: calculated as 48 - ALSFRS-R score at last visit/disease duration from onset of symptoms to last visit expressed in months

Protein aggregates enrichment from blood and brain.

Plasma samples kept at -80°C were thawed on ice. Triton X-100 was added to a final concentration of 2%. The mixture was incubated for 10 minutes at room temperature and centrifuged at 21000xg for 15 minutes. The supernatant was placed onto a sucrose cushion (1 M sucrose, 50 mM Tris-HCl pH 7.4, 1 mM EDTA and 2% Triton X-100) to form two different phases. Ultracentrifugation (UC) was performed for 2 hours at 50000 rpm (167829.2xg) at 4°C, using a Sorvall Discovery 100SE (TFT 80.2 rotor). Supernatant was discarded and pellet, resuspended, washed in PBS (1.5 NaCl) and vortexed for 30 seconds. An additional 40 minutes UC was undertaken for CPA pellet enrichment. The UC final product was resuspended in experimental procedure-specific media including 1) a buffer suitable for the analysis of aggregates resistance to digestion and 2) SysQuant Buffer, 8M urea, phosphatase inhibitor (PhosSTOP™, Merck) and protease inhibitor (cOmplete™, Merck).

For transmission electron microscopy (TEM), the final UC pellet was resuspended in 500 µl PBS and subjected to an additional washing step. The supernatant was then discarded, the pellets re-suspended in 100 µl double distilled water (ddH2O) and transferred into a clean tube to be sonicated on ice at max power for 5 minutes (Diogenode, Bioruptor) in order to disrupt possible formations caused by the high g-force in UC. Enriched fractions were stored at -80°C for TEM analysis.

**Correction factors for semi-quantitative analysis of neurofilament heavy chain (NfH) in circulating protein aggregates (CPAs) before and after digestion with proteases.**

For semi-quantitative analysis of ALS and HC digested products, band intensities of the related undigested samples were used as reference to adjust for differences in the total protein content loading across samples (SDS-PAGE was subjected to zinc staining as described in the methods section).

CPA digestion products (as described in “Circulating and brain protein aggregates protease digestion”) were resolved in 3-8% tris-acetate SDS-PAGE and visualized by Zinc staining (Life Technologies). Images acquisition (Chemi-Doc Camera, Bio-Rad) and processing (ImageJ) was performed using the Analyze\Gels\Plot lanes function and band intensities was obtained using the size marker (HiMark™ Pre-stained Protein Standard) as reference. Correction factors to adjust loading differences across samples included the ratio between the sum of all band intensities for each undigested sample and the sum of the marker total band intensities (from the same gel; Supplementary material, Table S5). Resistance to proteases of NfH within CPA was evaluated by western blotting. Band intensities of NfH digested products were corrected using NfH undigested samples band intensities as reference and normalized for loading volumes differences as reported in the supplementary material (Table S6).

The sum of the intensity of all bands in each lane was compared across samples (Table S5). The size marker variation across gels showed a coefficient of variation (CV) of 6.3% while the undigested CPA samples showed a CV of >10% (Table S5). To eliminate this variability, a correction factor was generated as the ratio between the intensities of the undigested samples and the sum of the intensities of the Marker in the same gel (Table S6). These factors were applied to the semi-quantitative analysis of NfH expression after CPA digestion.

Supplementary Table 4. Marker, undigested ALS and HC samples: sum of the band intensities.

| Total intensity for: | Gel1 (ALS1_HC1) | Gel2 (ALS2_HC2) | Gel3 (ALS3_HC3) | Gel4 (ALS4_HC4) | Gel5 (ALC5_HC5) | Gel6 (HC6) | Mean | St. Dev. | CV% |
| --- | --- | --- | --- | --- | --- | --- | --- | --- | --- |
| Marker | 107704 | 109638 | 102576 | 114133 | 100891 | 95666 | 105102 | 6664 | 6.3 |
| ALS | 62481 | 57628 | 58192 | 55143 | 46977 | - | 56084 | 5735 | 10.2 |
| HC | 40800 | 59041 | 39941 | 46063 | 40622 | 50107 | 46095 | 7477 | 16.2 |

The Marker shows low CV% suggesting equal loading. A higher CV% was obtained for ALS and in particular for HC samples, suggesting uneven loading.

Total bands intensity (black color code): sum of the intensities of all bands in each ALS and HC undigested sample and in the gel marker lane (ImageJ) for all 6 gels. St. Dev. (grey color code): mean, standard deviation; CV% 9grey color code): coefficient of variation expressed in percentage.

Supplementary Table 5. Correction factors (CFs) for the digested ALS and HCs samples calculated using undigested samples and marker total intensities as reported in Table 4.

|  | ALS1* | ALS2* | ALS3* | ALS4* | ALS5* | HC3 | HC4 | HC5 | HC6 |
| --- | --- | --- | --- | --- | --- | --- | --- | --- | --- |
| Correction Factors | 0.58 | 0.56 | 0.57 | 0.48 | 0.47 | 0.39 | 0.40 | 0.40 | 0.52 |

The correction factors (CFs) were applied to quantify differences in CPA band intensities of digested samples obtained by SDS-PAGE. Each band was first normalized with the marker band intensity closer to the band molecular weight (MW) and then divided by the specific sample/lane CF. ALS1*-5* and HC3-6 indicates the samples used in this experiment with relative CF.

Antibodies used in the study

Supplementary Table 6. List of antibodies used for western blotting, including primary and secondary antibodies.

| **Primary antibodies** | **ID antibody** | **Species** | **Provider** | **Working condition** |
| --- | --- | --- | --- | --- |
| **anti-Neurofilament heavy (NfH)** | N4142 | rabbit | Sigma-Aldrich | 1:1000 in blocking buffer |
| **anti-TAR DNA-binding protein 43 (TDP-43)** | G400 | rabbit | New England Biolabs | 1:1000 in blocking buffer |
| **anti-Ubiquitinylated proteins** | clone FK1 \| 04-262 | mouse | Millipore | 1:1000 in blocking buffer |
| **anti-Fibromodulin (FMOD)** | CSB-PA008755GA01HU | rabbit | Generon Ltd | 1:1000 in blocking buffer |
| **anti-Glypican-4 (GCP4)** | LS-C375826 | rabbit | Source BioScience UK | 1:2000 in blocking buffer |
| **anti-Byglican (BGN)** | HPA003157 | rabbit | Cambridge Bioscience | 1:250 in TBS-T 0.1%, 5%BSA |
| **anti-Cation-dependent mannose-6-phosphate receptor (M6PR)** | ARP43519_T100 | rabbit | Insight Biotechnology | 1:500 in TBS-T 0.1%, 5%BSA |
| **anti-****Protein DJ-1 (PARK7)** | HPA004190 | rabbit | Cambridge Bioscience | 1:250 in TBS-T 0.1%, 5%BSA |
| **anti-Endophilin-B2 (SH3GLB2)** | H00056904-B01P | mouse | Bio-Techne | 1:500 in TBS-T 0.1%, 5%BSA |
| **anti-Rabbit IgG (HRP conjugated)** | P021702-2 | swine | DAKO | 1:50000 or 1:20000 in blocking buffer depending on the primary antibody |
| **anti-Mouse IgG (HRP conjugated)** | A28177 | goat | Thermo Fischer Scientific | 1:20000 in blocking buffer |

Supplementary figures – TMTcalibrator™


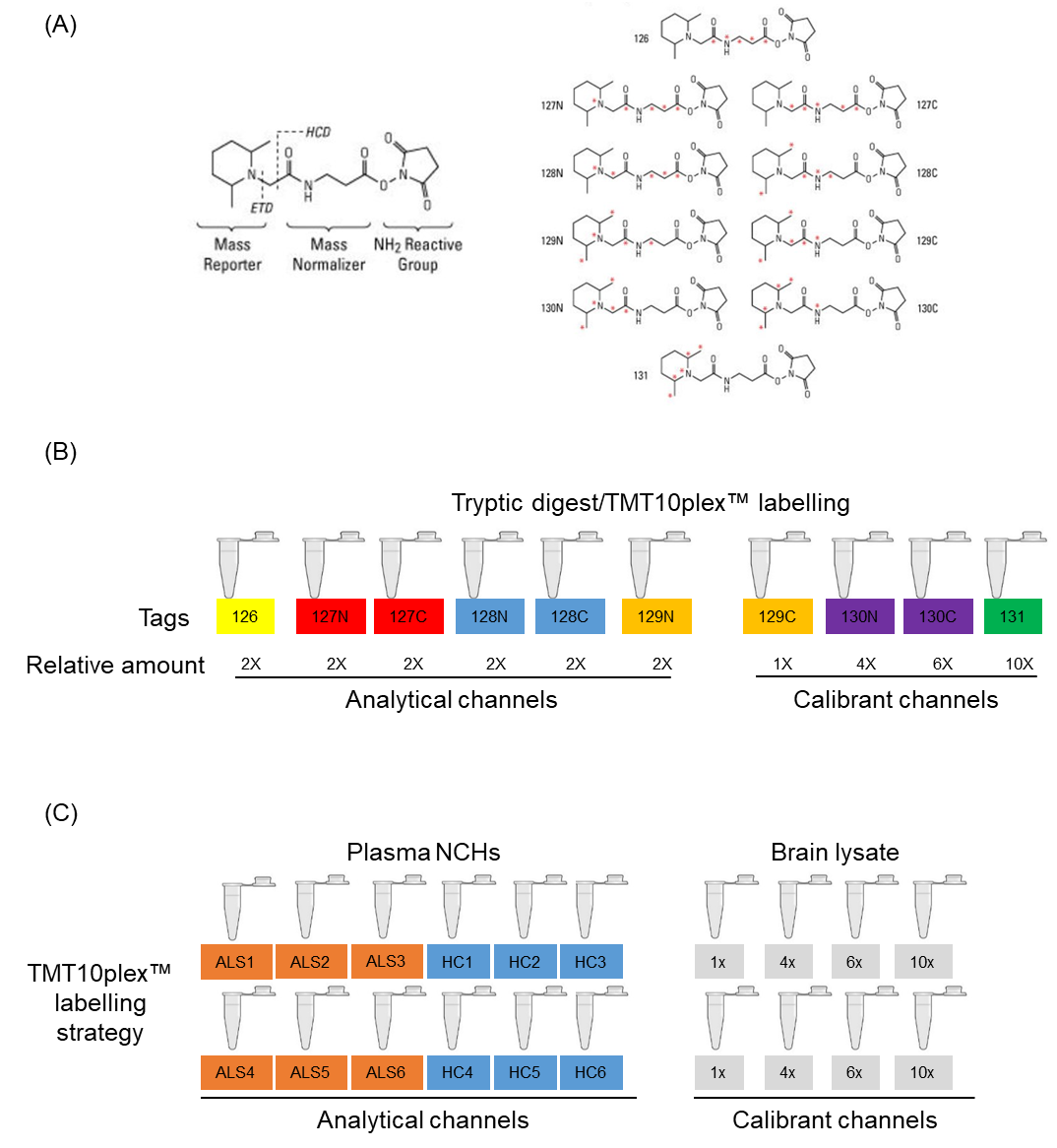


Supplementary Figure 1. TMTcalibrator™ experimental design.

(A) Tandem Mass Tag (TMT) reagents with relative masses and isotope position. (B) General 10plex labelling layout after trypsin digestion of the samples; analytical samples and calilbrants are mixed in a specific ratio that enhances detection by LC-MS/MS of low abundant peptides in the analytical channels thanks to the high calibrant content. (C) Labelling strategy in two 10plexes LC-MS/MS runs which includes Circulating Protein aggregates (CPA) from amyotrophic lateral sclerosis (ALS) patients and from healthy controls (HC) in the analytical channels (orange and blue colour codes) and a mixture (1:1) of brains (Precentral gyrus) lysates from two different ALS patients in the calibrant channels (grey colour code).


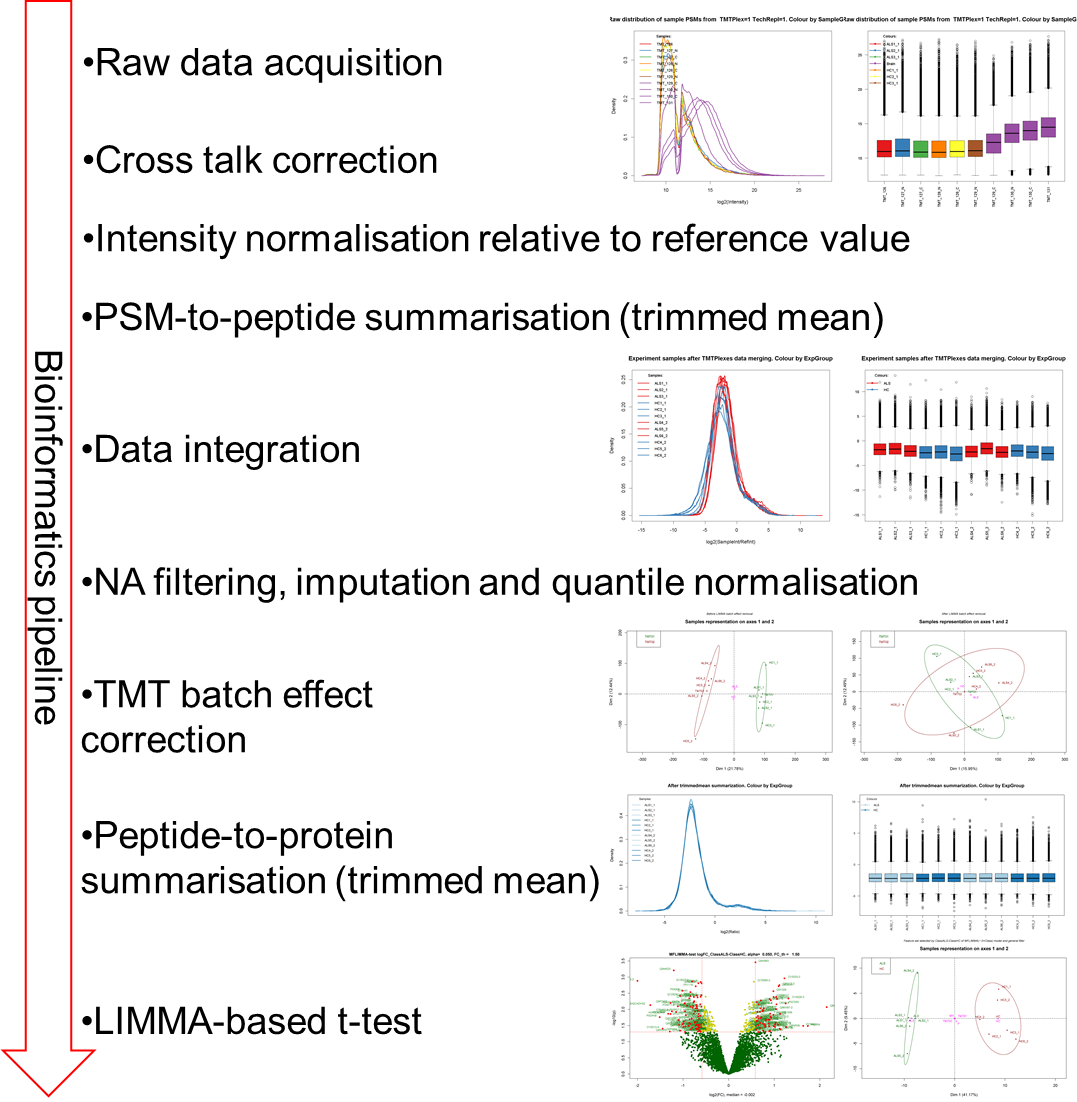


Supplementary Figure 2. TMTcalibrator™: bioinformatic pipeline.

After MS/MS spectra (raw data) acquisition, the intensity of each channel was corrected for background and cross-talking between tags in the second mass spectrometer (MS2). Intensity values of the detected Peptide-Spectrum Matches (PSMs) were normalised with a reference value generated as the average of the Calibrant channels and this was followed by PSM-to-peptide summarisation defined as “trimmed mean”. Data points considered as outliers in each analytical sample were removed stabilizing the mean before merging the data obtained from the two 10plexes. Then, “not available data points (NA)” filtering, imputation and quantile normalization were performed on the merged data set, so that it was possible to perform a Principal Component Analysis (PCA) on the data acquired. It was also possible to evaluate the TMT batch effect within linear models for microarray data (LIMMA). After peptide-to-protein summarisation, a statistically significant difference of expression (p-Value < 0.05, Fold Change threshold = 1.5) of the protein groups identified was tested using a LIMMA-based t-test .

**Supplementary figure – Circulating and Brain Protein Aggregates overlapping**

**
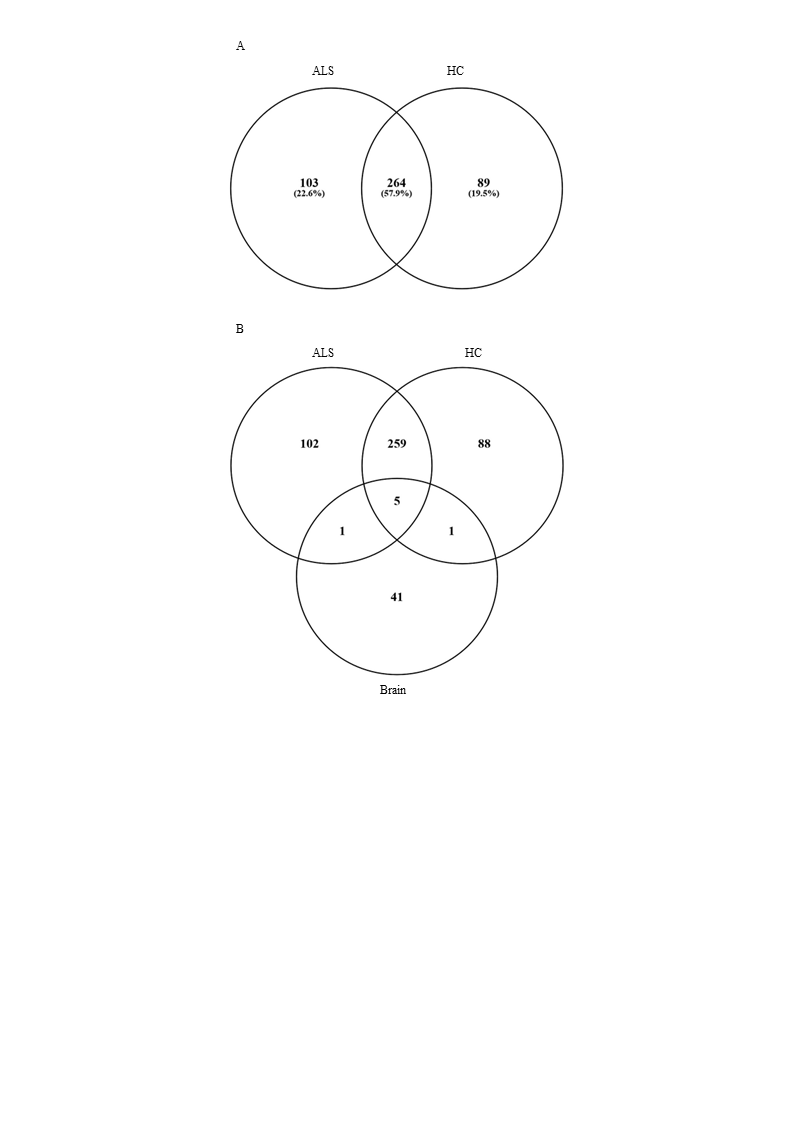
**

Supplementary Figure 3. Comparison of circulating protein aggregates (ALS and HC) and brain protein aggregates (ALS) composition.

(A) Venn diagram showing CPA proteins unique to or shared by ALS and HC. (B) Venn diagram showing HC and ALS CPA proteins shared by brain aggregates. Five proteins were expressed in all 3 aggregate groups (actin cytoplasmic 1, tubulin alpha-4A chain isoform 2, clathrin heavy chain 1 isoform 2, collagen alpha-1(VI) and plectin isoform 7), while brain aggregates shared only one protein with ALS and HC CPA (cytoplasmic dynein 1 heavy chain 1 and collagen alpha-2(VI), respectively).

**Supplementary figure – NfH in Circulating protein aggregates and proteases digestion**

**
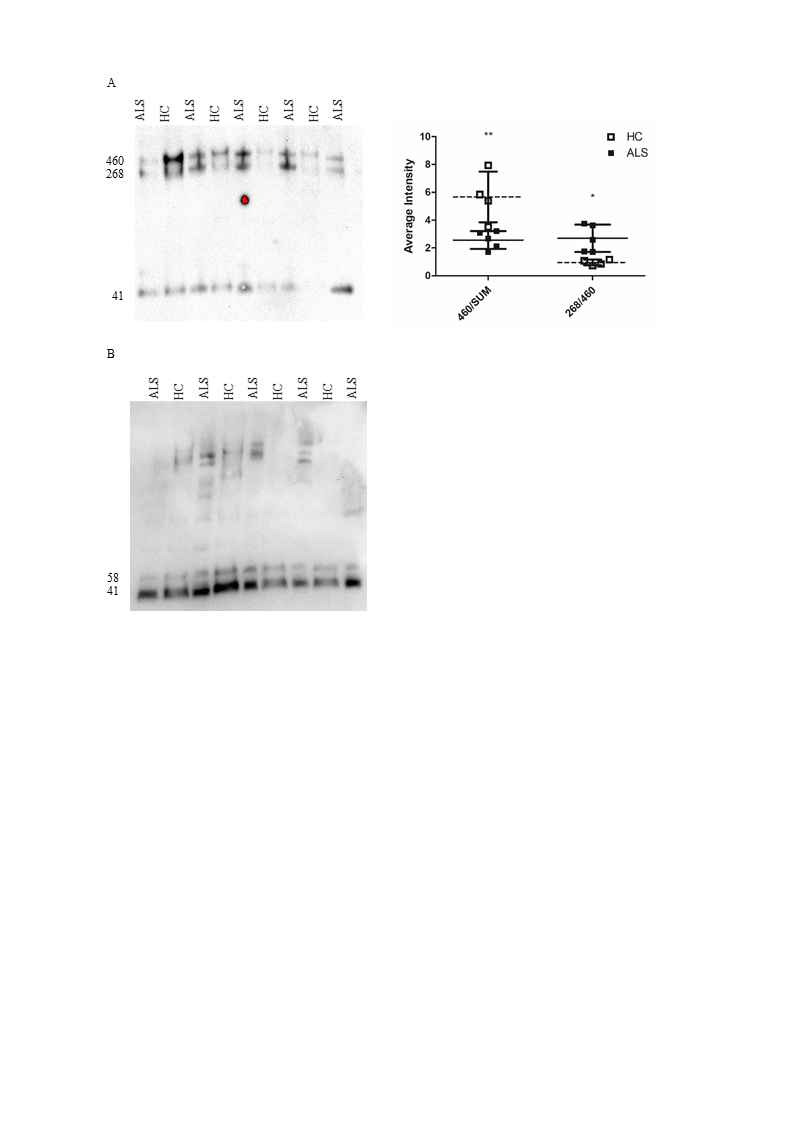
**

Supplementary Figure 4. Western blot analysis of neurofilament heavy chain (NfH) within circulating protein aggregates (CPA) and after Calpain digestion

Undigested CPA (A) show NfH bands at 460, 268 and 41 KDa (268 KDa is NfH expected molecular weight). The ratio between the 460 KDa band and the the sum of all NfH band intensities (SUM, 460/SUM) is higher in HC (p= 0.032), while the ratio between the 268 and 460 bands (268/460) is higher in ALS (p= 0.018). Calpain digestion (B) shows 58 and 41 KDa bands in all samples with no difference in expression. The enterokinase digestion profile of NfH

**Functional Analysis Tool (FAT) results: top10 regulated pathways in the TMTcalibrator™ experiment**

Supplementary Table 7. FAT analysis of the ALS vs HC TMT proteomics: top10 regulated pathways.

| Pathway description | Number of genes in the category | p-Value | Median logFC |
| --- | --- | --- | --- |
| Lipoprotein metabolism [Reactome] | 27 | 4.11e^-04^ | 0.19 |
| Dopamine Neurotransmitter Release Cycle [Reactome] | 15 | 4.53e^-04^ | 0.24 |
| DCC mediated attractive signaling [Reactome] | 8 | 6.65e^-04^ | 0.36 |
| Plasma lipoprotein assembly, remodeling, and clearance [Reactome] | 25 | 8.03e^-04^ | 0.19 |
| Plasma lipoprotein assembly [Reactome] | 13 | 1.65e^-03^ | 0.29 |
| Netrin-1 signaling [Reactome] | 15 | 4.80e^-03^ | 0.20 |
| Lipid digestion, mobilization, and transport [Reactome] | 36 | 5.25e^-03^ | 0.11 |
| Oncostatin M Signaling Pathway [Wikipathways] | 21 | 8.09e^-03^ | -0.13 |
| Plasma lipoprotein remodeling [Reactome] | 9 | 8.16e^-03^ | 0.19 |
| CD28 dependent Vav1 pathway [Reactome] | 6 | 8.35e^-03^ | 0.28 |

Pathway description: name of the pathway identified (reference database in square brackets, e.g. [Reactome])

Number of genes in the category: genes included in the given pathway in Homo sapiens

p-Value: statistical significance calculated by Mann-Whitney U test

Median logFC: median value of expression (logFC values) of the proteins included in the given pathway in Homo sapiens

Highly regulated proteins in the TMTcalibrator™ dataset

Supplementary Table 8. List of the proteins identified in the TMTcalibrator™ dataset with unique peptides ≥ 2, logarithmic fold change (LogFC) < -0.693 or > 0.693 and statistically significant (p-value < 0.05).

| **Uniprot ID** | **Gene name** | **Protein name** | **Unique peptides** | **logFC** | **p-value** |
| --- | --- | --- | --- | --- | --- |
| Q9P2W3 | GNG13 | Guanine nucleotide-binding protein G(I)/G(S)/G(O) subunit gamma-13 | 2 | -1.276 | 6.32e^-03^ |
| A0A0C4DH67 | IGKV1-8 | Immunoglobulin kappa variable 1-8 | 2 | -1.273 | 1.39e^-02^ |
| O75157-2 | TSC22D2 | Isoform 2 of TSC22 domain family protein 2 | 2 | -1.245 | 1.15e^-02^ |
| Q9NRZ5 | AGPAT4 | 1-acyl-sn-glycerol-3-phosphate acyltransferase delta | 2 | -1.205 | 6.21e^-04^ |
| P35579 | MYH9 | Myosin-9 | 82 | -1.000 | 2.70e^-02^ |
| Q9Y3E2 | BOLA1 | BolA-like protein 1 | 2 | -0.994 | 3.48e^-02^ |
| Q5QJ74 | TBCEL | Tubulin-specific chaperone cofactor E-like protein | 2 | -0.941 | 1.50e^-02^ |
| Q53GQ0 | HSD17B12 | Very-long-chain 3-oxoacyl-CoA reductase | 5 | -0.910 | 7.70e^-03^ |
| Q9UHI5 | SLC7A8 | Large neutral amino acids transporter small subunit 2 | 2 | -0.867 | 2.62e^-02^ |
| Q16718 | NDUFA5 | NADH dehydrogenase [ubiquinone] 1 alpha subcomplex subunit 5 | 2 | -0.862 | 4.52e^-03^ |
| Q9BU02 | THTPA | Thiamine-triphosphatase | 2 | -0.838 | 3.92e^-03^ |
| O75487 | GPC4 | Glypican-4 | 2 | -0.834 | 4.10e^-02^ |
| O15084 | ANKRD28 | Serine/threonine-protein phosphatase 6 regulatory ankyrin repeat subunit A | 3 | -0.809 | 1.92e^-02^ |
| P29144 | TPP2 | Tripeptidyl-peptidase 2 | 37 | -0.789 | 3.62e^-02^ |
| A8MWD9 | SNRPGP15 | Putative small nuclear ribonucleoprotein G-like protein 15 | 3 | -0.768 | 1.36e^-02^ |
| Q9NX63 | CHCHD3 | MICOS complex subunit MIC19 | 10 | -0.763 | 4.14e^-03^ |
| Q53H82 | LACTB2 | Endoribonuclease LACTB2 | 2 | -0.756 | 5.56e^-03^ |
| P50238 | CRIP1 | Cysteine-rich protein 1 | 3 | -0.742 | 2.61e^-02^ |
| P00813 | ADA | Adenosine deaminase | 3 | -0.733 | 1.16e^-02^ |
| P51116 | FXR2 | Fragile X mental retardation syndrome-related protein 2 | 3 | -0.723 | 1.93e^-03^ |
| P40855 | PEX19 | Peroxisomal biogenesis factor 19 | 2 | -0.722 | 9.35e^-03^ |
| P38919 | EIF4A3 | Eukaryotic initiation factor 4A-III | 6 | -0.709 | 7.35e^-03^ |
| Q9BYH1-3 | SEZ6L | Isoform 2 of Seizure 6-like protein | 4 | -0.697 | 8.24e^-03^ |
| Q9BV20 | MRI1 | Methylthioribose-1-phosphate isomerase | 4 | -0.692 | 3.74e^-03^ |
| P52788 | SMS | Spermine synthase | 5 | -0.689 | 2.44e^-02^ |
| Q99470 | SDF2 | Stromal cell-derived factor 2 | 2 | -0.682 | 4.26e^-03^ |
| Q9NPB8 | GPCPD1 | Glycerophosphocholine phosphodiesterase GPCPD1 | 2 | 0.683 | 1.19e^-02^ |
| O14514 | BAI1 | Brain-specific angiogenesis inhibitor 1 | 2 | 0.697 | 9.31e^-03^ |
| Q6NXE6-2 | ARMC6 | Isoform 2 of Armadillo repeat-containing protein 6 | 2 | 0.700 | 1.87e^-02^ |
| Q99729-3 | HNRNPAB | Isoform 3 of Heterogeneous nuclear ribonucleoprotein A/B | 3 | 0.706 | 5.29e^-03^ |
| Q9HD89 | RETN | Resistin | 4 | 0.754 | 2.78e^-02^ |
| P55083 | MFAP4 | Microfibril-associated glycoprotein 4 | 2 | 0.761 | 1.09e^-02^ |
| Q06828 | FMOD | Fibromodulin | 3 | 0.770 | 9.28e^-03^ |
| P45984-3 | MAPK9 | Isoform Beta-1 of Mitogen-activated protein kinase 9 | 2 | 0.789 | 2.87e^-02^ |
| Q9Y3C8 | UFC1 | Ubiquitin-fold modifier-conjugating enzyme 1 | 2 | 0.803 | 3.52e^-02^ |
| Q9NRA0-2 | SPHK2 | Isoform 2 of Sphingosine kinase 2 | 2 | 0.897 | 3.39e^-02^ |
| P04275 | VWF | von Willebrand factor | 127 | 0.910 | 4.69e^-02^ |
| P16519-2 | PCSK2 | Isoform 2 of Neuroendocrine convertase 2 | 3 | 0.911 | 1.35e^-02^ |
| P35219 | CA8 | Carbonic anhydrase-related protein | 2 | 0.925 | 5.23e^-03^ |
| Q9HAU0-2 | PLEKHA5 | Isoform 2 of Pleckstrin homology domain-containing family A member 5 | 2 | 0.933 | 1.84e^-02^ |
| Q9Y328 | NSG2 | Neuron-specific protein family member 2 | 2 | 0.981 | 2.73e^-03^ |
| Q00537 | CDK17 | Cyclin-dependent kinase 17 | 2 | 1.043 | 4.11e^-03^ |
| O60814 | HIST1H2BK | Histone H2B type 1-K | 2 | 1.049 | 6.97e^-03^ |
| P49406 | MRPL19 | 39S ribosomal protein L19, mitochondrial | 2 | 1.062 | 4.76e^-03^ |
| Q8IX12-2 | CCAR1 | Isoform 2 of Cell division cycle and apoptosis regulator protein 1 | 2 | 1.113 | 1.77e^-03^ |
| Q96F86 | EDC3 | Enhancer of mRNA-decapping protein 3 | 2 | 1.134 | 4.15e^-02^ |
| O15533-2 | TAPBP | Isoform 2 of Tapasin | 2 | 1.224 | 1.09e^-03^ |
| P27449 | ATP6V0C | V-type proton ATPase 16 kDa proteolipid subunit | 2 | 1.635 | 3.23e^-02^ |

Uniprot ID: Uniprot database protein identifier

Gene name: the recommended gene symbol used to officially represent a gene

Protein name: protein full name recommended by Uniprot

Unique peptides: number of peptide sequences unique to a protein group

logFC: relative quantification with value expressed as log2(ALS/HC) intensities

p-value: statistical significance of the differential regulation between ALS and HC experimental groups

Supplementary figure – Hierarchical clustering of regulated features in TMTcalibrator™


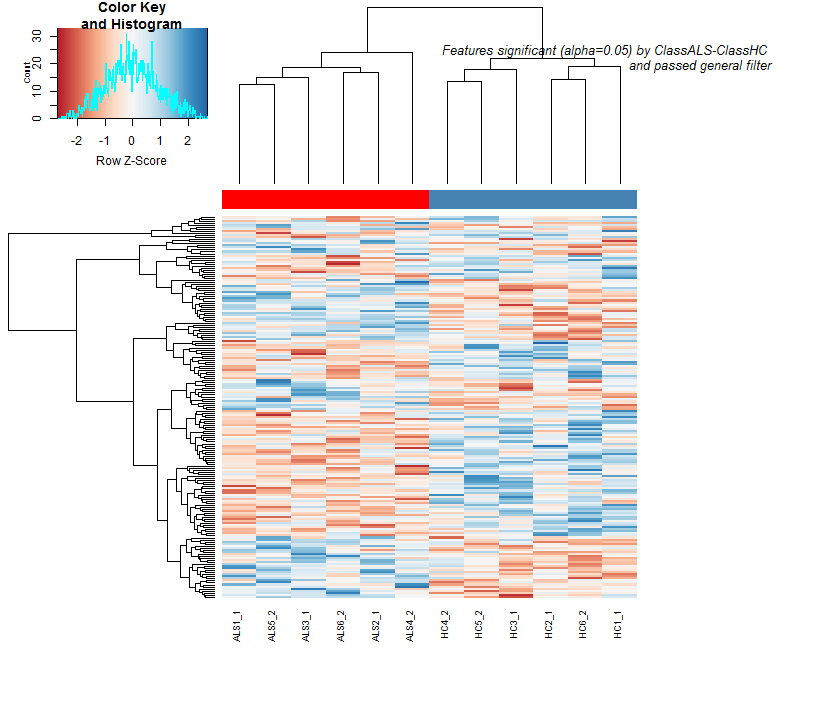


Supplementary Figure 5. Heatmap and hierarchical clustering.

Heatmap showing the distribution of the regulated features and their clustering. Regulated features are distributed vertically with relative clustering on the left-hand side. Analytical samples are distributed horizontally and relative clustering at the top of the heatmaps, with each sample highlighted at the bottom. In line with principal component analysis (Figure 4A), the heatmap groups ALS and HC samples separately. The color key histogram at the top left side shows the distribution of the features and the heatmap color coding. The list of regulated features is included in the final “Regulated proteins”, as well as on ProteomeXchange Consortium via the PRIDE repository.

Supplementary figure – Analysis of regulated proteins by immunodetection

**
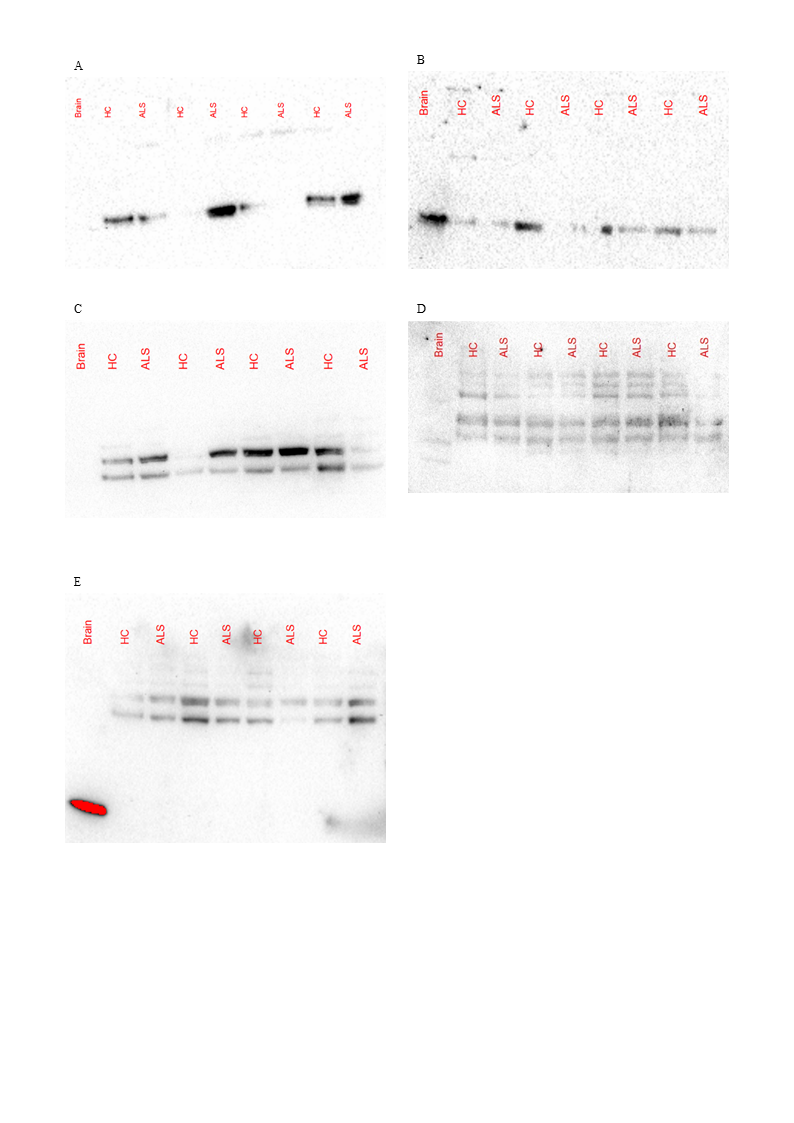
**

Supplementary Figure 6. Western blot analysis of five regulated proteins in plasma CPAs from ALS patients and healthy controls.

CPA samples from ALS (n=4) and HC (n=4), together with brain lysate, were tested for Glypican-4 (GPC4; A), Fibromodulin (FMOD; B), Biglycan (BGN; C), Cation-Dependent Mannose-6-Phosphate Receptor (M6PR; D) and Protein DJ-1 (PARK7; E). The targets tested showed a different trend from what obtained by TMTcalibrator™ analysis. In particular, BGN (C), M6PR (D) and PARK7 (E) showed multiple bands, which in some cases differed also from the brain lysate reference in terms of molecular weight (D and E). As stated in the main manuscript, no loading control was included for lack of constitutively expressed proteins in CPAs, as well as differential regulation presented in ALS literature for those proteins normally used in plasma and serum western blotting (e.g. albumin, transferrin, etc.).

**Supplementary figures – Full size and uncropped blots**

**
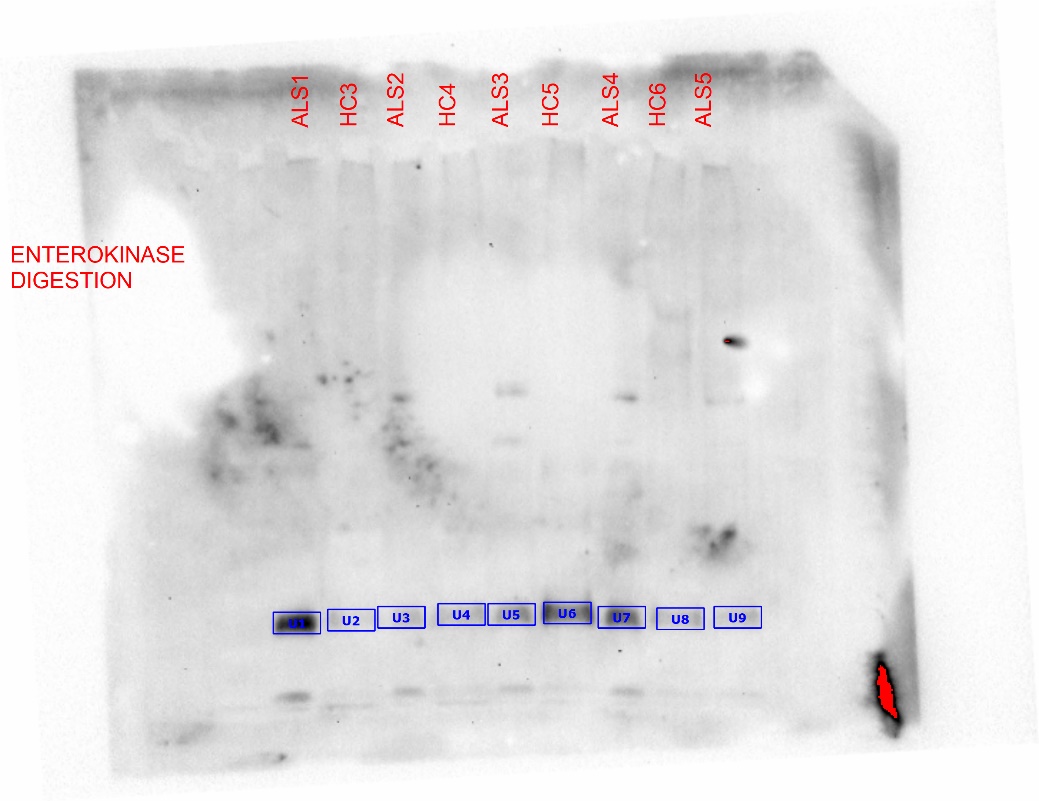
**

**Full size of Figure 3A. NfH in CPA after enterokinase digestion**

**The figure shows the entire blot after acquisition, including labels for samples and membrane. The blue frames were used to obtain data for semi quantification in Image Lab software (Bio-Rad), “Volume Tools” function.**

**
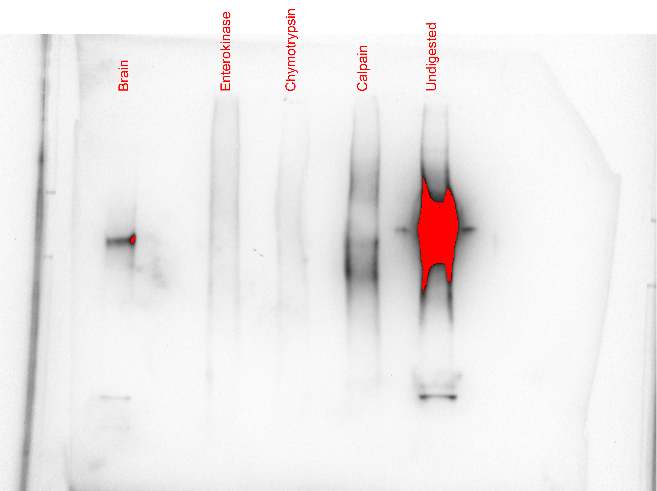

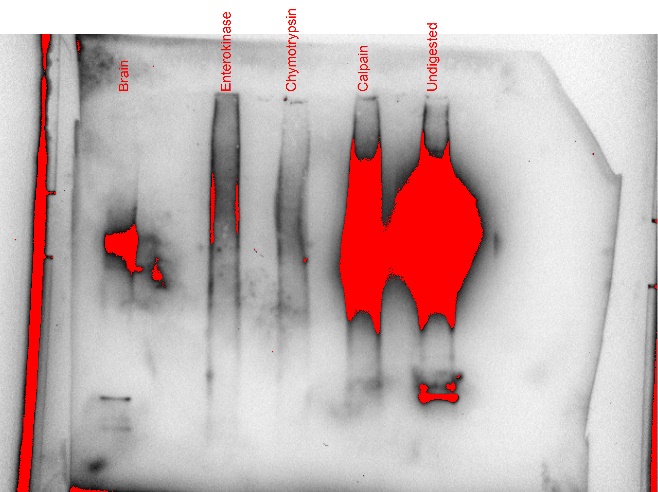

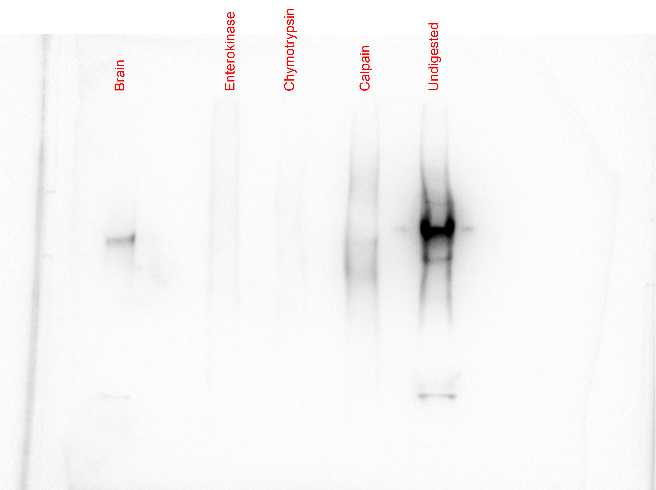
**

**Uncropped blots of Figure 3B. NfH in BPA after digestion with proteases and Brain**

**The figure shows the entire membrane at different exposure time to allow detection and determination of NfH isoforms and fragments in the samples. The images were acquired with Image Lab (Bio-Rad) and the red indicates saturated pixels.**

**
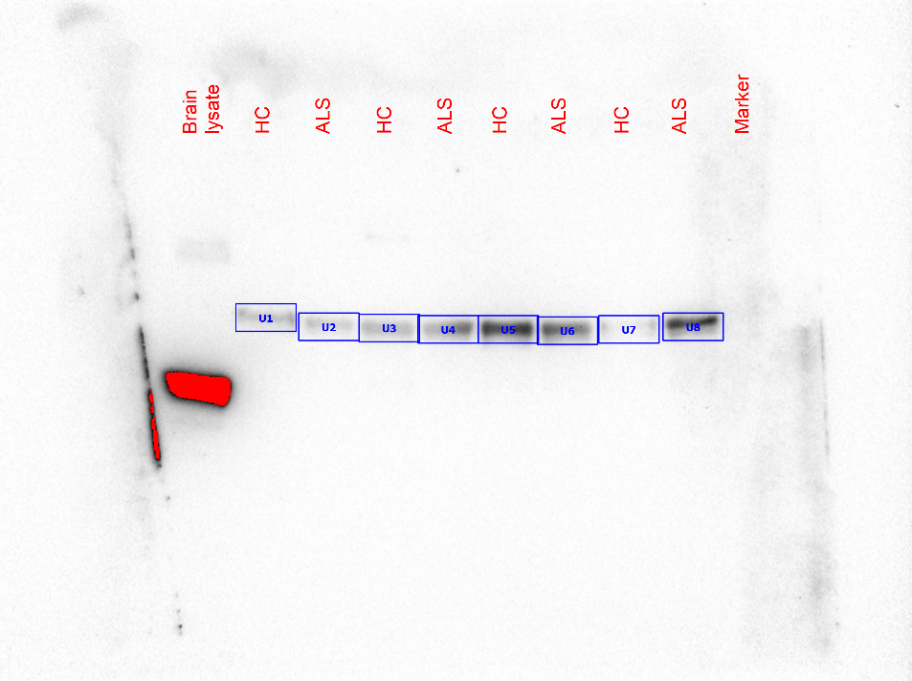
**

**Full size of Figure 5. Endophilin-B2 (SH3GLB2) in CPA**

**The figure shows the entire blot after acquisition, including labels for the samples. The blue frames were used to obtain data for semi quantification in Image Lab software (Bio-Rad), “Volume Tools” function.**

**
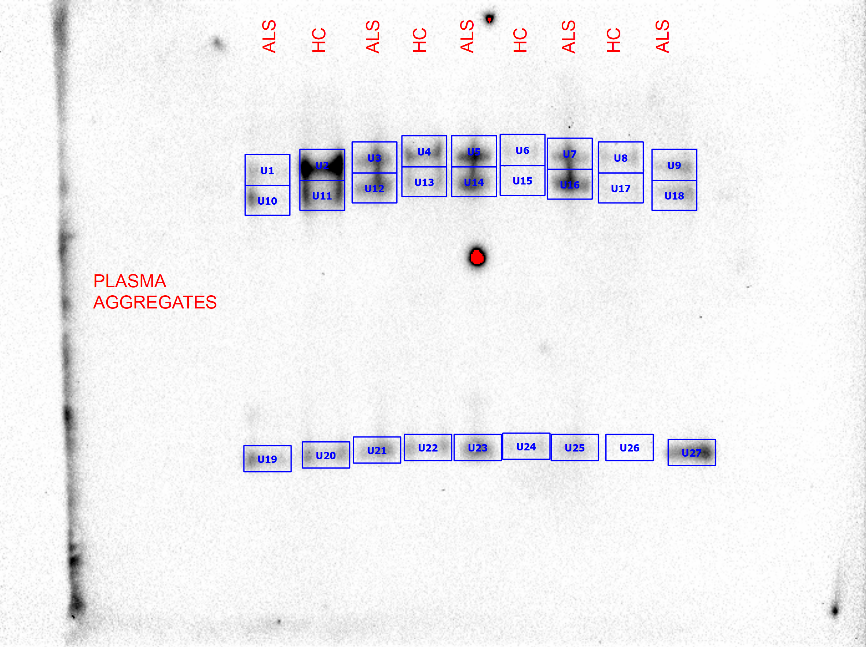
**

**Full size of Supplementary Figure 4A. NfH in CPA**

**The figure shows the entire blot after acquisition, including labels for samples and membrane. The blue frames were used to obtain data for semi quantification in Image Lab software (Bio-Rad), “Volume Tools” function.**

**
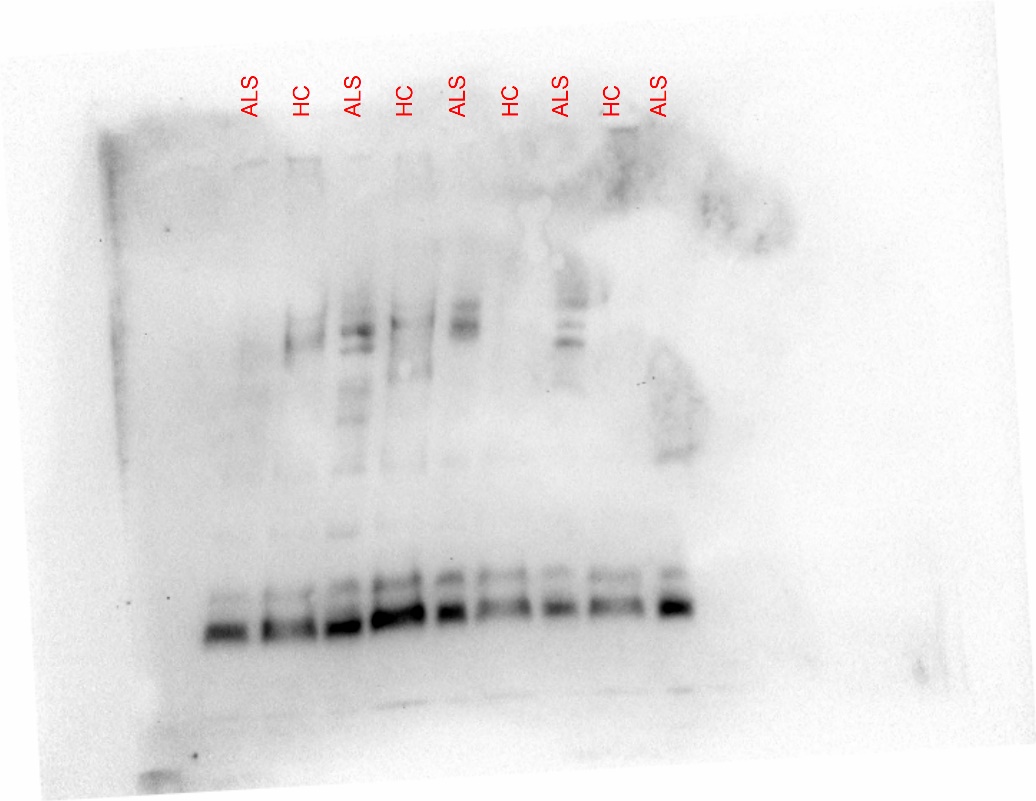
**

**Full size of Supplementary Figure 4B. NfH in CPA after Calpain digestion**

**The figure shows the entire blot after acquisition, including labels for samples, with Image Lab software (Bio-Rad).**

**SI References**

1. Ludolph A, Drory V, Hardiman O, et al. A revision of the El Escorial criteria - 2015. *Amyotroph Lateral Scler Frontotemporal Degener*. 2015;16(5-6):291-292. doi:10.3109/21678421.2015.1049183
