# Supplementary material for: Analysis of circulating protein aggregates as a route of investigation into neurodegenerative disorders
Source: Brain Commun. 2021 Jul 9;3(3):fcab148. doi: 10.1093/braincomms/fcab148 (PMC8361415; doi:10.1093/braincomms/fcab148)
Supplement: fcab148_Supplementary_Data [file Original Submission-Decision Letter .pdf › Original Submission-Decision Letter .pdf]

Original Submission-Decision Letter

Dear Dr. Malaspina,

Thank you for your recent submission BRAINCOM-2020-313 entitled "Analysis of circulating protein aggregates reveals pathological hallmarks of amyotrophic lateral sclerosis" to Brain Communications.

Your manuscript has been reviewed, and while the reviewers and editorial team appreciate many aspects of the paper, they raise some concerns that you will need to address in order for the paper to be considered for publication.

If you can address these concerns, I invite you to submit a revised manuscript and a response to each of the reviewers' comments. The reviewer(s)' comments are included at the bottom of this message.

Also, for the data presented in figure 4 could you indicate where the same ALS and HC sample has been analysied. For the cell viability experiments please confirm that the control samples were treated with comparible levels of urea as appropriate.

In addition to the reviewers' comments, please note our editorial comments added below to comply with our requirements for presentation of scientific data at Brain Communications.

Please format your revised manuscript according to our Instructions to Authors https://academic.oup.com/braincomms/pages/General_Instructions – a summary of key points is attached to help with this.

To revise your manuscript, click the link below or log into https://mc.manuscriptcentral.com/braincom and enter your Author Centre, where you will find your manuscript title listed under "Manuscripts with Decisions." Under "Actions," click on "Create a Revision." Your manuscript number has been appended to denote a revision.

*** PLEASE NOTE: This is a two-step process. After clicking on the link, you will be directed to a webpage to confirm. ***

https://mc.manuscriptcentral.com/braincom?URL_MASK=0acd9d83c63e4dfbb0fc8c69e86d8116

Please upload a revised manuscript with changes tracked with track changes or in coloured text.

When submitting your revised manuscript, you will be asked to respond to the reviewer(s) comments. Please be as specific as possible in your response to all of the comments.

The editorial team at Brain Communications aims to increase transparency in scientific publishing and to demonstrate the value that peer review brings to the scientific process. Please therefore consider whether you are willing to have reviewer(s)' comments on your paper and your response published as supplementary data with the paper if it is accepted. This is entirely optional and will only be done if both the authors and reviewers agree. While submitting your revision there will be an opportunity to review or update the answer you gave previously, if necessary.

IMPORTANT: Your original files are available to you when you upload your revised manuscript. Please delete any redundant files before completing the submission.

Because we are trying to facilitate timely publication of manuscripts submitted to Brain Communications, we would appreciate your revised manuscript as soon as possible. If it is not possible for you to resubmit within 3 months, the option to submit a revision will expire, and we would then consider your revised paper as a new submission. However, if you need a little more time, please get in touch and we can extend this deadline for you.

Once again, thank you for submitting your manuscript to Brain Communications and I look forward to receiving your revision.

Yours sincerely,

Dr. Paul Skehel
Editor, Brain Communications

Reviewer(s)' Comments to Author:

Reviewer: 1 (Christopher Henstridge)

Comments to the Author
In this manuscript, Adiutori and colleagues have used proteomics to assess the composition of protein aggregates isolated from plasma and brains of ALS patients. These have been compared with plasma-derived aggregates from healthy controls. While the title provided excitement and promise of a useful and interesting study, the data and its presentation, sadly did not. There are a number of major issues with the study, which I will describe in a figure-by-figure basis:

Figure 1: Presentation of the TEM micrographs is very poor. Panel D is completely out of focus and panels A+B have 2 scale bars, which are formatted differently to the other panels. Without any arrows, it’s extremely difficult to know which structures and particles are being referred to in the text.
Figure 2: While it is useful to have a summary Venn diagram of the proteomics data, I’m not sure it warrants an entire stand-alone figure. Most importantly, none of the data associated with this figure is provided anywhere. We don’t know for example what the 103 ALS proteins are in panel A. All the proteomics data should be provided in a supplementary table at the very least and some proteins of interest highlighted and discussed.
Figure 3: These data are interesting and well presented. However, as readers we have no idea which proteins are being analysed here, due to the lack of proteomics data provision.
Figure 4: It is interesting to look at the digestion profile of the aggregates, and here the authors focus on NfH. However, the blots are of very poor quality. Without uniform development/exposure across the membrane it’s impossible to be confident of densitometric analyses of bands. Furthermore, there are no loading controls provided for these blots. This is essential, for any quantification of Western blots.
Figure 5: This would have been a very useful figure, nicely showing the proteomic data and the log-fold changes in protein expression. However, the figure is extremely small and impossible to view in the pdf. This is not a mistake of the authors, but possibly a journal handling issue.
Figure 6: It’s good to see that the authors try to replicate their proteomics findings with another technique. However, again the blot suffers from non-uniform development/exposure and a lack of loading control. Furthermore, in the legend the authors state “Immunodetection confirmed the SH3GLB2 higher level of expression…, but without being statistically significant…”. This statement is incorrect. The statistical test shows no difference; therefore, you cannot state that an increased expression in ALS was observed. Finally, in the manuscript text the authors state that 5 other proteins were tested by western blot and all of them showed a different change in expression compared to the proteomics data. None of these blots were shown. It also raises the question why there is such a striking discrepancy between the proteomics and the blots?
Figure 7: Here, the authors have tried to analyse a pathophysiological role for these aggregated proteins. However, it is difficult to explain why the only effect on endothelial cell viability is with ALS-derived CPAs at the lowest tested concentration.

General comments: The paper is also rather poorly written. It is difficult to follow the logic or the flow of the manuscript and there are many English and grammar mistakes and typos. There are two supplementary figures which are not referred to in the main text.

Reviewer: 2 (Pamela McCombe)

Comments to the Author
This is an interesting study of protein aggregates in ALS. The number of subjects is small, particularly from the brain. Although the authors talk about understanding heterogeneity of ALS, the numbers are too small for this. The study is very detailed and yields interesting results namely that brain and ALS related proteins are found in aggregates in ALS but not control. This is of interest because such proteins would not be detected by other assays.
The authors talk about the possibility that such aggregated in the brain could be the vehicle for prion like spread. This is very speculative and not addressed in the experimental work.
The idea that protein aggregation is greater in ode subjects is interesting, and presumably would play a role if the brain aggregates were responsible for prion like spread, but again this is not addressed in the experimental work. Possibly these concepts would be better in the discussion than the introduction.
The main work is the blood study and this should be highlighted.
There is a problem with the references- I found many refs that were cited in the text but not in the reference list- there could be others I did nit check

Lehallier et al - page 22
Garbuzove -Daveis page 33
Petzold page 22
Ngo and steyn page 21 etc

Editorial Office comments:

- Fig4a: Please provide details of the statistical analysis performed, the experimental unit (n= cells,

wells, plate downs, etc)) and the number of data-points for each group analysed in these plots, and if the number is <10 show the single data-points. Please note our policy on pseudoreplication, we do not accept analyses where individual cells are the experimental unit when there is likely biological variability is between animals, culture replicates, etc. Please see instructions for authors for more details. Fort his type of data, we require either that a mean/median be taken per animal/culture replicate followed by statistical analyses or that more sophisticated analyses are performed such as mixed effects modelling which can account for random variables.

-Please supply a graphical abstract. A graphical abstract is a visual representation of the central finding or methodology of the paper. It will be displayed in the table of contents. Authors should strive to make the graphical abstract informative, interesting, visually appealing, and straightforward (no caption needed, no abbreviations except gene names). Please upload as a separate high resolution image file (tiff preferred). The technical requirements for the graphical abstracts are:
--size of the submitted image: 1200 pixels square at 300dpi
--font of the text: Arial 12-16 point (smaller fonts will not be legible online)

-Please add an Abbreviated Summary (up to 50 words) of your paper that captures the main purpose and conclusions of the work. The summary should state the main results written in the third person ('[1st author surname] et al. report that …') and conclusion. This summary will be used in the contents list online and should not contain any abbreviations other than those on the accepted abbreviations list and accepted gene/protein names, making it accessible to the non-specialist. Please upload it as a Word document. Allowed abbreviations: AIDS; ANOVA; AMPA; ATP; A,T,C,G; CNS; CSF; CT; DNA; ECG; EEG; EMG; GABA; HIV; MRI; NMDA; PET; PCR; RNA.

-Please include a paragraph about data availability in the 'Materials and Methods' section. We strongly encourage data sharing either as supplementary information or uploads to repositories which you can link in the paper.

-Full- size and uncropped blots/gels are to be made available as Supplementary material.

-To comply with our ethical standards, the appropriate checklist must be included (see Reporting Guideline section within 'Instructions to authors').
