# Supplementary material for: Analysis of circulating protein aggregates as a route of investigation into neurodegenerative disorders
Source: Brain Commun. 2021 Jul 9;3(3):fcab148. doi: 10.1093/braincomms/fcab148 (PMC8361415; doi:10.1093/braincomms/fcab148)
Supplement: fcab148_Supplementary_Data [file Revision 1- Author’s Response.pdf › Revision 1- Author’s Response.pdf]

Revision 1- Author's Response

Dear Editor,
thanks for highlighting the presence of a duplicate image and the opportunity to address the minor comments from the Reviewers. We report below a response to each point raised by the Reviewers.

• Reviewer: 1
Figure 1 looks much better in my opinion, with the new panel D and the inclusion of arrows. However, the scalebars are still distractingly heterogenous. I feel this is an editorial consideration, so merely highlight it here.
After acquisition, images were adjusted with ImageJ and to include scalebars of similar sizes (500 nm for A and B; 50 nm from C to F). However, the scalebars are heterogenous in size because not all the micrographs have been taken at same magnification, for this reason, we have not been able to generate images with the same scalebar sizes. We agree nevertheless with the Reviewer, these differences might distract the reader and therefore, we have adjusted the legends size and font to make them more homogeneous.
Figure 2 - The authors should be commended for their addition of the proteomics file and the submission to PRIDE.
Many thanks for the nice words. We believe that science is above all about sharing data and information and we are committed to make all our data available to those working on the same research field.
Figure 3/4 - I agree with the authors that loading controls would be difficult, if not impossible to select, but a total protein stain (Ponceau for example) would convince readers that equal loading has been achieved between samples. I accept that the authors have explained the correction factors and how they were generated to suggest equal loading. Again, I feel this may be an editorial consideration regarding the visualisation and quantification of western blots.
We are grateful for the understanding. We carried out Ponceau staining in all the western blots as standard practice to verify protein transfer from gel to membrane and even-loading. However, we did not take a picture of the staining for all the blots performed, having decided to include another robust internal control for loading as described in the methods.
Figure 6 – I accept that Westerns are not as sensitive as proteomics analyses. It is good that the authors now include the blots in supplementary data.
Thanks for the understanding.
Please note, the full size blots on page 59 and 61 are duplicates, yet claim to be different experiments.
We apologize for the mistake and we have replaced the Full size of Supplementary Figure 4B ("NfH in CPA after Calpain digestion") with the correct image.
The new added text in the discussion helps clarify some points for the reader.
We appreciate for the suggestions made in the first instance.
• Reviewer: 2
The authors have revised the manuscript and I have no further comments
• Editorial Office comments:
please, update the legends associated to the uncropped blots according to the figure organisation of the revised manuscript.
The Uncropped Blots section in the Supplementary Information file have been adjusted following the figure organisation in the revised manuscript. We included the blots from the manuscript (Full size of Figure 3A; Uncropped blots of Figure 3B; Full size of Figure 5) followed by those presented in the Supplementary Information (Full size of Supplementary Figure 4A; Full size of Supplementary Figure 4B).
