# Supplementary material for: Analysis of circulating protein aggregates as a route of investigation into neurodegenerative disorders
Source: Brain Commun. 2021 Jul 9;3(3):fcab148. doi: 10.1093/braincomms/fcab148 (PMC8361415; doi:10.1093/braincomms/fcab148)
Supplement: fcab148_Supplementary_Data [file Revision 1- Decision Letter.pdf › Revision 1- Decision Letter.pdf]

Revision 1- Decision Letter

17-Feb-2021

Dear Dr. Malaspina,

Thank you for your recent submission BRAINCOM-2020-313.R1
entitled "Analysis of circulating protein aggregates as a route of investigation into neurodegenerative disorders" to Brain Communications.

Your manuscript has been re-reviewed, and while the reviewers had largely positive comments some minor revision of the manuscript is still required. The reviewer(s)' comments are included at the bottom of this message. The issue we would particularly like you to address concerns supplementary figures 3A and 4B, showing immunoblot analysis following enterokinase and calpain digestions respectively. As stated, these are separate experiments, but the identical image is presented in both figures.

In addition to the reviewers' comments, please note our editorial comments added below to comply with our requirements for presentation of scientific data at Brain Communications.

Please format your revised manuscript according to our Instructions to Authors https://academic.oup.com/braincomms/pages/General_Instructions – a summary of key points is attached to help with this.

To revise your manuscript, click the link below or log into https://mc.manuscriptcentral.com/braincom and enter your Author Centre, where you will find your manuscript title listed under "Manuscripts with Decisions." Under "Actions," click on "Create a Revision." Your manuscript number has been appended to denote a revision.

*** PLEASE NOTE: This is a two-step process. After clicking on the link, you will be directed to a webpage to confirm. ***

https://mc.manuscriptcentral.com/braincom?URL_MASK=658559a8a1b44c8e9d7687048be119dc

Please upload a revised manuscript with changes tracked using track changes or in coloured text.

When submitting your revised manuscript, you will be asked to respond to the reviewer(s) comments. Please be as specific as possible in your response to all of the comments.

The editorial team at Brain Communications also aims to increase transparency in scientific publishing and to demonstrate the value that peer review brings to the scientific process. Please therefore consider whether you are willing to have reviewer comments on your paper and your response published as supplementary data with the paper if it is accepted. This is entirely optional and will only be done if both the authors and reviewers agree. When submitting your revision you will have the opportunity to update the answer you entered previously.

IMPORTANT: Your original files are available to you when you upload your revised manuscript. Please delete any redundant files before completing the submission.

Because we are trying to facilitate timely publication of manuscripts submitted to Brain Communications, we would appreciate your revised manuscript as soon as possible. If it is not possible for you to resubmit within 3 months, the option to submit a revision will expire, and we would then consider your revised paper as a new submission. However, if you need a little more time, please get in touch and we can extend this deadline for you.

Once again, thank you for submitting your manuscript to Brain Communications and I look forward to receiving your revision.

Yours sincerely,

Dr. Paul Skehel
Editor, Brain Communications

Reviewer(s)' Comments to Author:

Reviewer: 2 (Pamela McCombe)

Comments to the Author
The authors have revised the manuscript and I have no further comments

Reviewer: 1 (Christopher Henstridge)

Comments to the Author
In this rebuttal, Adiutori and colleagues have made changes to their initial submission, which I feel have strengthened the manuscript. However, there are still some points that I feel need addressed.
Figure 1 looks much better in my opinion, with the new panel D and the inclusion of arrows. However, the scalebars are still distractingly heterogenous. I feel this is an editorial consideration, so merely highlight it here.
Figure 2 - The authors should be commended for their addition of the proteomics file and the submission to PRIDE.
Figure 3/4 - I agree with the authors that loading controls would be difficult, if not impossible to select, but a total protein stain (Ponceau for example) would convince readers that equal loading has been achieved between samples. I accept that the authors have explained the correction factors and how they were generated to suggest equal loading.
Again, I feel this may be an editorial consideration regarding the visualisation and quantification of western blots.
Figure 6 – I accept that Westerns are not as sensitive as proteomics analyses. It is good that the authors now include the blots in supplementary data.
Please note, the full size blots on page 59 and 61 are duplicates, yet claim to be different experiments.
The new added text in the discussion helps clarify some points for the reader.

Editorial Office comments:

- please, update the legends associated to the uncropped blots according to the figure organisation of the revised manuscript.
