# Supplementary material for: Analysis of circulating protein aggregates as a route of investigation into neurodegenerative disorders
Source: Brain Commun. 2021 Jul 9;3(3):fcab148. doi: 10.1093/braincomms/fcab148 (PMC8361415; doi:10.1093/braincomms/fcab148)
Supplement: fcab148_Supplementary_Data [file Revision 2- Decision Letter.pdf › Revision 2- Decision Letter.pdf]

Revision 2- Decision Letter

Dear Dr. Malaspina,

It is a pleasure to accept your manuscript entitled "Analysis of circulating protein aggregates as a route of investigation into neurodegenerative disorders" for publication in Brain Communications. Any comments made by the reviewer(s) are included below.

The next step is for your paper to go through “First Look”. We will contact you again shortly if any final files or formatting changes are required for publication or if we have any queries.

If no changes are needed, your submission will be transferred to our Production Department and you will hear from the Production Editor in due course.

Video abstracts - Brain Communications encourages authors to provide a video abstract to accompany their article. These are hosted on the Brain/Brain Communications YouTube channel. If you are interested in producing a 5-min video abstract to promote your article, then please contact the Editorial Office (brcoms.editorialoffice@oup.com) for further details and guidelines.

Thank you very much for your contribution. On behalf of the Editors of Brain Communications, we look forward to your future contributions to the Journal.

Yours sincerely,

Reviewer(s)' Comments to Author:

Reviewer: 1 (Christopher Henstridge)

Comments to the Author
Figure 1 looks much tidier and my issue with the supplementary data has been addressed.

Reviewer: 2 (Pamela McCombe)

Comments to the Author
The changes have enhanced the manuscript
