# Supplementary material for: Analysis of circulating protein aggregates as a route of investigation into neurodegenerative disorders
Source: Brain Commun. 2021 Jul 9;3(3):fcab148. doi: 10.1093/braincomms/fcab148 (PMC8361415; doi:10.1093/braincomms/fcab148)
Supplement: fcab148_Supplementary_Data [file Original Submission - Author's Response.pdf › Original Submission - Author's Response.pdf]

Dear Editor, many thanks for the opportunity to address the Reviewers comments. We report below point-by-point responses to each point raised by the Reviewers. Our manuscript has been extensively revised as requested and all changes in response to criticisms highlighted in yellow in the text.

- **Reviewer 1**

In this manuscript, Adiutori and colleagues have used proteomics to assess the composition of protein aggregates isolated from plasma and brains of ALS patients. These have been compared with plasma-derived aggregates from healthy controls. While the title provided excitement and promise of a useful and interesting study, the data and its presentation, sadly did not. There are a number of major issues with the study, which I will describe in a figure-by-figure basis:

Figure 1: Presentation of the TEM micrographs is very poor. Panel D is completely out of focus and panels A+B have 2 scale bars, which are formatted differently to the other panels. Without any arrows, it's extremely difficult to know which structures and particles are being referred to in the text.

We have now reconfigured the TEM micrographs. Panel D (a magnification of a detail from a micrograph) has now been replaced with another picture. The same scale bar format is now used for all panels and arrows have been added to indicate details which are reported and discussed in the text.

Figure 2: While it is useful to have a summary Venn diagram of the proteomics data, I'm not sure it warrants an entire stand-alone figure. Most importantly, none of the data associated with this figure is provided anywhere. We don't know for example what the 103 ALS proteins are in panel A. All the proteomics data should be provided in a supplementary table at the very least and some proteins of interest highlighted and discussed.

We have now removed the Venn diagram and placed it in the Supplementary file (Supplementary figure 3). We now provide tables listing all the proteins identified in ALS and HC circulating aggregates and those that are shared by these blood formations and with brain aggregates. We make specific reference to and discuss in the text key proteins shared by brain and blood aggregates as well as those represented in aggregates from ALS, including proteins like tubulin alpha-4A chain isoform which are critical in the maintenance of axonal function patients.

Figure 3: These data are interesting and well presented. However, as readers we have no idea which proteins are being analysed here, due to the lack of proteomics data provision.

The Reviewer is right in mentioning that reference to the exact group of proteins reported here has to be clearer. To this end, an excel file has been added to the submission with all the proteins identified and used to generate the analysis shown in what is now Figure 2 (replacing former Figure 3. Each worksheet contains the Uniprot IDs, with relative description for the protein subsets mentioned in the main text and described in Figure 2 (Unique ALS, Unique Control, Shared, Brain).

In addition, the data obtained using the proteomic approach detailed in our paper have been submitted to PRIDE under two main sections: 1) data obtained from plasma pooled plasma samples and brain protein aggregates (PRIDE identifier PXD018938), 2) data from the

TMTcalibrator proteomic output (PRIDE identifier PXD018923). The reviewer can access this material with the following credentials:
- dataset PXD018938: reviewer53086@ebi.ac.uk, Password: 08GOFSN7
- dataset PXD018923: reviewer_pxd018923@ebi.ac.uk, Password: oTqeyeqS

Figure 4: It is interesting to look at the digestion profile of the aggregates, and here the authors focus on NfH. However, the blots are of very poor quality. Without uniform development/exposure across the membrane it's impossible to be confident of densitometric analyses of bands. Furthermore, there are no loading controls provided for these blots. This is essential, for any quantification of Western blots.

The former Figure 4 is now Figure 3.

This experiment and its representation is necessarily limited by the loading of very low amounts of proteins in the form of protein aggregates extracted from plasma which require higher exposure for visualization. We definitely agree with the Reviewer that this has affected the quality of the pictures. We are also showing the entire membranes to include all different isoforms and fragments and not only a specific set of bands, to follow the Journal guidelines.

With regard to the reported lack of loading controls in our western blot analysis, as reported in the text and described in detail in the supplementary material ("Correction factors for semi-quantitative analysis of neurofilament heavy chain (NfH) in circulating protein aggregates (CPAs) before and after digestion with proteases"), we have indeed corrected for loading differences across samples by using the gel Markers and by calculating correction factors (CFs) using both size Markers and the intensity of bands from matched undigested samples. We believe that this was the most accurate way to balance the differences observed after SDS-PAGE and due uneven loading.

In our view, the study of protein aggregates coming from ALS using western blotting does not allow the use of loading controls that would be normally considered as "constitutively expressed" as we would not be able to rule out any regulation related to the disease process.

It would be in fact difficult to select proteins as loading controls among those normally used in western blotting and expect no change between the study groups. In fact, proteins used as loading controls in plasma and serum westerns blotting like albumin, transferrin, Beta Actin, Beta Tubulin, Cyclophilin and GAPDH have been reported to be regulated in in ALS (Andrés-Benito, Moreno, Aso, Povedano, & Ferrer, 2017; Tam et al., 2019; Wang, Simmons, Liu, Boyer, & Connor, 2006 included in manuscript's bibliography). Moreover, due to the lack of knowledge in circulating protein aggregates in blood, presence and/or composition of constitutively expressed proteins is unknown. To our knowledge, this is the first paper presenting data on circulating protein aggregates in blood obtained from individuals, healthy or affected by a neurodegenerative disease.

To address the Reviewer comment and since the information we would like to convey is the different pattern of enterokinase digestion in ALS compared to HC (with digestion fragments observed only in ALS) and the digestion pattern observed in aggregates from brain, we have simplified the figure leaving only f panel C and D in the main text. We have taken off and put in the supplementary material the remaining panels showing NfH in undigested CPA and after digestion with calpain.

Figure 5: This would have been a very useful figure, nicely showing the proteomic data and the log-fold changes in protein expression. However, the figure is extremely small and impossible to view in the pdf. This is not a mistake of the authors, but possibly a journal handling issue.

The former Figure 5 is now Figure 4.

We have now included a larger picture where details may be more visible. The size is compatible with what recommended by the Journal (18cm width). In case the Editor wanted a different graphical representation, we will be happy to provide each panel in the original size.

Figure 6: It's good to see that the authors try to replicate their proteomics findings with another technique. However, again the blot suffers from non-uniform development/exposure and a lack of loading control.
Furthermore, in the legend the authors state "Immunodetection confirmed the SH3GLB2 higher level of expression…, but without being statistically significant…". This statement is incorrect. The statistical test shows no difference; therefore, you cannot state that an increased expression in ALS was observed.
Finally, in the manuscript text the authors state that 5 other proteins were tested by western blot and all of them showed a different change in expression compared to the proteomics data. None of these blots were shown. It also raises the question why there is such a striking discrepancy between the proteomics and the blots?

The former Figure 6 is now Figure 5.

We refer to our answer to the previous question regarding the lack of loading controls in our western blotting experiments to look at patterns of protease digestion of aggregates. The small amount of protein aggregates loaded requires a long and non-uniform exposure (across different experiments) of the gels for visualization of the relevant bands. This is a methodological hurdle intrinsic to experiments that have to visualize very small quantities of protein material, as in protein aggregates from biological fluids.

We agree with the Reviewer about the lack of statistical significance and we apologise if this has been worded incorrectly. We report now clearly, as shown in the graph, that the higher levels in ALS do not reach statistical significance in the comparison with HC.

The 5 other proteins selected for western blotting re-testing were analysed and did not reproduce the direction and the level of differential regulation identified in the Proteomic experiment. We have now added the western blot figures and results in the supplementary file. The Reviewer makes a valid point about the discrepancy between the Proteomic and western blotting results. We have nevertheless mentioned and extensively discussed in the text that we believe that western blot immunodetection would not necessarily reproduce the regulation of peptides seen using proteomics. The use of orthogonal techniques like immunoassays to reproduce and validate the regulation of peptides identified through large-scale proteomic studies has always had inherent limitations, which are amplified by the inclusion of protein aggregates rather than soluble proteins as targets. We have now reinforced this concept in "Analysis of regulated proteins by immunodetection" and in the "Conclusions". Reproduction of the differential regulation of proteins identified by proteomics in aggregate formations may not be possible using immunodetection techniques, since protein epitopes interaction with the immunoassay antibodies may be altered in a situation in which proteins are deeply embedded and assembled within aggregates, even after treatment with urea. In addition, the peptides identified in plasma which are expressed in the brain calibrant used in our proteomic experiments may have different post-translational modification, making any validation experiment using immunodetection in plasma to confirm proteomic data not viable.

Figure 7: Here, the authors have tried to analyse a pathophysiological role for these aggregated proteins. However, it is difficult to explain why the only effect on endothelial cell viability is with ALS-derived CPAs at the lowest tested concentration.

The former Figure 7 is now Figure 6.

This is a valid point from the Reviewer which we have discussed in the submitted manuscript and now in the revised paper. The higher impact (reduced cell viability) on cells treated with the lowest tested CPA concentration in ALS is in our view related to the potential higher toxicity that smaller, possibly oligomeric, protein assemblies may have on cells compared to the effect that larger aggregates likely to be found in higher concentration solutions may have.

Our observation is based on data in the literature on amyloid particles, where larger size assemblies have been found to have less detrimental effect than the smaller size counterparts, which may include more oligomeric type of aggregate formation (Xue et al., 2009 as reported in bibliography).

In low concentration solutions we are more likely to select for potential residual oligomeric formations from CPA. Larger aggregates in lower concentration solutions may exert a protective effect by sequestering potentially toxic proteins that may otherwise have a higher degree of contact with cell membranes in the cell viability experiments.

Nevertheless, our interpretation is clearly based on speculations and a better understanding, as we advocate in the paper, will depend on future studies on the composition and conformation of CPAs under pathological and healthy conditions.

General comments: The paper is also rather poorly written. It is difficult to follow the logic or the flow of the manuscript and there are many English and grammar mistakes and typos. There are two supplementary figures which are not referred to in the main text.

We have now rewritten the paper and had it fully reviewed by a Native Speaker. All the supplementary figures are mentioned in the text. The two supplementary figures are now mentioned in the materials and methods section. Supplementary Figure 1 is mentioned in the paragraph "TMTcalibrator™", while supplementary Figure 2 is mentioned in the "Bioinformatics" section. Supplementary figure 1 and 2 support the reader with a graphical reproduction of the TMTcalibrator experimental setting and the data analysis pipeline.

- **Reviewer 2**

This is an interesting study of protein aggregates in ALS. The number of subjects is small, particularly from the brain. Although the authors talk about understanding heterogeneity of ALS, the numbers are too small for this. The study is very detailed and yields interesting results namely that brain and ALS related proteins are found in aggregates in ALS but not control. This is of interest because such proteins would not be detected by other assays.
The authors talk about the possibility that such aggregated in the brain could be the vehicle for prion like spread. This is very speculative and not addressed in the experimental work.
The idea that protein aggregation is greater in ode subjects is interesting, and presumably would play a role if the brain aggregates were responsible for prion like spread, but again this is not addressed in the experimental work. Possibly these concepts would be better in the discussion than the introduction.
The main work is the blood study and this should be highlighted.

We thank the Reviewer for the appreciation of our work. We agree the study is conducted on a relatively small number of samples given the exploratory nature of the investigation and the labour-intensive methodologies employed for aggregates extraction. Therefore, we fully acknowledge that any reference to an association between proteomic data and clinical

phenotype of ALS would not be possible with the current cohort size. We have not intended to emphasize that our work would be important to unravel clinical heterogeneity in ALS. The aim of our work has been to explore a potential new route for the identification of protein biomarkers in circulating aggregates that may not be easily detectable and to demonstrate that these formations may contain brain-derived molecules that are relevant to the pathogenesis of a neurogenerative condition like ALS. We have now made more explicit that our work addresses simply the changes that come into place under ALS pathological conditions and the high density of brain peptides recoverable from circulating protein aggregates.

Likewise, we have mentioned that aggregation of proteins in biological fluid may be a physiological phenomenon which may be altered under pathological conditions and speculated that these changes systemically may contribute to the pathological process and disease progression by, for example, affecting blood brain barrier permeability. It is not our intention to suggest that any pathological effect that circulating protein aggregates may have could occur through a prion-like mechanism, as this would be highly speculative given the lack of any experimental work supporting this. We have modified the text to make sure that we put emphasis on the body of work on blood as suggested by the reviewer, in both introduction and in the conclusion.

There is a problem with the references- I found many refs that were cited in the text but not in the reference list- there could be others I did nit check

We have now thoroughly checked all references matching citations in the text.

- **Editor**

Fig4a: Please provide details of the statistical analysis performed, the experimental unit (n= cells, wells, plate downs, etc)) and the number of data-points for each group analysed in these plots, and if the number is <10 show the single data-points. Please note our policy on pseudoreplication, we do not accept analyses where individual cells are the experimental unit when there is likely biological variability is between animals, culture replicates, etc. Please see instructions for authors for more details. For this type of data, we require either that a mean/median be taken per animal/culture replicate followed by statistical analyses or that more sophisticated analyses are performed such as mixed effects modelling which can account for random variables

Former Figure 4a is not anymore in the text, we moved to supplementary information (Supplementary figure 4) and the plot has been changed from bar to points, hence showing each data point.

-Please supply a graphical abstract. A graphical abstract is a visual representation of the central finding or methodology of the paper. It will be displayed in the table of contents. Authors should strive to make the graphical abstract informative, interesting, visually appealing, and straightforward (no caption needed, no abbreviations except gene names). Please upload as a separate high resolution image file (tiff preferred). The technical requirements for the graphical abstracts are:
--size of the submitted image: 1200 pixels square at 300dpi
--font of the text: Arial 12-16 point (smaller fonts will not be legible online)

Graphical abstract included in the submission following editor’s guidelines.

-Please add an Abbreviated Summary (up to 50 words) of your paper that captures the main purpose and conclusions of the work. The summary should state the main results

written in the third person ('[1st author surname] et al. report that …') and conclusion. This summary will be used in the contents list online and should not contain any abbreviations other than those on the accepted abbreviations list and accepted gene/protein names, making it accessible to the non-specialist. Please upload it as a Word document. Allowed abbreviations: AIDS; ANOVA; AMPA; ATP; A,T,C,G; CNS; CSF; CT; DNA; ECG; EEG; EMG; GABA; HIV; MRI; NMDA; PET; PCR; RNA.

Abbreviated summary included in the submission.

Please include a paragraph about data availability in the 'Materials and Methods' section. We strongly encourage data sharing either as supplementary information or uploads to repositories which you can link in the paper.

We have already included a paragraph about data availability. The datasets have been uploaded onto PRIDE repository and will be made public after publication, for the moment editor and reviewers can access the datasets with the credentials included above.

Full- size and uncropped blots/gels are to be made available as Supplementary material.

Full size and uncropped blots are made available in the supplementary material.

To comply with our ethical standards, the appropriate checklist must be included (see Reporting Guideline section within 'Instructions to authors').

We are now including the STROBE checklist for observational studies.
